# Synthesis of Oxacalix[4]arene and the Study of Its Dynamic Behavior

**DOI:** 10.1021/acs.orglett.6c01571

**Published:** 2026-05-15

**Authors:** Michal Churý, Karla Hovorková, Radek Staník, Václav Eigner, Jan Sýkora, Pavel Lhoták

**Affiliations:** ‡ Department of Organic Chemistry, 52735 University of Chemistry and Technology Prague (UCTP), Technická 5, 166 28 Prague 6, Czech Republic; § Institute of Physics AS CR, v.v.i., Na Slovance 1999/2, 182 21 Prague 8, Czech Republic; † Department of Analytical Chemistry, UCTP, Technická 5, 166 28 Prague 6, Czech Republic

## Abstract

The preparation of a new member of the mixed-bridged calixarenes, monooxacalix[4]­arene, is described. The key step is the synthesis of an oxygen-bridged bisphenol, which was obtained by an Ullmann-type cross-coupling reaction using 2,2,6,6-tetramethylheptane-3,5-dione/CuI as a catalytic system in a microwave reactor. Final fragment condensation under high-dilution conditions allowed the isolation of oxacalix[4]­arene in 36% yield. The dynamic behavior of the new macrocycle was studied using VT NMR experiments, and the energy barriers of the basic dynamic processes (*cone*–*cone* interconversion and *flip*–*flop* motion of hydrogen bonds at the lower rim) were determined. Preliminary attempts to immobilize the macrocycle in a specific conformation revealed a surprising effect of the oxygen bridge on the conformational outcome of the alkylation reactions (*cone* vs *partial cone*).

## Graphical abstract

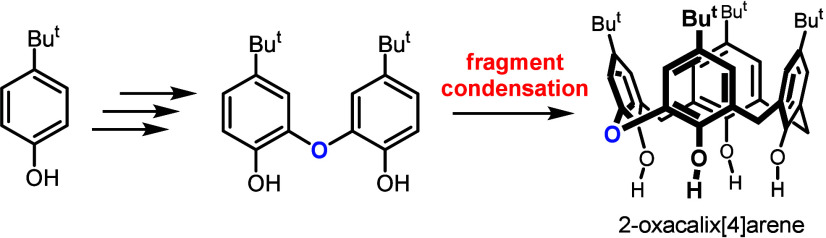

The calix­[*n*]­arene family^1^ represents a very popular group of macrocyclic compounds that play an irreplaceable role in modern supramolecular chemistry.^2^ A number of unique properties (for example, the preparation of macrocycles of various sizes in multigram quantities, almost unlimited possibilities of subsequent derivatization of the basic skeleton, and excellent complexation abilities depending on the derivatization) make these compounds very valuable building blocks in the design and synthesis of new receptors, self-assemblies, and other sophisticated supramolecular systems.^3^

Furthermore, in the case of calix[4]­arene **I** (Figure 1), there is the possibility of locking the three-dimensional structure in one of the four basic conformations/atropisomers (*cone*, *partial cone*, *1,2-alternate*, and *1,3-alternate*). Such fixed conformations with a precisely defined 3D structure can then be used as molecular scaffolds for the preparation of more elaborate functional systems.

**Figure 1:**
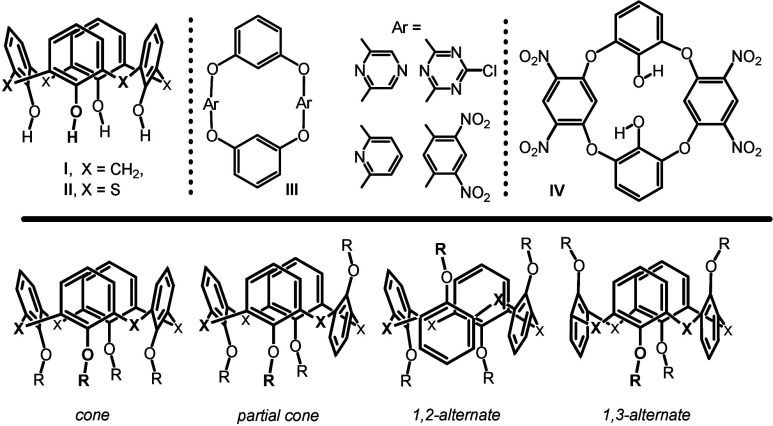
Calix­[4]­arene **I**, thiacalix[4]­arene **II** and typical general structures of oxacalix[4]­arenes **III** and **IV** (top). Four conformations/atropisomers of (thia)­calix[4]­arenes **I** and **II** (bottom).

The relatively well-established chemistry of classical calixarenes has been significantly innovated by introducing heteroatoms instead of CH_2_ linkages. Thus, the existence of four sulfur atoms^4^ in the so-called thiacalix[4]­arene **II** brings with it a whole range of new properties (different complexation ability, amended conformational preferences, new type of chemistry/derivatization, etc.)^5^ that are not available in the parent macrocycle **I** (Figure 1). Even replacing only some of the bridging groups (one or two) provides mixed-bridged systems^6^ with significantly different properties compared to the parent macrocycles.

There are a number of articles in the literature devoted to the so-called oxacalixarenes **III** and **IV** (Figure 1).^7^ These macrocycles are prepared by aromatic nucleophilic substitution of suitable starting building blocks (1,5-difluoro-^8^ or 1,5-dichloro-2,4-dinitrobenzene,^9^ cyanuric chloride,^10^ cyanohydrin,^11^ or perfluoroaromates^12^) with resorcinol, pyrogallol,^8a^ and their derivatives.^13^ These compounds exhibit a number of interesting properties that can be used in supramolecular chemistry, e.g., inherent chirality,^14^ complexation of anions,^15^ or detection of aromatic nitro compounds^16^ (explosives).

Although these structures resemble the aforementioned (thia)­calix[4]­arenes **I** and **II** in terms of the *meta*-linkage of the bridging groups, they cannot be considered “true” calixarenes. Unlike them, they cannot be fixed in a given conformation by simple substitution of the lower rim since they (fully or partially) lack phenolic OH groups. However, this significantly limits their use as molecular scaffolds, as the only available conformation for **III** and **IV** is usually *1,3-alternate*. In this context, it would be interesting to prepare oxygen-bridged systems that would exhibit the structure of real calixarenes. This Letter deals with the synthesis of monooxacalix[4]­arene as the first member of the mixed-bridged oxacalixarene family and its basic conformational and dynamic properties.

The advantage of classical calixarenes lies in their simple preparation from readily available materials,^1^ usually starting from *p*-*tert*-butylphenol. We therefore attempted to apply a similar approach to the synthesis of oxacalixarenes. As the simplest possible strategy, we chose the so-called fragment condensation,^17^ consisting of the final macrocyclization of appropriately substituted bisphenols.

The starting *p*-*tert*-butylphenol **1** was regioselectively formylated using a literature procedure^18^ to give aldehyde **2** in 96% yield (Scheme 1). This compound was alkylated by MeI/K_2_CO_3_ in MeCN, and the corresponding methoxy derivative **3** (89% yield) was subjected to a Baeyer–Villiger rearrangement.^19^ The use of sodium peroxodisulfate (Na_2_S_2_O_8_) as an oxidizing agent in aqueous MeCN at 55 °C led to the isolation of product **4** in 72% yield after column chromatography. The product was also alternatively obtained by vacuum distillation (52% yield), which might be more suitable for large-scale synthesis. The starting *tert*-butylphenol **1** was simultaneously converted to the corresponding methoxybromo derivative **6** via bromination^20^ (compound **5**, 80% yield) and subsequent alkylation (78% yield) with MeI/K_2_CO_3_ in DMF.

**Scheme 1:**
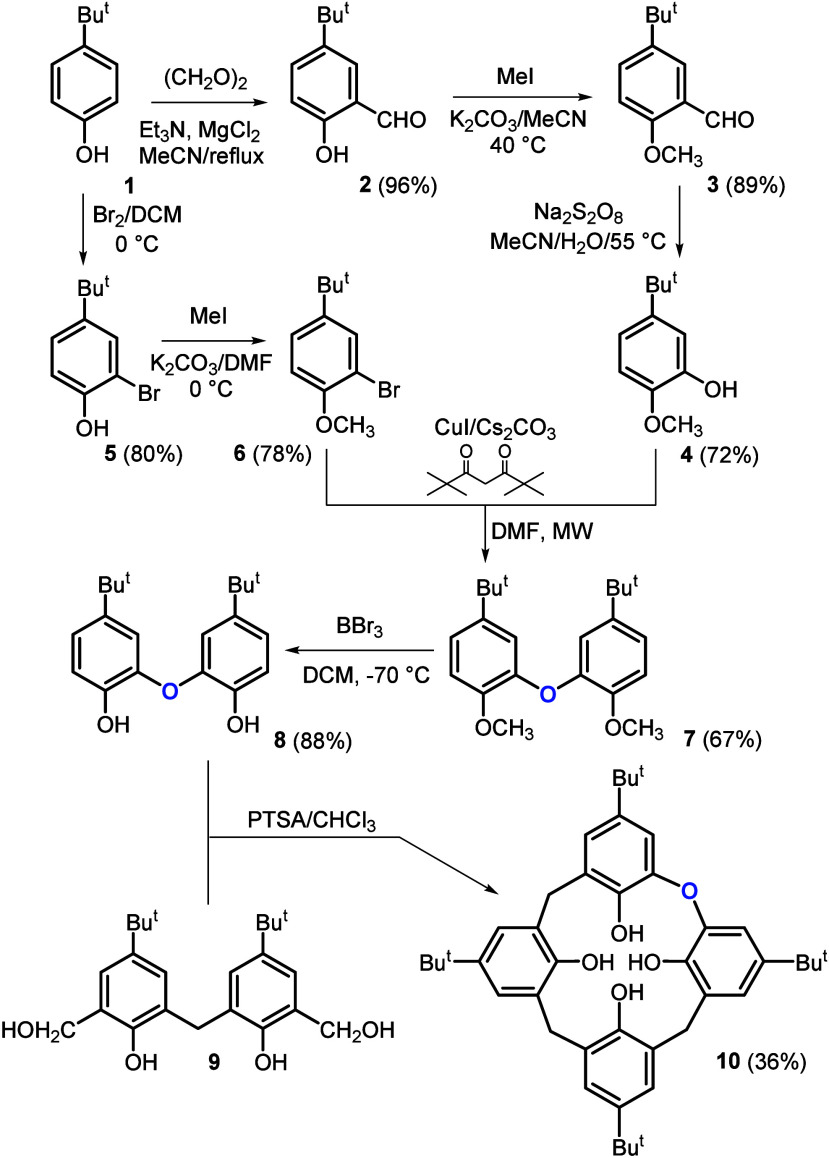
Synthesis of Monooxacalix[4]­arene

The key reaction of the entire synthesis is the synthesis of bisphenol **7** by condensation of compound **4** with compound **6**. Although many examples of diaryl ether synthesis can be found in the literature,^21^ none of them deal with similar structural motifs. We tested a procedure based on the use of the *N*,*N*-dimethyl glycine-promoted Ullmann coupling^22^ reaction (CuI 10 mol %, Cs_2_CO_3_/dioxane/90 °C/24 h). The ^1^H NMR of the crude reaction mixture indicated the formation of product **7** but with a very low conversion of the starting compounds (only about 15%). It is known that addition of 2,2,6,6-tetramethylheptan-3,5-dione (TMHD) forms a catalytic system which accelerates the Ullmann-type cross-coupling reactions^23^ under moderate conditions. Thus, a mixture of **4** and **6** was reacted with 10 mol % CuI and 10 mol % TMHD in DMF under various reaction conditions.

As can be seen from Table S1, conventional heating under reflux or heating in a sealed thick-walled flask only led to low yields of compound **7** (15% vs 33% yield after 24 h). The best results were ultimately achieved using microwave irradiation, where a significant reduction in reaction time (8 h) and the highest yields (67%) were achieved. Bisphenol **8** was readily obtained (in 88% yield) by dealkylation of phenolic groups with BBr_3_ in DCM at −78 °C.

This key building block was then condensed with bis­(hydroxymethylated) derivative **9**, prepared according to a literature procedure.^24^ The macrocyclization of blocks **8** and **9** was performed under pseudo-high-dilution conditions. Solutions of both reactants (1 equiv + 1 equiv, CHCl_3_) were added dropwise under reflux to a large volume of solvent containing PTSA (2 equiv) using a dual syringe pump over 4 h, and the reaction mixture was then stirred for another hour. Under these circumstances, oxacalix[4]­arene **10** was isolated in a 36% yield.

The HRMS (ESI^+^) spectrum showed a peak at *m*/*z* = 673.3862 perfectly corresponding to the value predicted for [**10** + Na]^+^ ion (*m*/*z* = 673.3864, C_43_H_54_O_5_Na). The ^1^H NMR spectrum of **10** (CD_2_Cl_2_, 600 MHz, 298 K) revealed two singlets for *tert*-butyl groups (1.195 and 1.203 ppm) together with four doublets with *meta* coupling constants (*J* ≈ 2.3 Hz) in the aromatic part of the spectrum (7.20, 7.07, 7.05, and 7.01 ppm). The broad OH signal at 10.01 ppm indicates strong hydrogen bonds at the lower rim, consistent with the *cone* conformation of the macrocycle in solution. Also, the ^13^C NMR spectrum (Figure S11) is fully consistent with the assumed *C*_*s*_ symmetry of **10**.

The use of new macrocyclic systems in supramolecular applications is always based on knowledge of their conformational and dynamic behavior. Therefore, oxacalixarene **10** was studied using VT ^1^H NMR techniques, both under heating (C_2_D_2_Cl_4_) and at low temperatures (CD_2_Cl_2_).

At ambient temperature, the ^1^H NMR spectrum of compound **10** (C_2_D_2_Cl_4_, 600 MHz) shows broadened signals of the three methylene bridges located at 4.25 and 3.53 ppm for the axial and equatorial CH bonds, respectively. Its broadening indicates a mutual exchange of these diastereotopic hydrogen atoms which is caused by an interconversion of two *cone* conformations (Figure 2). Lowering the temperature (267 K) resulted in two sets of sharp signals in a 2:1 ratio for each of the resonances. At elevated temperature (363 K), the CH_2_ signals collapsed into a single set of resonances at 3.96 and 3.92 keeping the 2:1 ratio due to the symmetry of the molecule. The ^1^H NMR spectra measured in CD_2_Cl_2_ in the range of 263–298 K were analyzed by Dynamic NMR Models (dNMR) in the Topspin software package, and the rate constants *k* were determined for each temperature. The activation free energy for the *cone*–*cone* interconversion (Δ*G* ^#^ = 61.5 kJ mol^–1^) was determined using Eyring equations. Surprisingly, this barrier is considerably lower than that for the parent *tert*-butylcalix­[4]­arene **I** (65.7 kJ mol^–1^),^25^ indicating a significant influence of the oxygen bridge.

**Figure 2:**
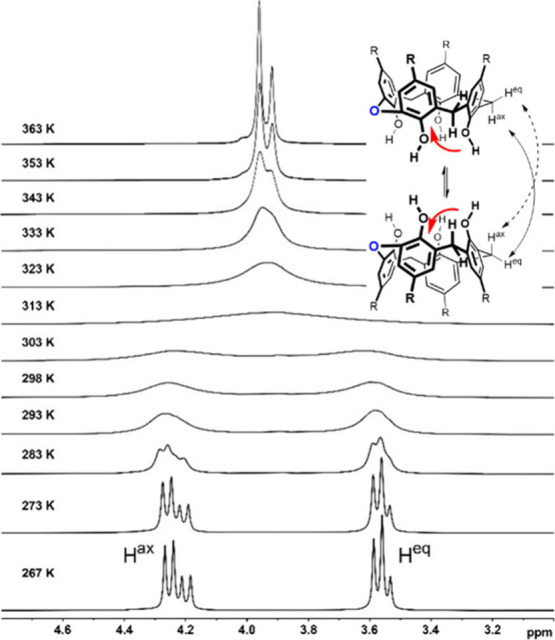
Partial VT ^1^H NMR spectra (C_2_D_2_Cl_4_, 500 MHz) of **10** showing the CH_2_ bridge area.

Furthermore, the signals of four phenolic OH groups (CD_2_Cl_2_) are collapsed into a broad singlet at rt. With decreasing temperature, the signal broadens and finally splits into four well-resolved resonances below 213 K (Figure 3). This dynamic behavior corresponds to a rapid change of the direction (sometimes called the *flip-flop* motion) of the cyclic hydrogen bond arrangement at the lower rim of calix[4]­arene. The Δ*G* ^#^ of 47.2 kJ mol^–1^ for this process was obtained by the line shape fitting within the temperature range 203–243 K using dNMR software. Comparison with the barrier reported for *tert*-butylcalix­[4]­arene **I** (Δ*G* ^#^_300K_ = 47.6 kJ·mol^–1^)^26^ suggests that the introduction of an oxygen atom does not have a major effect on weakening the circular hydrogen bond array on the lower rim.

**Figure 3:**
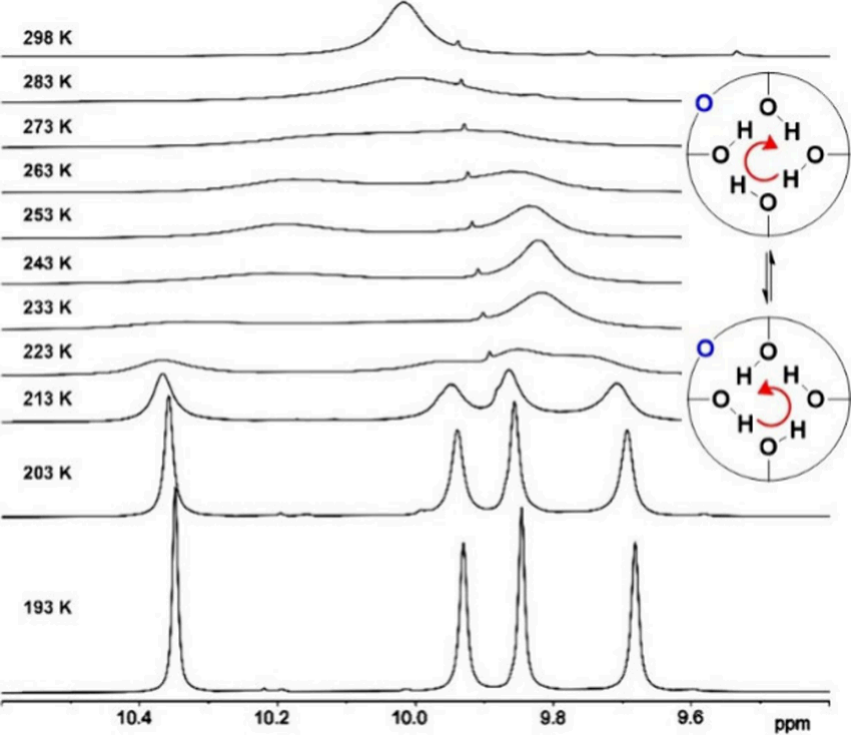
Partial VT ^1^H NMR spectra (CD_2_Cl_2_, 500 MHz) of **10** in the range of 193–253 K (*flip-flop* motion of the OH groups).

Final evidence of the structure of compound **10** was obtained by single-crystal X-ray structural analysis. The macrocycle crystallized (CHCl_3_) in a tetragonal crystal system, space group *P*4/*n*. As shown in Figure 4a, oxacalixarene adopts a *cone* conformation in the solid state, held together by a circular arrangement of hydrogen bonds at the lower rim. Unfortunately, as a result of the high symmetry of the molecule, the oxygen bridge is statistically distributed across all four positions with an occupancy of 25%. The final structure, therefore, represents only a statistically averaged molecule.

**Figure 4:**
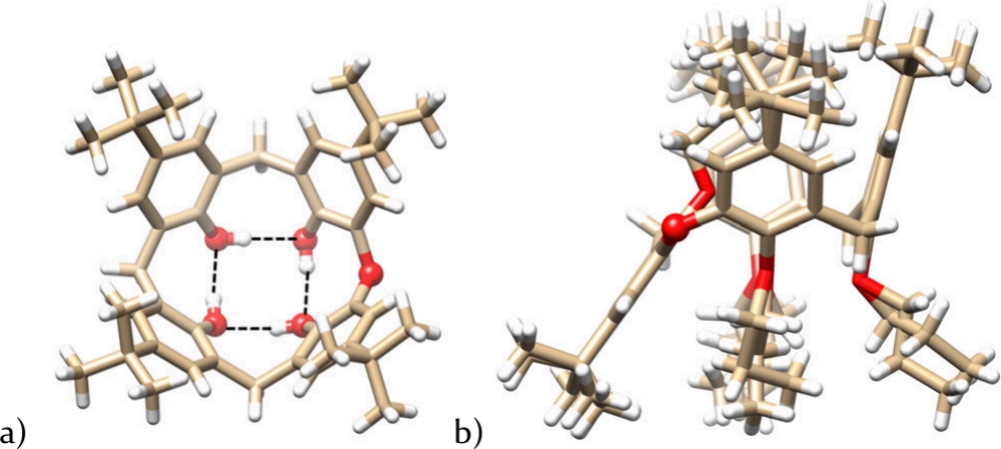
(a) Single-crystal X-ray structure of compound **10** (top-view) (one of the four equivalent positions of bridging O shown as a ball). (b) Single-crystal X-ray structure of compound **12** (side-view) (one of the two equivalent positions of bridging O shown as a ball).

The immobilization of the scaffold in one of the four basic conformations is perhaps the most attractive feature of calix[4]­arenes. Since the *cone* conformation is most commonly used for receptor design, we performed a preliminary study of fixing macrocycle **10** in this conformation. Compound **10** was alkylated under conditions known from the literature to selectively yield a *cone* conformation in a classical calix[4]­arene (PrI/NaH/DMF/rt) (Scheme 2). The crude reaction mixture contained two major products in a ratio of approximately 2:1 (NMR), and subsequent preparative TLC chromatography on silica gel afforded two peralkylated products **11** and **12** in 24% and 12% yield, respectively.

**Scheme 2:**
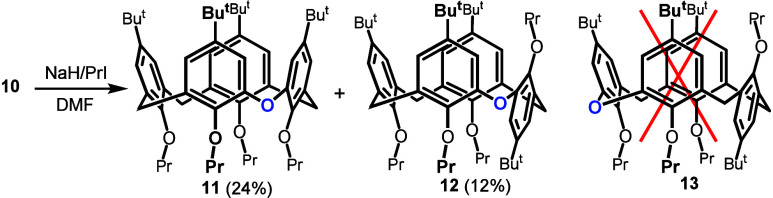
Alkylation of Monooxacalix[4]­arene **10**

The ^1^H NMR spectrum (CDCl_3_, 400 MHz, 298 K) of product **11** exhibits a set of two doublets at 4.44 ppm (1H) and 4.46 (2H) with geminal coupling constants of ^2^ *J* ≈ 12.5 Hz, typical for axial CH bonds of the methylene bridges of the *cone* conformation. Superposition of the equatorial CH counterparts (3H) forms a doublet at 3.10 ppm (^2^ *J* ≈ 12.5 Hz). Also, the presence of two *tert*-butyl groups (1.05 and 1.06 ppm) and two triplets of terminal methyls (0.94 and 0.98 ppm) is fully consistent with the predicted *cone* conformation (*C*_*s*_ symmetry).

The four signals of *tert*-butyl groups for byproduct **12/13** (1.15, 1.17, 1.19, and 1.32 ppm) indicated the absence of any symmetry elements and thus pointed to a *partial cone* conformation exhibiting an inherently chiral structure. The formation of the *paco* isomer itself is very unexpected because the reaction conditions are known to provide selectively the *cone* conformer in the parent calix[4]­arene **I**. Indeed, under the same reaction conditions, only the *cone* tetrapropoxy derivative of **I** was obtained (82% yield), while we were unable to detect (^1^H NMR) any presence of the *paco* isomer as a potential byproduct. This indicates the unique influence of the heteroatom bridge (oxygen) of the macrocycle on the conformational outcome of the alkylation reaction.

Furthermore, and perhaps even more interestingly, only one of the two theoretically possible *partial cone* conformers, **12** and **13**, is formed (Scheme 2). The splitting pattern of methylene bridges in the ^1^H NMR spectrum (2 × 2 doublets with typical coupling constants) undoubtedly corresponds to compound **12** possessing two methylene bridges with the *syn*-orientation of the three neighboring aromatic units. DFT calculations using the B3LYP functional and def2-TZVP basis set (see SI, p. 36) revealed a significant difference in the stability of both *partial cone* isomers (25.06 kJ/mol in favor of compound **12**), indicating that the formation of isomer **13** would be thermodynamically disadvantageous.

In addition, a significant upfield shift of one propoxy group (δ = 0.18 ppm for CH_2_ and 0.12 ppm for CH_3_ moieties) represents unprecedented extreme values, not previously observed in similar calix[4]­arene analogues. The 2D NOESY spectrum revealed steric interactions between the inverted propoxy group and the neighboring aromatic rings (Figure 5). It suggests that the terminal CH_2_–CH_3_ group enters deeper into the cavity of the macrocycle, which ultimately leads to its significant shielding.

**Figure 5:**
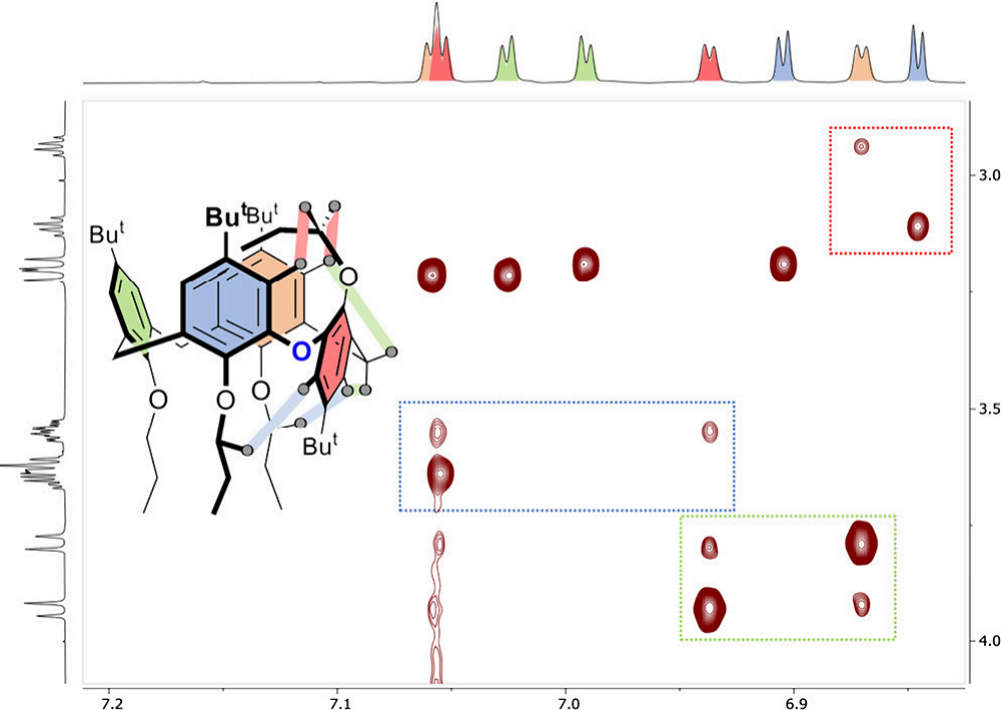
Section of a 2D NOESY spectrum of compound **12** (500 MHz, CDCl_3_, 298 K) showing a through-space interaction between the inverted propoxy group and adjacent aromatic rings (red rectangle), the interaction of the inverted aromatic ring with both nearby propoxy groups (blue), and the interactions between the methylene bridge and neighboring aromatics (green).

The structure and conformation of **12** were unambiguously confirmed by single crystal X-ray analysis (Figure 4b). The macrocycle crystallized in the monoclinic system, space group *P*2_1_/*c*, and adopted a *partial cone* conformation. If we define the main plane of the macrocycle by the four bridges, the inverted phenolic unit points into the cavity at an acute angle (interplanar angle Φ = 53.69°). This arrangement brings the terminal CH_2_CH_3_ group of the propyl moiety closer to the remaining three aromatic units, as indicated by the unusual ^1^H NMR shifts (see above). Unfortunately, even in this case it is not possible to separate the disordered positions of the oxygen and carbon bridge (probability 50%) due to the presence of a racemic mixture.

The preparation of a new member of the mixed-bridged calixarenes, monooxacalix[4]­arene **10**, is described. The synthesis of an oxygen-bridged bisphenol **7** as the key intermediate was achieved by Ullmann-type cross-coupling reaction using 2,2,6,6-tetramethylheptane-3,5-dione/CuI as a catalytic system in a microwave reactor. The dynamic behavior of the new macrocycle was studied using VT NMR experiments, and the energy barriers Δ*G* ^#^ of the basic dynamic processes (*cone*–*cone* interconversion and *flip*-*flop* motion of hydrogen bonds at the lower rim) were determined. Preliminary attempts to immobilize the macrocycle in a specific conformation revealed a surprising effect of the oxygen bridge on the conformational outcome of the alkylation reactions (*cone* vs *partial cone*). The potential use in host–guest chemistry and further applications of this new macrocyclic system are currently being investigated.

## Supplementary Material



## Data Availability

The data underlying this study are available in the published article and its online Supporting Information.

## Notes

Experimental details, copies of ^1^H, ^13^C NMR, and IR spectra and HRMS of new compounds, VT NMR measurements, X-ray crystallographic data, and DFT calculations (PDF)

## References

[1] a Gutsche, C. D. Calixarenes Revisited; Royal Society of Chemistry, 1998.

[2] a Steed, J. W. ; Atwood, J. L. Supramolecular chemistry, 3rd ed.; John Wiley & Sons, 2022.

[3] Mandolini, L. ; Ungaro, R. Calixarenes In Action; Imperial College Press, 2000.

[4] Kumagai H., Hasegawa M., Miyanari S., Sugawa Y., Sato Y., Hori T., Ueda S., Kamiyama H., Miyano S. (1997). Facile synthesis
of p-tert-butylthiacalix[4]­arene by the reaction of p-tert-butylphenol
with elemental sulfur in the presence of a base. Tetrahedron Lett..

[5] Kumar R., Lee Y. O., Bhalla V., Kumar M., Kim J. S. (2014). Recent developments of thiacalixarene
based molecular
motifs. Chem. Soc. Rev..

[6] Kortus D., Miksatko J., Kundrat O., Babor M., Eigner V., Dvorakova H., Lhotak P. (2019). Chemistry of 2,14-Dithiacalix[4]­arene:
Alkylation and Conformational Behavior of Peralkylated Products. J. Org. Chem..

[7] Maes W., Dehaen W. (2008). Oxacalix­[n]­(het)­arenes. Chem.
Soc. Rev..

[8] Csokai V., Kulik B., Bitter I. (2006). The First
Synthesis
of Functionalized Oxacalix[4]­crown Ethers. Supramol.
Chem..

[9] Yang Y., Xue M., Chen C.-F. (2010). Nanotoroidal
tubule assembled from a functionalized
oxacalix[4]­arene. CrystEngComm.

[10] Wang D.-X., Zheng Q.-Y., Wang Q.-Q., Wang M.-X. (2008). Halide Recognition
by Tetraoxacalix[2]­arene[2]­triazine
Receptors: Concurrent Noncovalent Halide−π and Lone-pair−π
Interactions in Host–Halide–Water Ternary Complexes. Angew. Chem., Int. Ed..

[11] Katz J. L., Geller B. J., Conry R. R. (2006). Synthesis of Oxacalixarenes Incorporating
Nitrogen Heterocycles: Evidence for Thermodynamic Control. Org. Lett..

[12] Kovtonyuk V. N., Gatilov Y. V., Salnikov G. E., Amosov E. V. (2019). Polyfluorinated
tetraoxacalixarenes and bicyclooxacalixarenes. Interaction of pentafluorobenzonitrile
with resorcinol, orcinol and tetrafluororesorcinol. J. Fluor. Chem..

[13] Katz J. L., Feldman M. B., Conry R. R. (2005). Synthesis
of Functionalized Oxacalix[4]­arenes. Org. Lett..

[14] Li Z., Zhang J., Zhu W., Wang T., Tang Y., Wang J. (2025). N-Heterocyclic carbene-catalyzed
enantioselective (dynamic) kinetic resolution for the assembly of
inherently chiral macrocycles. Chem. Sci..

[15] Wang D.-X., Wang M.-X. (2013). Anion−π
Interactions: Generality, Binding Strength, and Structure. J. Am. Chem. Soc..

[16] Panchal M., Kongor A., Athar M., Modi K., Patel C., Dey S., Vora M., Bhadresha K., Rawal R., Jha P. C. (2020). Structural
motifs of oxacalix[4]­arene for molecular recognition of nitroaromatic
explosives: Experimental and computational investigations of host-guest
complexes. J. Mol. Liq..

[17] Kortus D., Moravec O., Varga H., Churý M., Mamleev K., Čejka J., Dvořáková H., Lhoták P. (2025). Synthesis
of Monothiacalix[4]­arene Using the Fragment Condensation Approach. Molecules.

[18] Knight P. D., O’Shaughnessy P. N., Munslow I. J., Kimberley B. S., Scott P. (2003). Biaryl-bridged Schiff
base complexes of zirconium alkyls: synthesis
structure and stability. J. Organomet. Chem..

[19] Li W., Chen L.-L., Han K., Liu Z.-B., Luan Y.-S., Chen D.-T. (2019). Transition-metal-free
Baeyer–Villiger oxidation
of benzaldehydes to phenols using Na2S2O8. J.
Chem. Res..

[20] Sweetman B. A., Guiry P. J. (2018). Axially chiral tridentate isoquinoline
derived ligands
for diethylzinc addition to aldehydes. Tetrahedron.

[21] a Caron, S. ; McInturff, E. Nucleophilic Aromatic Substitution. In Practical Synthetic Organic Chemistry, 2020; pp 231–246.

[22] Ma D., Cai Q. N. (2003). N-Dimethyl
Glycine-Promoted Ullmann Coupling Reaction
of Phenols and Aryl Halides. Org. Lett..

[23] Buck E., Song Z. J., Tschaen D., Dormer P. G., Volante R. P., Reider P. J. (2002). Ullmann Diaryl Ether
Synthesis: Rate Acceleration by
2,2,6,6-Tetramethylheptane-3,5-dione. Org. Lett..

[24] Ingenfeld B., Straub S., Frömbgen C., Lützen A. (2018). Synthesis
of Monofunctionalized Calix[5]­arenes. Synthesis.

[25] Gutsche C. D., Bauer L. J. (1985). Calixarenes. 13. The conformational properties of calix[4]­arenes,
calix[6]­arenes, calix[8]­arenes, and oxacalixarenes. J. Am. Chem. Soc..

[26] Lang J., Deckerova V., Czernek J., Lhotak P. (2005). Dynamics of circular
hydrogen bond array in calix[4]­arene in a nonpolar solvent: a nuclear
magnetic resonance study. J. Chem. Phys..

