# Supplementary material for: Synthesis of Oxacalix[4]arene and the Study of Its Dynamic Behavior
Source: Org Lett. 2026 May 15;28(21):6722–7. doi: 10.1021/acs.orglett.6c01571 (PMC13220344; doi:10.1021/acs.orglett.6c01571)
Supplement: Supplementary file 1 [file ol6c01571_si_001.pdf]

Supporting Information

Synthesis of Oxacalix[4]arene and the Study of Its Dynamic Behaviour

Michal Churý,‡ Karla Hovorková,‡ Radek Staník,‡ Václav Eigner,§ Jan Sýkora,†
and Pavel Lhoták‡*

‡ Department of Organic Chemistry, University of Chemistry and Technology Prague (UCTP), Technická 5, 166 28, Prague 6, Czech Republic.
§ Institute of Physics AS CR, v.v.i., Na Slovance 1999/2, 182 21, Prague 8, Czech Republic.
† Department of Analytical Chemistry, UCTP, Technická 5, 166 28, Prague 6, Czech Republic.

Table of Content

1. General information 2
2. Experimental procedures and characterization 2
3. Spectral characterization of compounds 7
4. Variable temperature NMR spectra 25
5. Crystallographic data 31
6. Single crystal X-ray structures 33
7. Computational results – energy minimized alkylation products 36

1. General information

All chemicals were purchased from commercial sources and used without further purification. Acetone was dried and distilled using conventional methods, THF, DMF and CH_3CN were dried using column solvent purification system PureSolv MD7 (Inert). Melting points were measured on Heiztisch Mikroskop Polytherm A (Wagner & Munz) and they are not corrected. ^{1}H, $^{13}C\{^{1}H\}$, APT, 2D and VT spectra were measured on JEOL JNM-ECZL400G operating at 399.78 MHz for ^{1}H and 100.54 MHz for ^{13}C, another JEOL JNM-ECZL400G operating at 399.38 MHz for ^{1}H, Bruker Avance III 500 operating at 500.13 MHz for ^{1}H and 125.77 MHz for ^{13}C and Bruker Avance III 600 operating at 600.13 MHz for ^{1}H and 150.90 MHz for ^{13}C. Chemical shifts are given in δ-units (ppm) and are referenced to solvent signal. Structural assignments were made with additional information from gCOSY, gHSQC, and gHMBC experiments. IR spectra were measured on FTIR spectrometer Nicolet iS50 ABX with diamond ATR module. The measurement parameters: spectral range 4000 – 400 cm^{-1}, resolution 4 cm^{-1}, 64 spectral accumulations. ESI HRMS spectra were measured on Q-TOF (Micromass) spectrometer. Substance purities and courses of the reactions were monitored by thin layer chromatography (TLC) using silica gel 60 F_{254} on aluminium-backed sheets (Merck) and analysed at 254 and 365 nm. Preparative TLC was carried out on self-prepared plates using silica gel 60 GF_{254} for thin-layer chromatography (Merck) on glass tables.

2. Experimental procedures and characterization

5-*tert*-Butyl-2-hydroxybenzaldehyde (2)
The starting compound was prepared according to a previously published procedure.[(S1)]

5-*tert*-Butyl-2-methoxybenzaldehyde (3)
Compound **1** (9.75g, 57 mmol) was mixed with 65 ml of dry acetonitrile and then 16 g (114.5 mmol) of K_2CO_3 was added. The mixture was heated using a heating mantle at 40 °C for 30 min and then 10.65 ml of methyl iodide was added. The mixture was then stirred at 40 °C for another 7 days. Approximately half of the solvent was evaporated *in vacuo* and 100 ml of 5M HCl was added. The mixture was extracted 3 times with dichloromethane and the extracts were washed with 100 ml of brine and dried over $MgSO_4$. After evaporation of the solvent, 9.8 g (89 %) of yellow liquid was obtained. Analytical data were identical to those previously reported.[(S2)]

5-*tert*-Butyl-2-methoxyphenol (4)
Procedure used was a modified procedure from literature.[(S3)] Compound **2** (9.0 g, 46.5 mmol), $Na_2S_2O_8$ (16.8 g, 70.5 mmol), 450 ml of acetonitrile and 9 ml of water were added to 100ml flask. The mixture was heated using a heating mantle and stirred for 24 hours at 55 °C, then filtered and the solvent from the filtrate was evaporated. The liquid residue can be purified by a vacuum distillation. The fraction with boiling point of 90 °C at 9 torr was collected to obtain 4.4 g (52 %) of product as an orange liquid. Alternatively, a purification by column chromatography can be used. Separation using silica gel as a stationary phase and ethyl acetate:cyclohexane 1:4 as eluent yielded 6.1 g (72 %) of the product. All analytical data are consistent with the literature.[(S3)]

2-Brom-4-*tert*-butylphenol (5)
The compound was synthesized by modified procedure from the literature.[(S4)] 4-*tert*-Butylphenol (20.1 g, 134 mmol) was dissolved in 200 ml of dichloromethane. The solution was cooled down using an ice bath and a solution of 22.4 g (7 ml, 140 mmol) of bromine in 50 ml of dichloromethane was added dropwise. The mixture was stirred for another 2 h and the reaction was quenched by adding 50 ml of saturated $Na_2S_2O_3$. Two phases were separated and the aqueous phase was extracted 3 times

with 100 ml of dichloromethane. The extracts were washed with 200 ml of brine and dried over $MgSO_4$. After evaporation of the solvent, the product was distilled of at 75 °C at 1 torr. The reaction yielded 24 g (78 %) of yellowish liquid. All analytical data are consistent with the literature.[S4]

2-Brom-4-*tert*-butyl-1-methoxybenzene (6)
Compound **4** (20 g, 86.9 mmol) and K_2CO_3 (24.1 g, 174 mmol) were suspended in 300 ml of N,N-dimethylformamide. After stirring at 40 °C for 30 min, the solution had blue colour. Then, 11 ml (25 g, 180 mmol) of methyl iodide was added and the colour changed to light yellow. The mixture was heated using a heating mantle and stirred at 40 °C for another 6 days, another portion of methyl iodide (5.5 ml, 12.5 g, 88 mmol) was added and the stirring continued for another day. The reaction was quenched by adding 100 ml of 5M HCl and the mixture was extracted 3 times with 100 ml of chloroform. The extracts were washed with brine and dried over $MgSO_4$. After evaporating of the solvent *in vacuo*, the mixture was separated by distillation under reduced pressure. The reaction yielded 16.4 g (78%) of yellow liquid with boiling point of 105 °C at 1 torr. Analytical data were identical to those previously reported.[S5]

2,2’-oxybis(4-*tert*-butyl-1-methoxybenzene) (7)
The synthesis was based on C-O coupling published.[S6]

Table S1. Optimization of the synthesis of compound **7** by cross coupling reaction of **4** and **6**. A mixture of phenol **4** (3.22 mmol), compound **6** (2.07 mmol), Cs_2CO_3(4.28 mmol), 10 mol% CuI and 10 mol% TMHD in DMF (2 ml) was stirred under the indicated reaction conditions.

Temp.	Time	Reaction arrangement	Yield
120 °C	24 h	flask under reflux (Ar atm.)	15%
140 °C	72 h	flask under reflux (Ar atm.)	21%
110 °C	24 h	closed pressure tube	33%
120 °C	48 h	closed pressure tube	40%
120 °C<br>140 °C	6 h<br>2 h	microwave heating/microwave tube	64%
140 °C	8 h	microwave heating/microwave tube	67%

*Detailed and optimized synthesis of **7***

Compounds **4** (580 mg, 3.22 mmol) and **6** (500 mg, 2.07 mmol), Cs_2CO_3 (1.4 g, 4.28 mmol and 41 mg CuI (0.207 mmol) were mixed together and then 0.045 ml of 2,2,6,6-tetramethylheptane-3,5-dione and 2 ml of dry N,N-dimethylformamide were added. The mixture was then microwaved at 140 °C for 8 hours. The reaction was let to cool down and 10 ml of 5M HCl was added. The mixture was extracted 3 times with 10 ml of dichloromethane and the extracts were washed with brine and dried on $MgSO_4$. After evaporation of the solvent, the mixture was separated by column chromatography on silica gel using dichloromethane:cyclohexane 3:2 as an eluent. White powder (0.47 g, 67 %) was obtained.
M.p. 140.1 - 149.3 °C.
^{1}H NMR ($CDCl_3$, 400 MHz, 298 K) δ (ppm): 7.04 (dd, 2H, *J*= 8.5, 2.4 Hz, Ar-*H*), 6.90 (d, 2H, *J*= 8.5 Hz, Ar-*H*), 6.88 (d, 2H, *J*= 2.4 Hz, Ar-*H*), 3.85 (s, 6H, -OCH_3), 1.21 (s, 18H, -CH_3).
^{13}C{^{1}H} NMR (101 MHz, $CDCl_3$, 298 K) δ (ppm): 148.3, 145.4, 144.1, 119.9, 116.4, 112.0, 56.2, 34.3, 31.6.
IR (ATR) ν (cm^{-1}): 3002, 2961, 2903, 2867,2837, 1611, 1575, 1512, 1463, 1406.
HRMS (ESI) *m/z*: $[M + Na]^+$ Calcd for $C_{22}H_{30}O_3Na$ 365.2087; Found 365.2092.

2,2'-Oxybis(4-*tert*-butylphenol) (8)

Under argon atmosphere, dimer **6** (0.42 g, 1.228 mmol) was dissolved in 58 ml of dry dichloromethane. The mixture was cooled down to -78 °C and 6.15 ml of 1M solution of BBr_3 in dichloromethane was added dropwise. The mixture was stirred for 30 min and then let warm up to room temperature. After adding 70 ml of 1M HCl, the crude mixture was extracted 3 times with 70 ml of dichloromethane, extracts were washed with brine (100 ml) and dried over $MgSO_4$. After evaporation, white powder (0.34 g, 88 %) was obtained.
M.p. 64.3-67.2 °C.
^{1}H NMR ($CDCl_3$, 399 MHz, 298 K) δ (ppm): 7.06 (dd, 2H, *J* = 8.4, 2.2 Hz, Ar-*H*), 6.98 (d, 2H, *J* = 8.4 Hz, Ar-*H*), 6.89 (d, 2H, *J* = 2.1 Hz, Ar-*H*), 5.42 (s, 2H, Ar-O*H*), 1.21 (s, 18H, -CH_3).
^{13}C{^{1}H} NMR (101 MHz, $CDCl_3$, 298 K) δ (ppm): 144.7, 144.1, 142.8, 121.5, 115.8, 115.2, 34.5, 31.6.
IR (ATR) ν (cm^{-1}): 3246, 2961, 2935, 2910, 2869, 1605, 1590, 1512, 1463, 1419, 1362.
HRMS (ESI) *m/z*: $[M + Na]^+$ Calcd for $C_{20}H_{26}O_3Na$ 337.1774; Found 337.1775, $[M - H]^-$ Calcd for $C_{20}H_{25}O_3$ 313.1809; Found 313.1809.

2,2'-Methylenebis(4-*tert*-butyl-phenol)
This compound was synthesized using a published procedure from commercially available 2,4-di-*tert*-butylphenol.[S7]

6,6'-Methylenebis(4-*tert*-butyl-2-hydroxymethylphenol) (9)
This compound was prepared by direct hydroxymethylation from **8** using a published procedure.[S8]

5,11,17,23-Tetra-*terc*-butyl-2-oxacalix[4]arene-25,26,27,28-tetraol (10)
Solutions of **7** (328 mg, 1.044 mmol) and **9** (389 mg, 1.044 mmol) in 40 ml of dry chloroform each were prepared and using a linear dispenser added to a boiling suspension of *p*-toluenesufonic acid monohydrate (400 mg, 2.088 mmol) in 200 ml of dry chloroform during 4 hours. Heating mantle was used. After the addition was complete, the mixture was refluxed for another 1 hour. After the mixture was cooled down to room temperature, it was washed twice with water and dried over $MgSO_4$. The solvent was evaporated using a rotovap and triturated with methanol, yielding 0.245 g (36 %) of white powder.
M.p. >300 °C
^{1}H NMR ($CDCl_3$, 399 MHz, 298 K) δ (ppm): 10.37 (s, 2H, Ar-O*H*), 7.24 (s, 2H, Ar-O*H*), 7.16 (d, 2H, *J* = 2.0 Hz, Ar-*H*), 7.03 (bs, 4H, Ar-*H*), 6.95 (d, 2H, *J* = 2.2, Ar-*H*), 4.24 (bs, 3H, Ar-CH_2-Ar), 3.52 (bs, 3H, Ar-CH_2-Ar), 1.20 (s, 18H, -CH_3), 1.19 (s, 18H, -CH_3).
^{13}C{^{1}H} NMR ($CDCl_3$, 101 MHz, 298 K) δ (ppm): 147.8, 146.9, 144.7, 144.4, 142.2, 129.0, 127.8, 127.6, 126.2, 126.1, 123.6, 118.9, 34.3, 34.1, 32.8, 32.5, 31.5, 31.3.
IR (ATR) ν (cm^{-1}): 3129, 2957, 2906, 2868, 1605, 1589 1485, 1462, 1391, 1363, 1301.
HRMS (ESI) *m/z*: $[M + Na]^+$ Calcd for $C_{43}H_{54}O_5Na$ 673.3864; Found 673.3862.

5,11,17,23-Tetra-*terc*-butyl-25,26,27,28-tetrapropoxy-2-oxacalix[4]arene (11 and 12)

In 1 ml of dry N,N-dimethylformamide, 50 mg of **10** (0.0769 mmol) was dissolved. The solution was cooled down to 0 °C and 26 mg of 60% dispersion of NaH in mineral oil (0.645 mmol) was added. The mixture was stirred for 20 min and 92 µl of 1-iodopropane (0.938 mmol) was added. The reaction mixture was stirred overnight at room temperature and 10 ml of 1M HCl was added. After extracting 3 times with 10 ml of dichloromethane, drying over $MgSO_4$ and evaporation of the solvent, the mixture was separated using preparative TLC on silica gel, using cyclohexane:dichloromethane 9:1 as eluent. The chromatography yielded 12 mg of *cone* derivative **11** (22 %) and 10 mg of *partial cone* derivative **12** (16 %), both white crystalline solids.

*Data for **11**.*

M.p. 192.7 - 206.8 °C.

^{1}H NMR (500 MHz, CD_2Cl_2, 298 K) δ (ppm): 6.81 (d, 2H, *J* = 2.5 Hz, Ar-*H*), 6.76 (d, 2H, *J* = 2.5 Hz, Ar-*H*), 6.73 (d, 2H, *J* = 2.4 Hz, Ar-*H*), 6.71 (d, 2H, *J* = 2.4 Hz, Ar-*H*), 4.48 (d, 2H, *J* = 12.4 Hz, Ar-CH_2-Ar), 4.46 (d, 1H, *J* = 12.5 Hz, Ar-CH_2-Ar), 4.34 (td, *J* = 10.0, 5.6 Hz, 2H, -OCH_2-), 3.88 (td, *J* = 9.8, 5.9 Hz, 2H, -OCH_2-), 3.83 (t, *J* = 7.6 Hz, 4H, -OCH_2-), 3.13 (d, *J* = 12.5 Hz, 2H, Ar-CH_2-Ar), 3.12 (d, *J* = 12.6 Hz, 1H, Ar-CH_2-Ar), 2.05-1.88 (m, 6H, -CH_2-), 1.82 (dddd, 2 H, *J* = 13.2, 9.6, 7.5, 5.9 Hz, -CH_2-), 1.08 (s, 18H, -CH_3), 1.07 (s, 18H, -CH_3), 1.02 (t, 6H, *J* = 7.4 Hz, -CH_3), 0.97 (t, 6H, *J* = 7.5 Hz, -CH_3).

^{13}C{^{1}H} NMR (101 MHz, CD_2Cl_2, 298 K) δ (ppm): 154.4, 151.9, 148.3, 144.7, 144.6, 136.4, 134.3, 134.1, 125.41, 125.36, 122.5, 118.3, 77.6, 76.9, 34.2, 34.1, 31.8, 31.6, 31.5, 31.1, 23.8, 23.2, 10.6, 10.5.

IR (ATR) ν (cm^{-1}): 2961, 2927, 2876, 1739, 1606, 1569, 1483, 1468, 1412, 1389, 1361.

HRMS (ESI) *m/z*: $[M + Na]^+$ Calcd for $C_{55}H_{78}O_5Na$ 841.5742; Found 841.5734.

*Data for **12**.*

M.p. = 221.3 - 227.1 °C.

^{1}H NMR (600 MHz, $CDCl_3$, 298 K) δ (ppm): 7.01 – 6.99 (m, 2H, Ar-*H*), 6.97 (d, *J* = 2.6 Hz, 1H, Ar-*H*), 6.94 (d, *J* = 2.5 Hz, 1H, Ar-*H*), 6.88 (d, *J* = 2.5 Hz, 1H, Ar-*H*), 6.85 (d, *J* = 2.5 Hz, 1H, Ar-*H*), 6.81 (d, *J* = 2.5 Hz, 1H, Ar-*H*), 6.79 (d, *J* = 2.4 Hz, 1H, Ar-*H*), 4.34 (d, 1H, *J* = 12.0 Hz, Ar-CH_2-Ar), 4.33 (d, 1H, *J* = 12.0 Hz, Ar-CH_2-Ar), 3.88 (d, *J* = 16.5 Hz, 1H, Ar-CH_2-Ar), 3.73 (d, *J* = 16.6 Hz, 1H, Ar-CH_2-Ar), 3.65 – 3.44 (m, 6H, -OCH_2-), 3.16 (d, *J* = 12.2 Hz, 1H, Ar-CH_2-Ar), 3.14 (d, *J* = 11.9 Hz, 1H, Ar-CH_2-Ar), 3.08 – 3.01 (m, 1H, -OCH_2-), 2.91 – 2.84 (m, 1H, -OCH_2-), 1.75 – 1.61 (m, 4H, -CH_2-), 1.52 – 1.11 (m, 2H, -CH_2-), 1.31 (s, 9H, -CH_3), 1.17 (s, 9H, -CH_3), 1.16 (s, 9H, -CH_3), 1.13 (s, 9H, -CH_3), 0.85 (t, 3H, *J* = 7.5 Hz, -CH_3), 0.82 (t, 3H, *J* = 7.5 Hz, -CH_3), 0.78 (t, 3H, *J* = 7.6 Hz, -CH_3), 0.18-0.03 (m, 5H, -C*H*2-C*H*3).

^{13}C{^{1}H} NMR (101 MHz, CD_2Cl_2, 298 K) δ (ppm): 154.7, 153.4, 150.2, 149.4, 147.2, 145.9, 145.5, 144.9, 144.8, 144.3, 136.9, 135.0, 134.7, 134.4, 133.8, 132.0, 125.82, 125.76, 125.16, 125.11, 122.7, 121.5, 116.0, 115.0, 77.7, 75.9, 75.7, 72.7, 39.2, 34.34, 34.28, 34.2, 34.1, 31.69, 31.67, 31.63, 31.58, 31.0, 23.6, 23.2, 22.9, 21.9, 10.6, 10.4, 10.2, 9.9.

APT NMR (151 MHz, $CDCl_3$, 298 K) δ (ppm): 154.52, 153.17, 149.93, 149.17, 146.95, 145.66, 145.27, 144.68, 144.57, 144.14, 136.55, 134.77, 134.31, 134.08, 133.51, 131.75, 125.55, 125.49, 124.87, 124.84, 122.40, 121.17, 115.76, 114.84, 77.43, 75.64, 75.46, 72.48, 39.16, 34.23, 34.16, 34.07, 34.00, 31.72, 31.68, 31.64, 31.60, 30.94, 30.93, 23.33, 23.00, 22.62, 21.66, 10.55, 10.39, 10.21, 9.82.

IR (ATR) ν (cm^{-1}): 2958, 2931, 2873, 1737, 1603, 1573, 1484, 1468, 1410, 1387, 1361, 1310.

HRMS (ESI) *m/z*: $[M + Na]^+$ Calcd for $C_{55}H_{78}O_5Na$ 841.5742; Found 841.5733; $[M + K]^+$ Calcd for $C_{55}H_{78}O_5K$ 857.5481; Found 857.5466.

(S1) Knight, P. D.; O'Shaughnessy, P. N.; Munslow, I. J.; Kimberley, B. S.; Scott, P., Biaryl-bridged Schiff base complexes of zirconium alkyls: synthesis structure and stability. *J. Organomet. Chem.* **2003,** *683* (1), 103-113.
(S2) Zhang, Z.; Chen, Y.-A.; Hung, W.-Y.; Tang, W.-F.; Hsu, Y.-H.; Chen, C.-L.; Meng, F.-Y.; Chou, P.-T., Control of the Reversibility of Excited-State Intramolecular Proton Transfer (ESIPT) Reaction: Host-Polarity Tuning White Organic Light Emitting Diode on a New Thiazolo[5,4-d]thiazole ESIPT System. *Chem. Mater.* **2016,** *28* (23), 8815-8824.
(S3) Li, W.; Chen, L.-L.; Han, K.; Liu, Z.-B.; Luan, Y.-S.; Chen, D.-T., Transition-metal-free Baeyer–Villiger oxidation of benzaldehydes to phenols using $Na_2S_2O_8$. *J. Chem. Res.* **2019,** *43* (3-4), 115-118.
(S4) Sweetman, B. A.; Guiry, P. J., Axially chiral tridentate isoquinoline derived ligands for diethylzinc addition to aldehydes. *Tetrahedron* **2018,** *74* (38), 5567-5581.
(S5) Li, K.; Guan, X.; Ma, C.-W.; Lu, W.; Chen, Y.; Che, C.-M., Blue electrophosphorescent organoplatinum(ii) complexes with dianionic tetradentate bis(carbene) ligands. *Chem. Commun.* **2011,** *47* (32), 9075-9077.
(S6) Buck, E.; Song, Z. J.; Tschaen, D.; Dormer, P. G.; Volante, R. P.; Reider, P. J., Ullmann Diaryl Ether Synthesis: Rate Acceleration by 2,2,6,6-Tetramethylheptane-3,5-dione. *Org. Lett.* **2002,** *4* (9), 1623-1626.
(S7) Churý, M.; Petrů, T.; Staník, R.; Sýkora, J.; Eigner, V.; Lhoták, P., Newcomer to the Calixarene Family: Synthesis and Characterization of Selenacalix[4]arene. *Org. Lett.* **2025,** *27* (50), 13722-13727.
(S8) Kim, S. Y.; No, K., Synthesis of calixsalen : A route to azacalixarene analogue. *Bull. Korean Chem. Soc.* **2007,** *28* (2), 315-318.

3. Spectral characterization of compounds

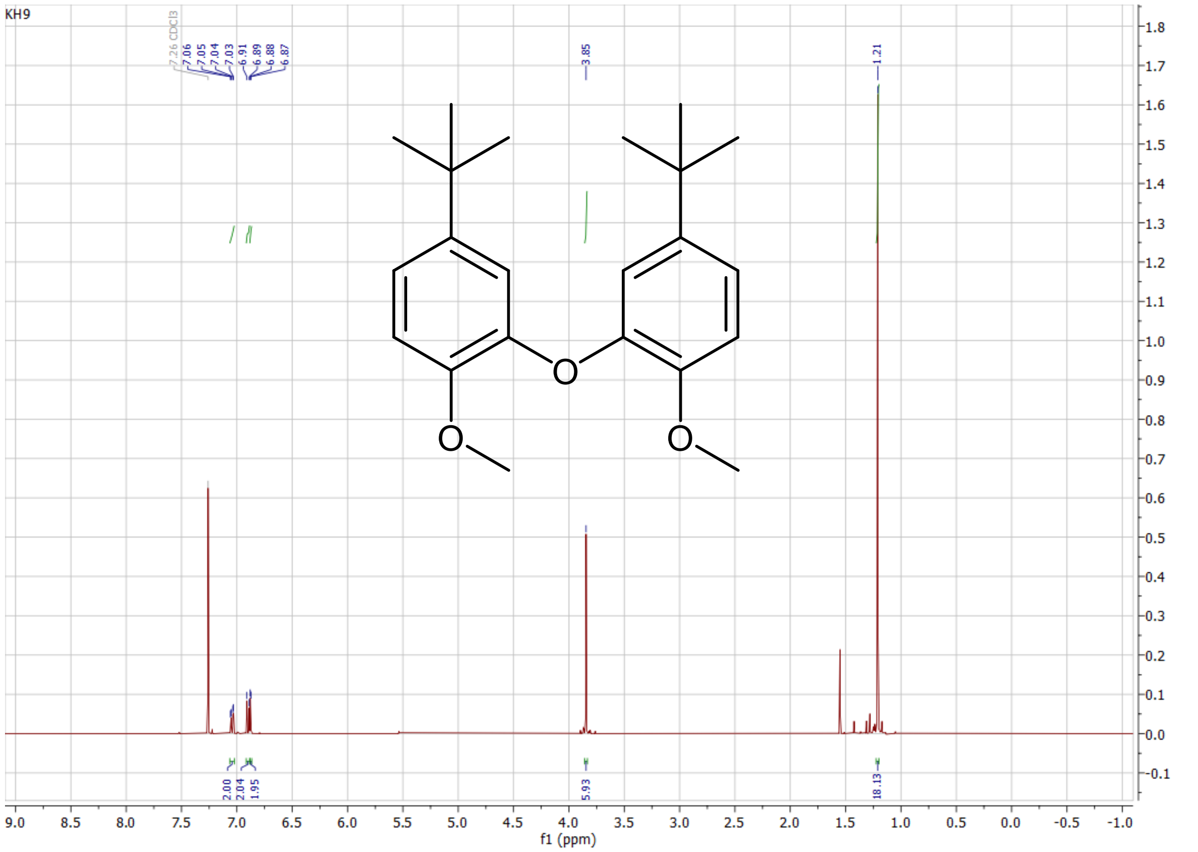

Figure S1: ^1H NMR spectrum of compound **7** in $CDCl_3$ (400 MHz)

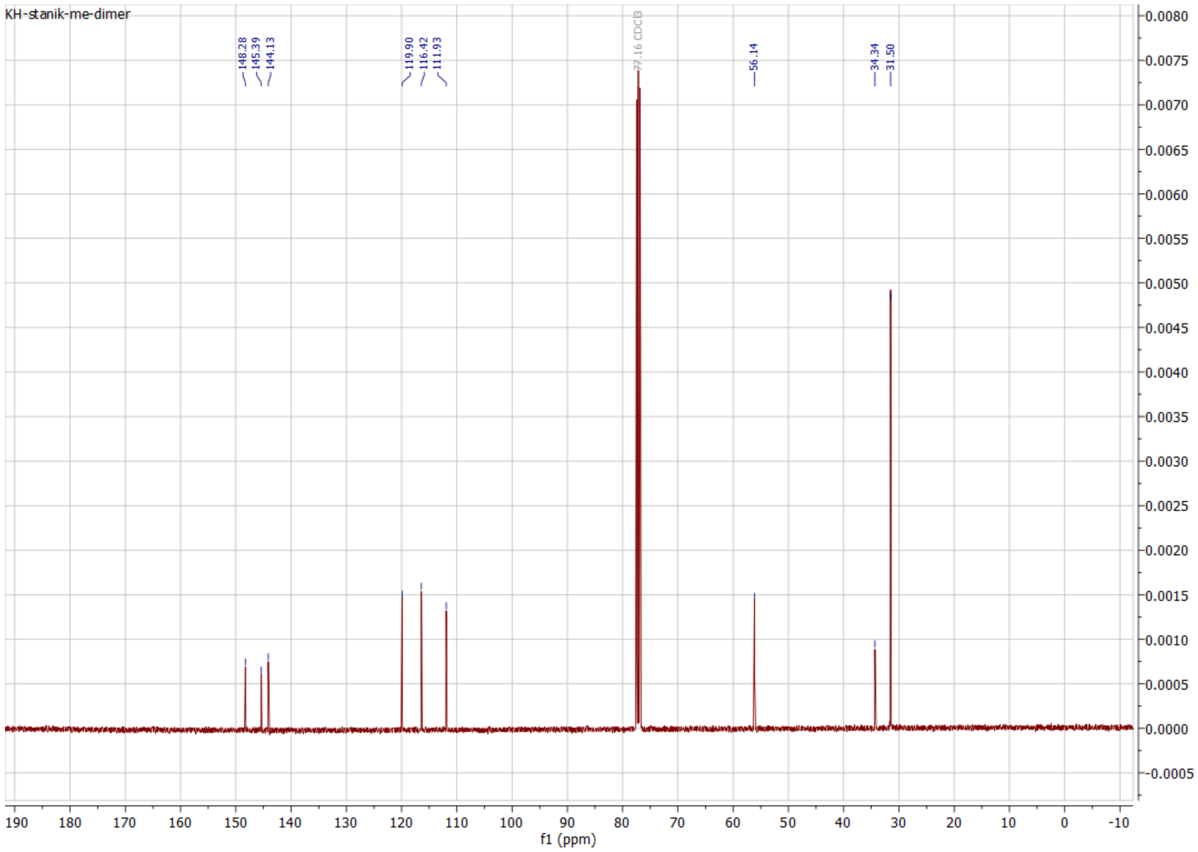

Figure S2: $^{13}C\{^1H\}$ NMR spectrum of compound **7** in $CDCl_3$ (101 MHz)

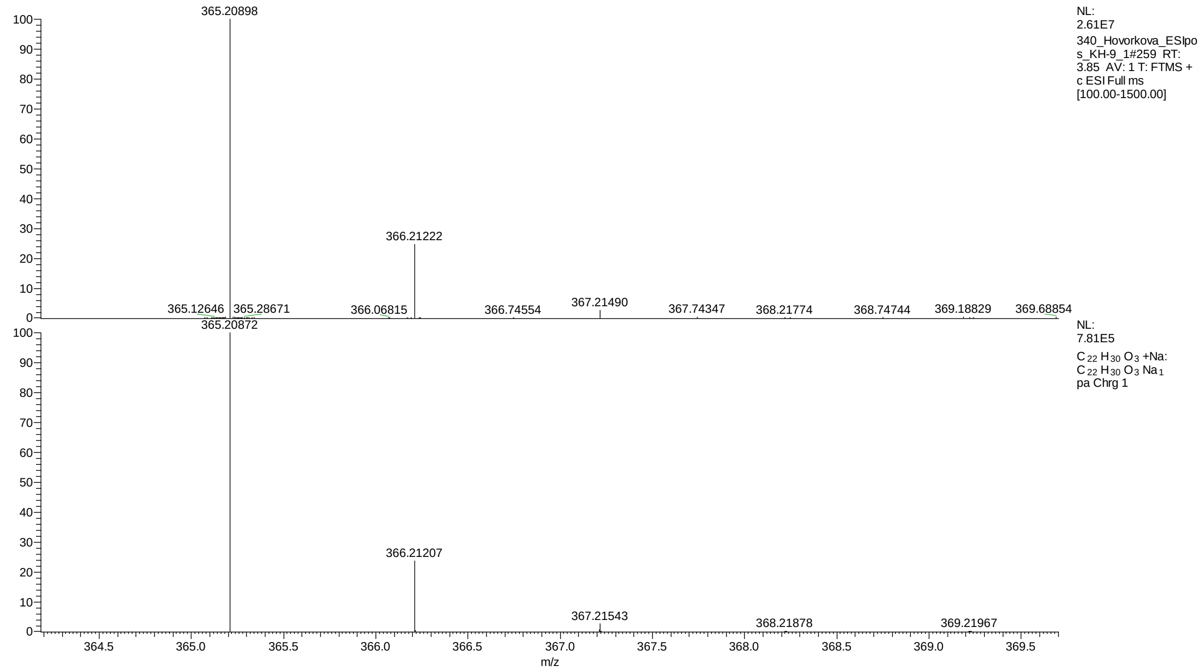

Figure S3: HRMS spectrum of compound **7**

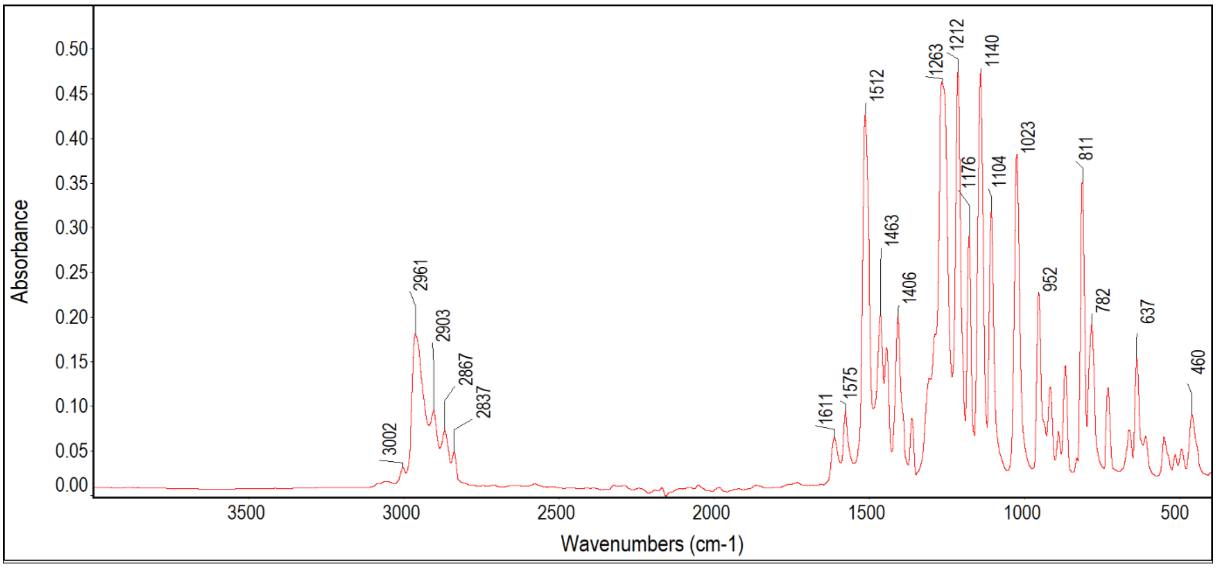

Figure S4: IR spectrum of compound **7**

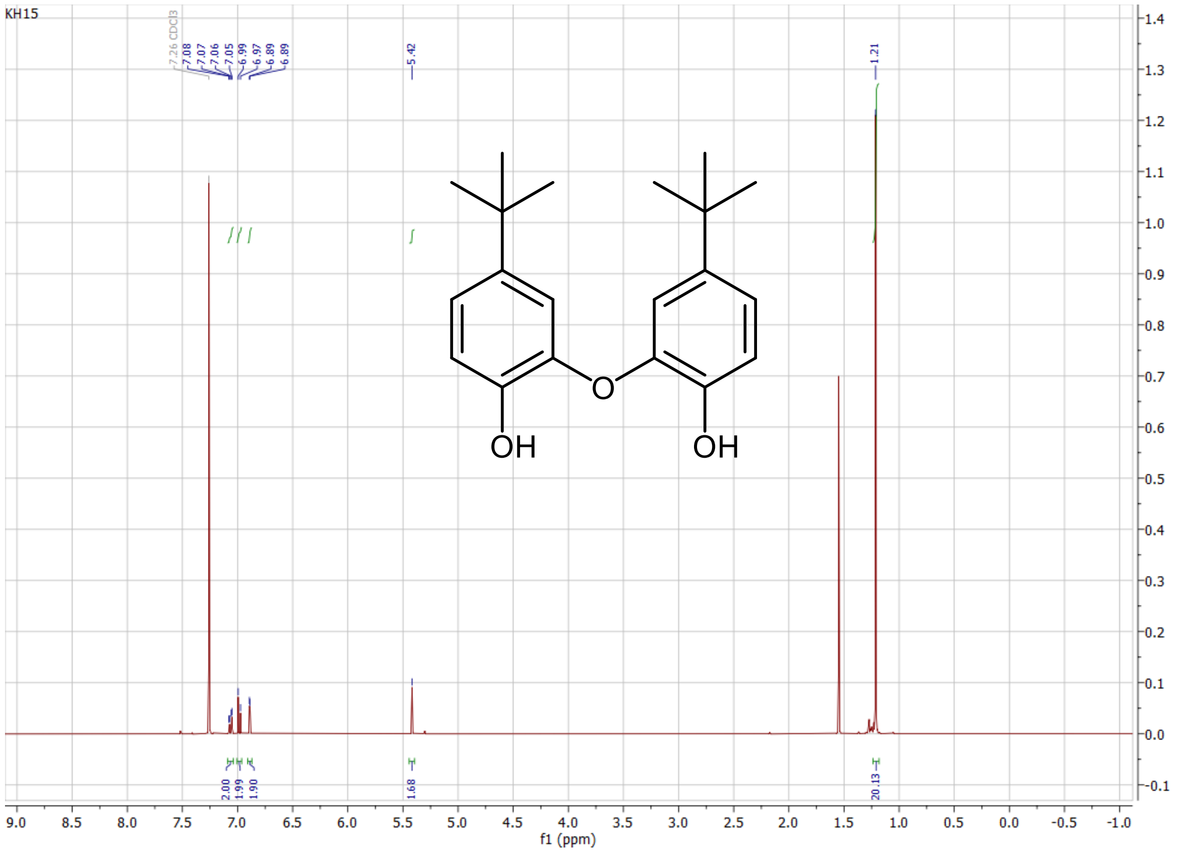

Figure S5: ^{1}H NMR spectrum of compound **8** in $CDCl_3$ (399 MHz)

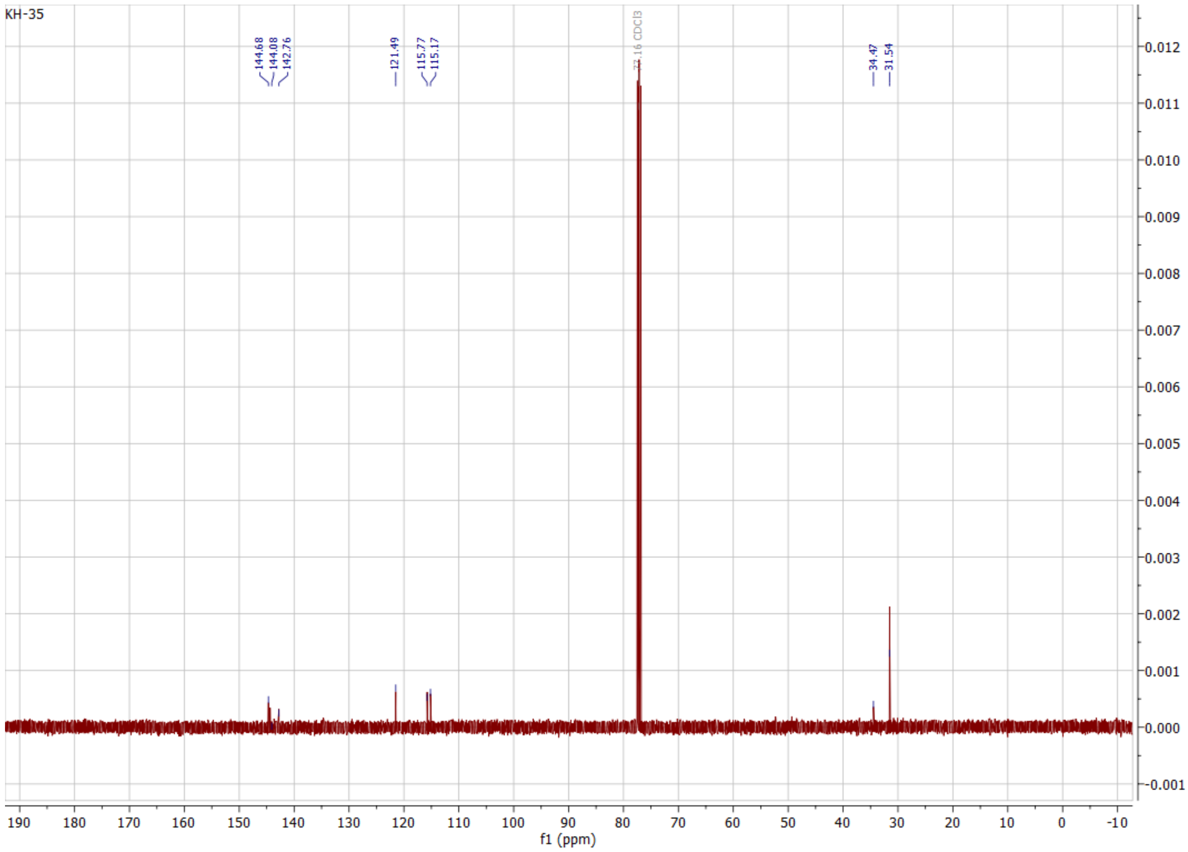

Figure S6: $^{13}C\{^{1}H\}$ NMR spectrum of compound **8** in $CDCl_3$ (101 MHz)

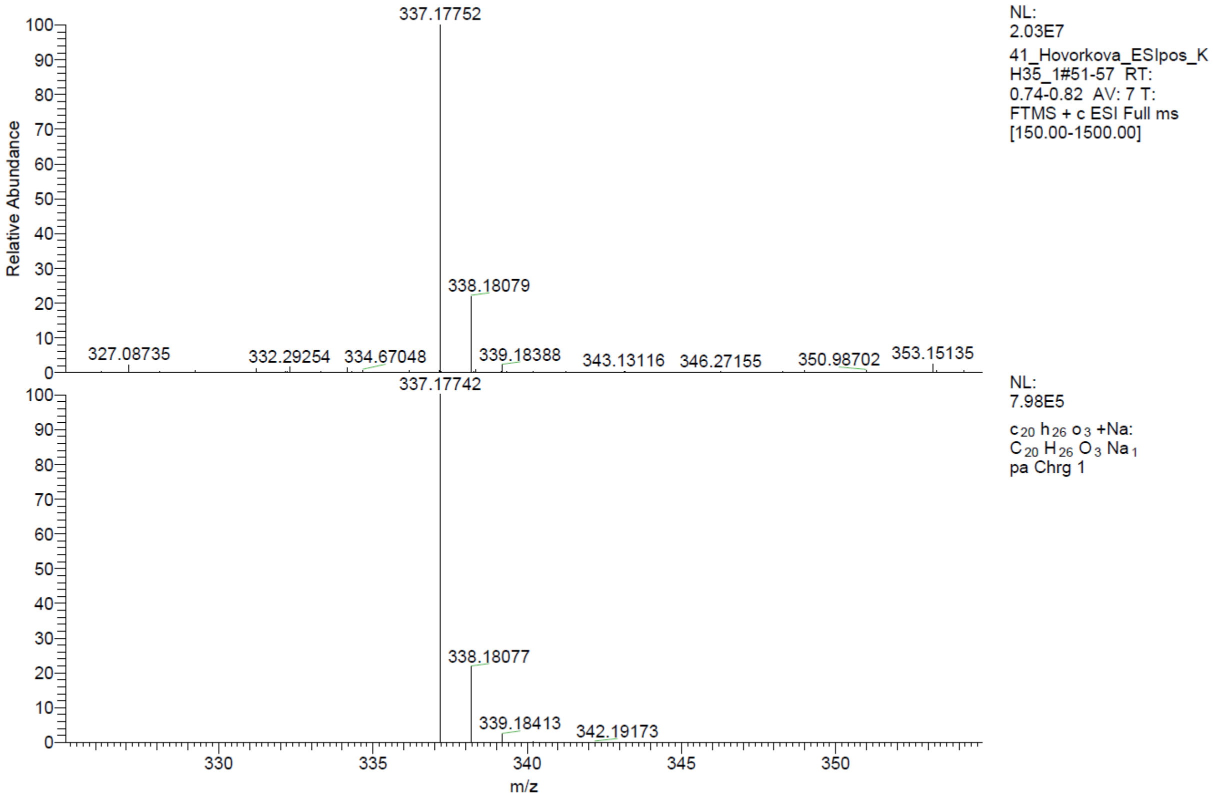

Figure S7: HRMS spectrum of compound **8** in positive mode

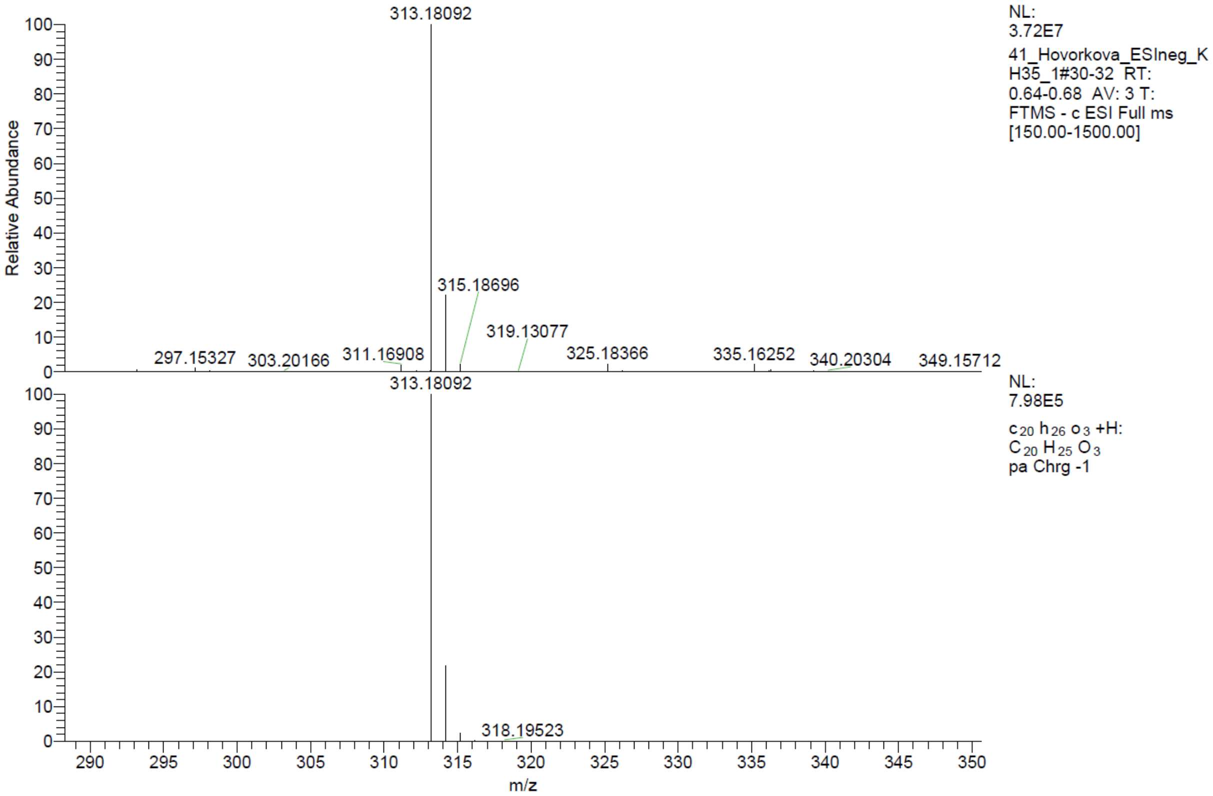

Figure S8: HRMS spectrum of compound **8** in negative mode

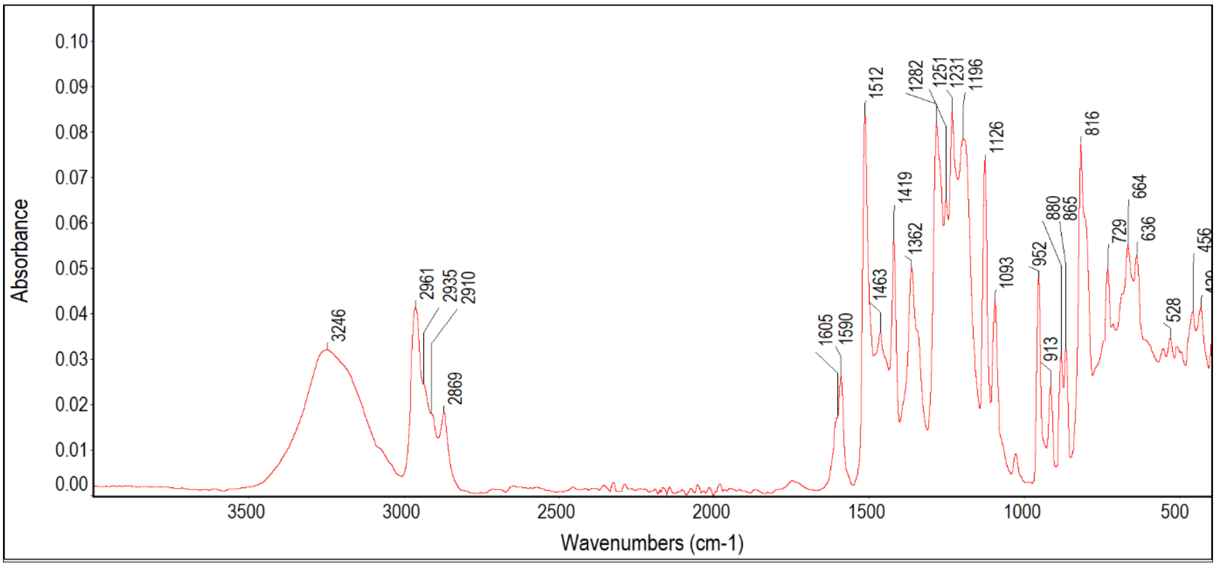

Figure S9: IR spectrum of compound **8**

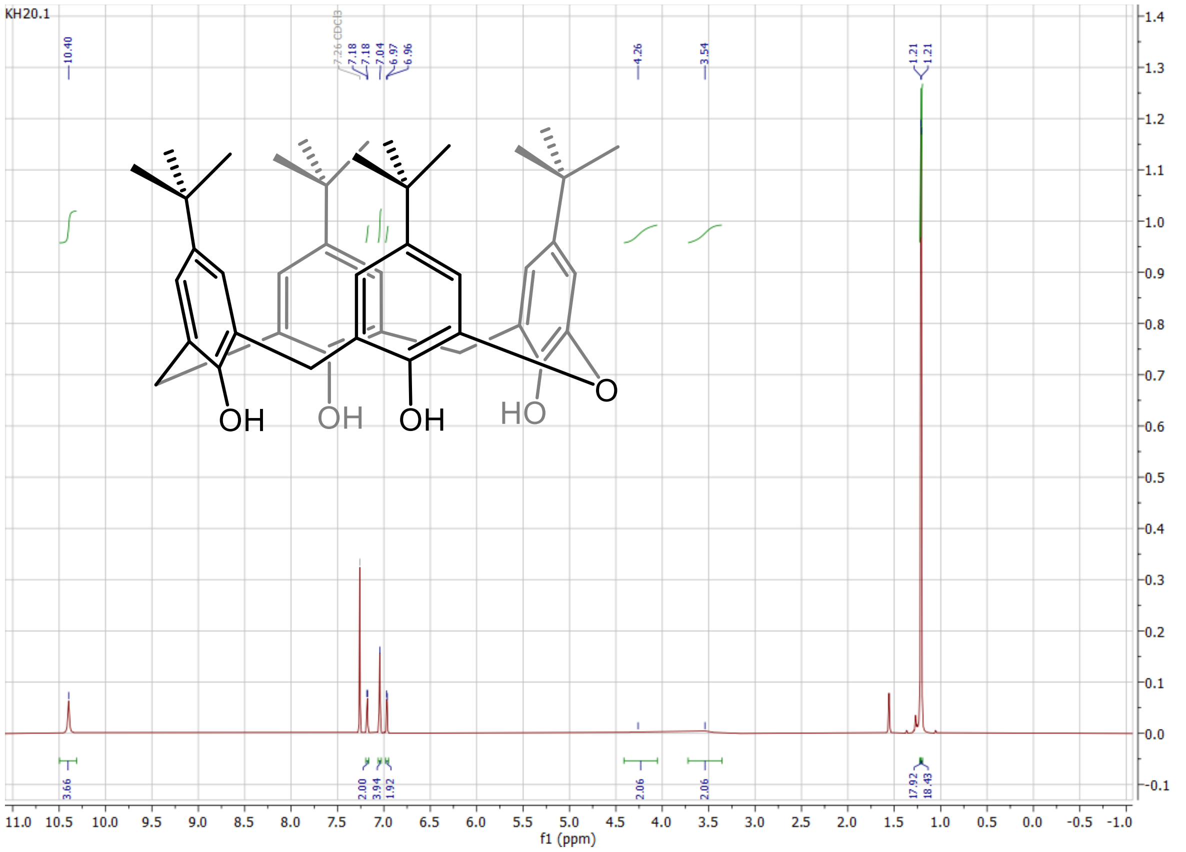

Figure S10: ^{1}H NMR spectrum of compound **10** in $CDCl_3$ at 20 °C (399 MHz)

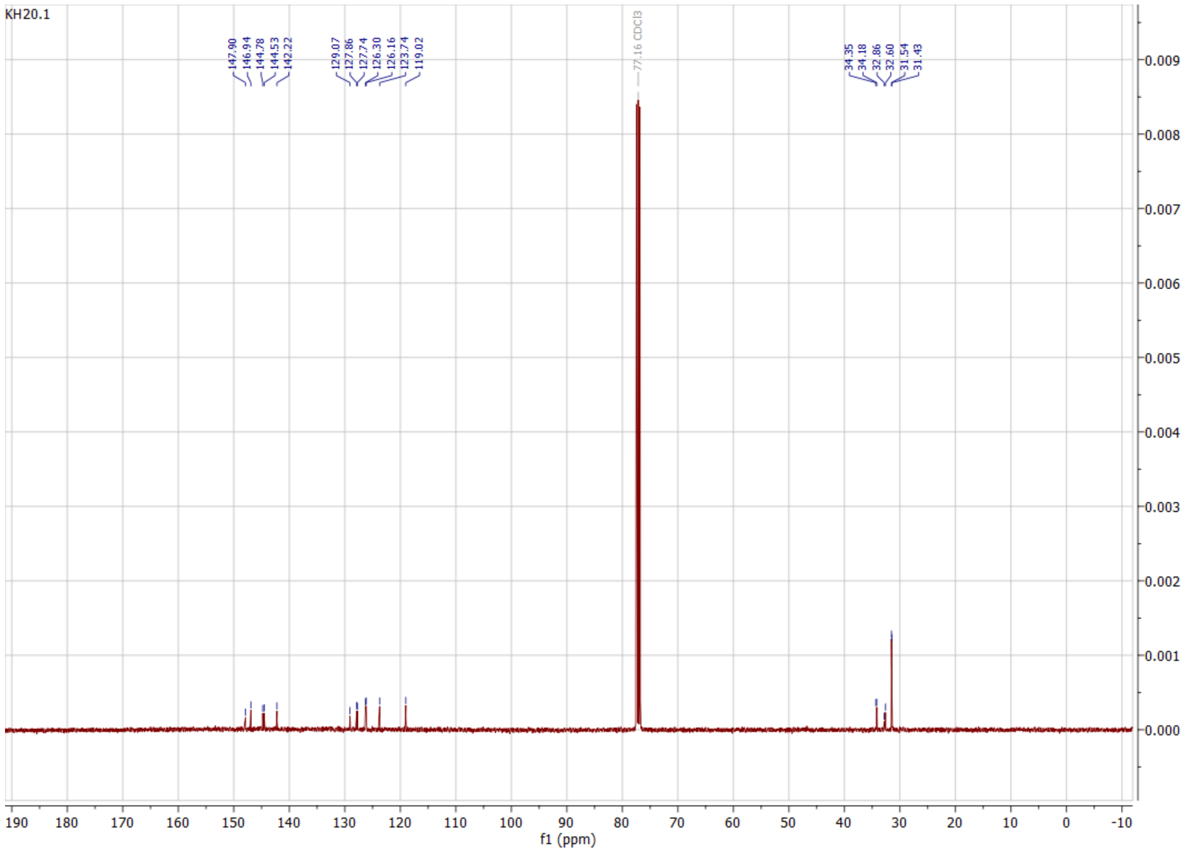

Figure S11: $^{13}C\{^{1}H\}$ NMR spectrum of compound **10** in $CDCl_3$ (101 MHz)

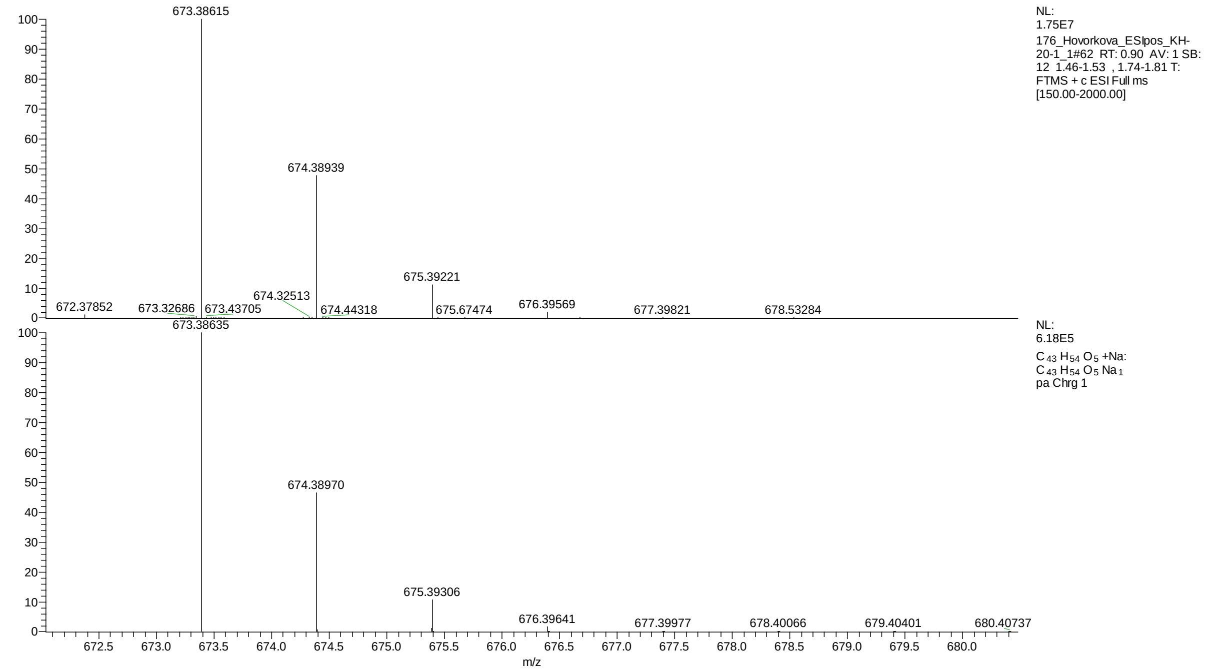

Figure S12: HRMS spectrum of compound **10**

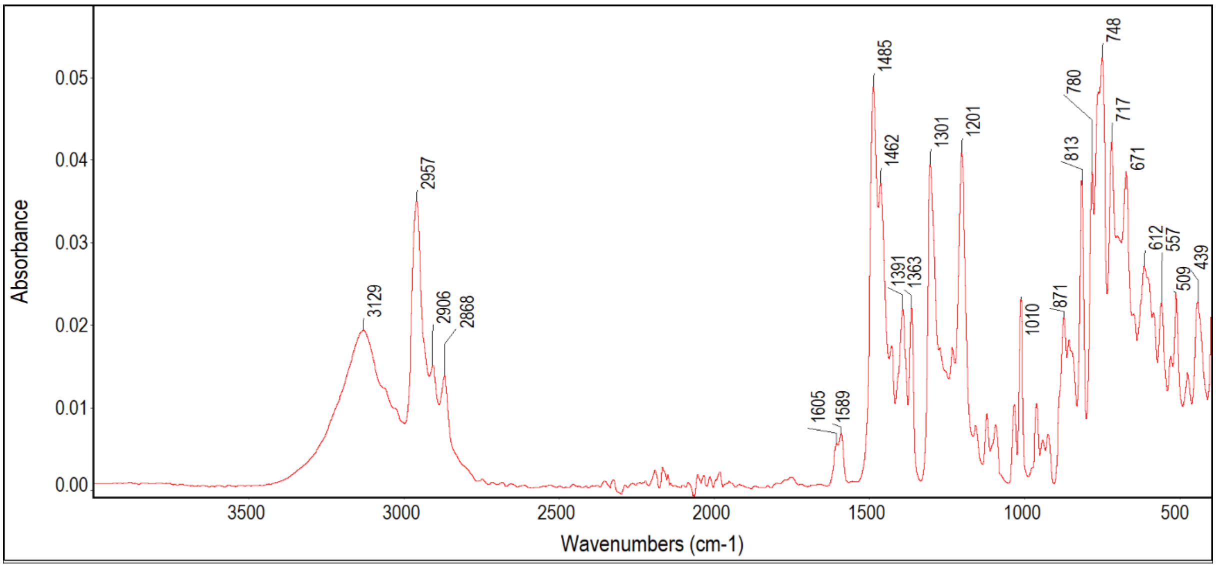

Figure S13: IR spectrum of compound **10**

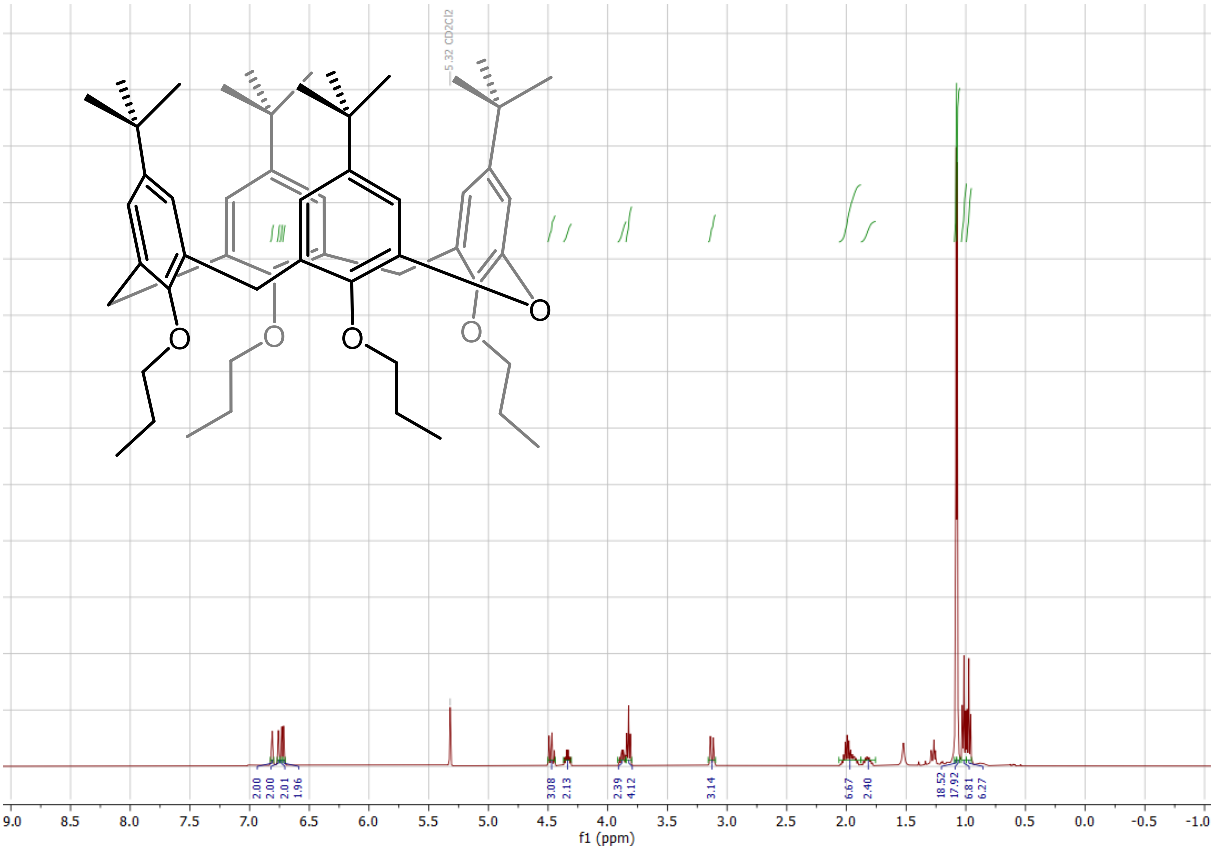

Figure S14: ^{1}H NMR spectrum of compound **11** in CD_2Cl_2 (500 MHz)

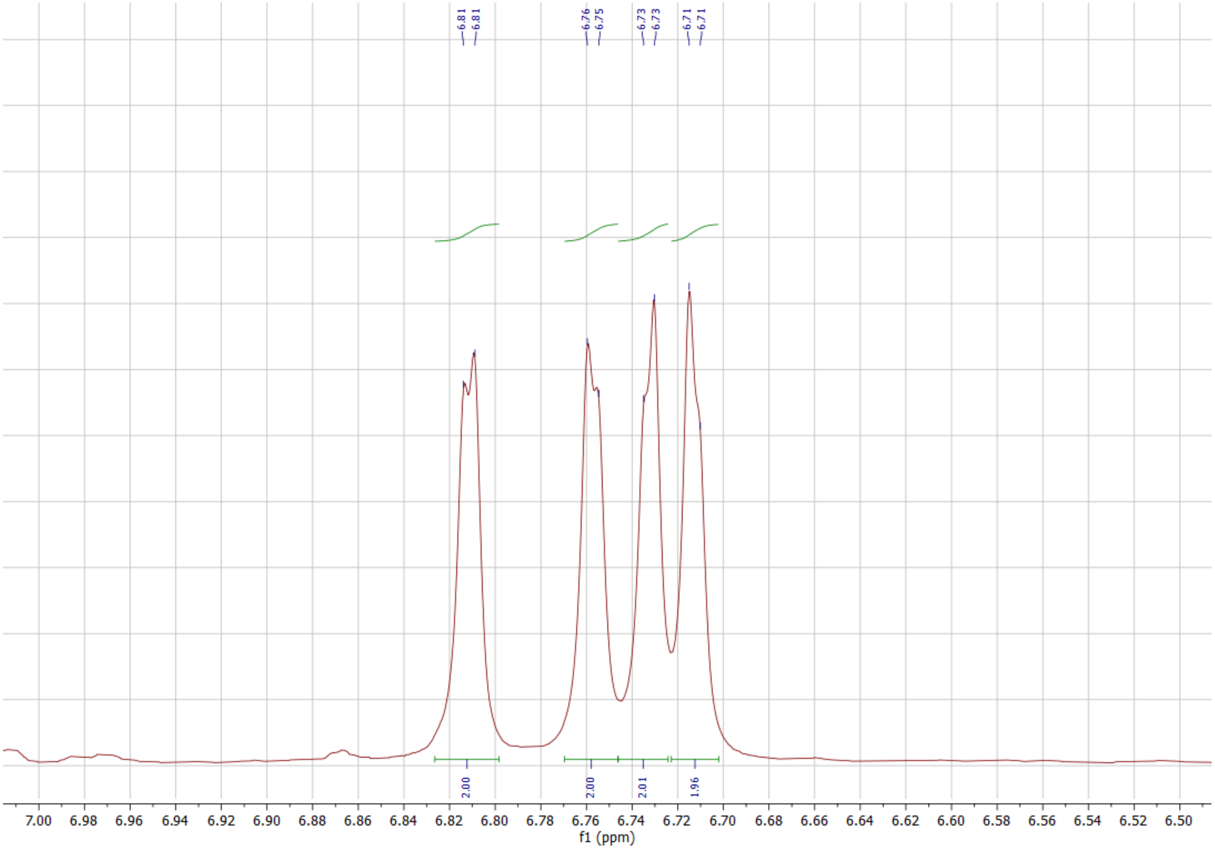

Figure S15: ^{1}H NMR spectrum of compound **11** in CD_2Cl_2 – aromatic section (500 MHz)

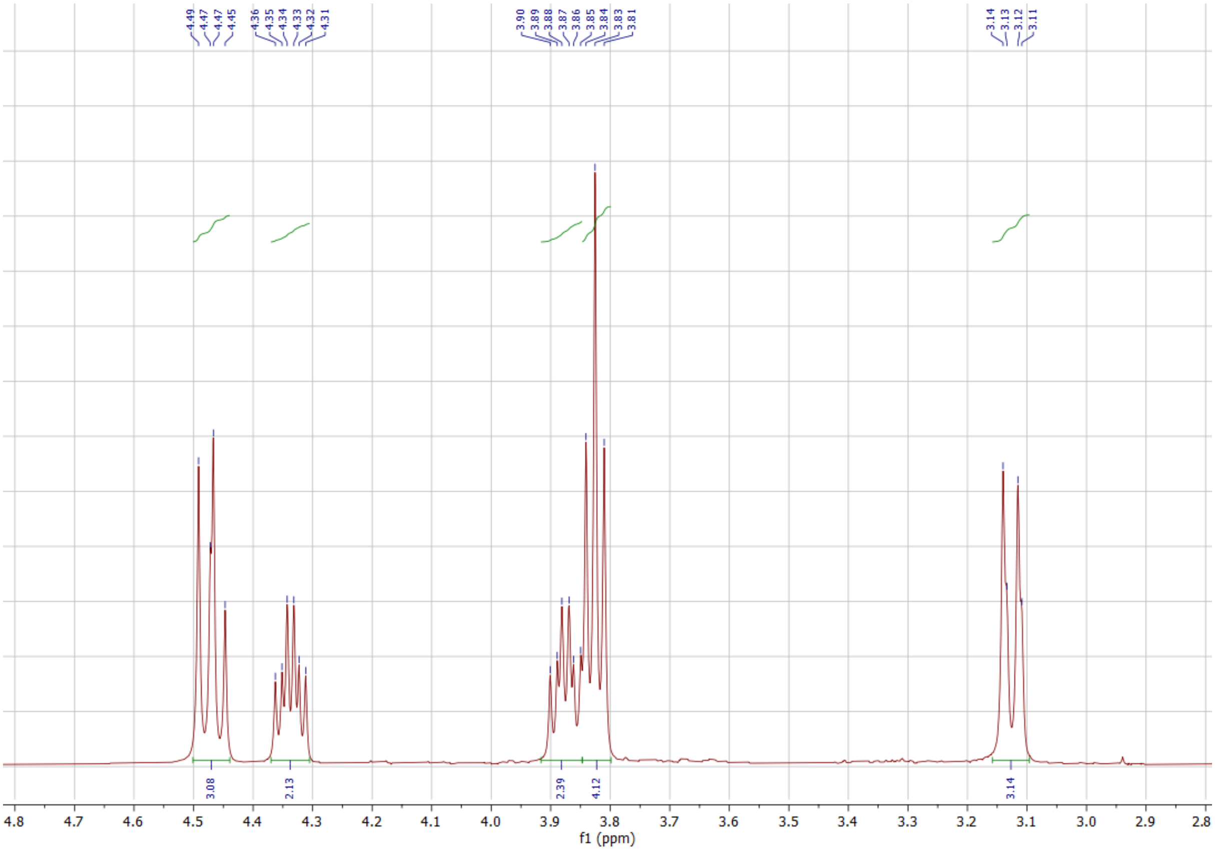

Figure S16: ^{1}H NMR spectrum of compound **11** in CD_2Cl_2 – methylene bridges section (500 MHz)

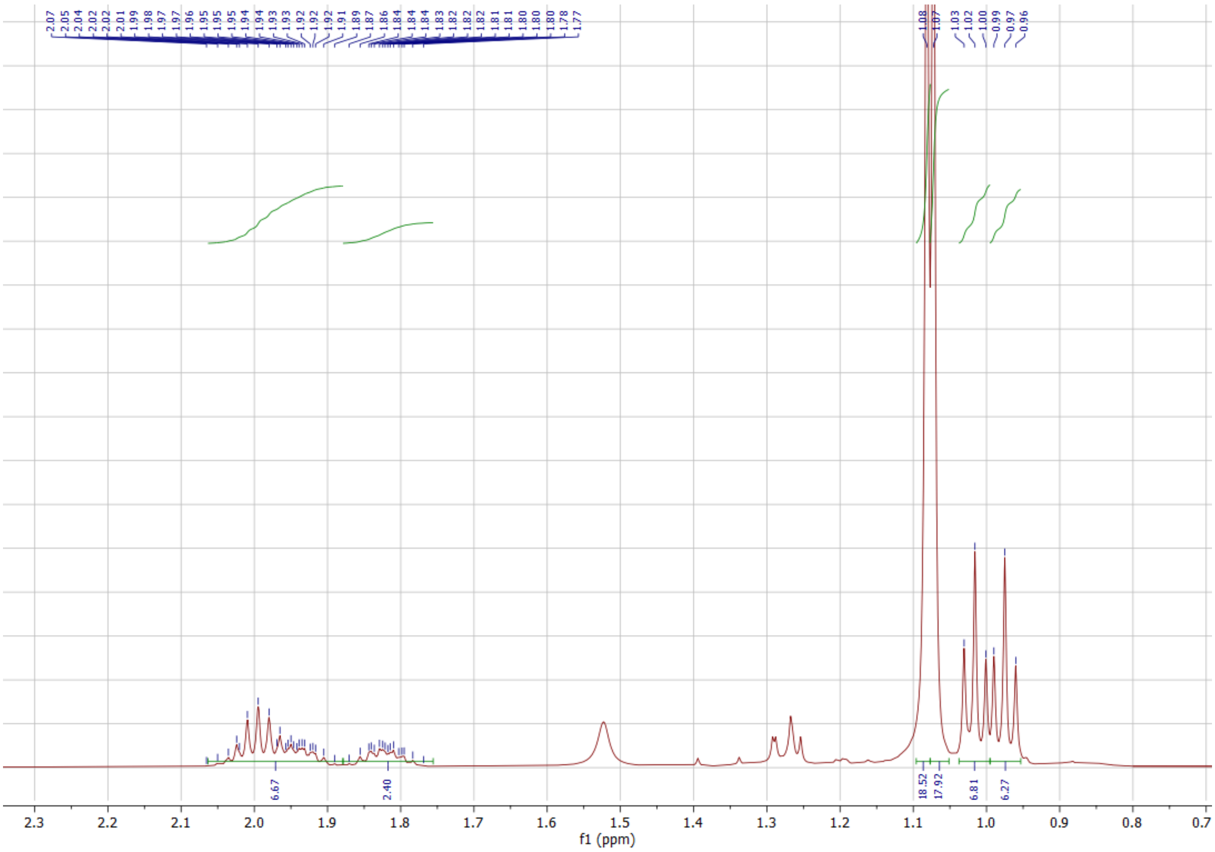

Figure S17: ^{1}H NMR spectrum of compound **11** in CD_2Cl_2 – aliphatic section (500 MHz)

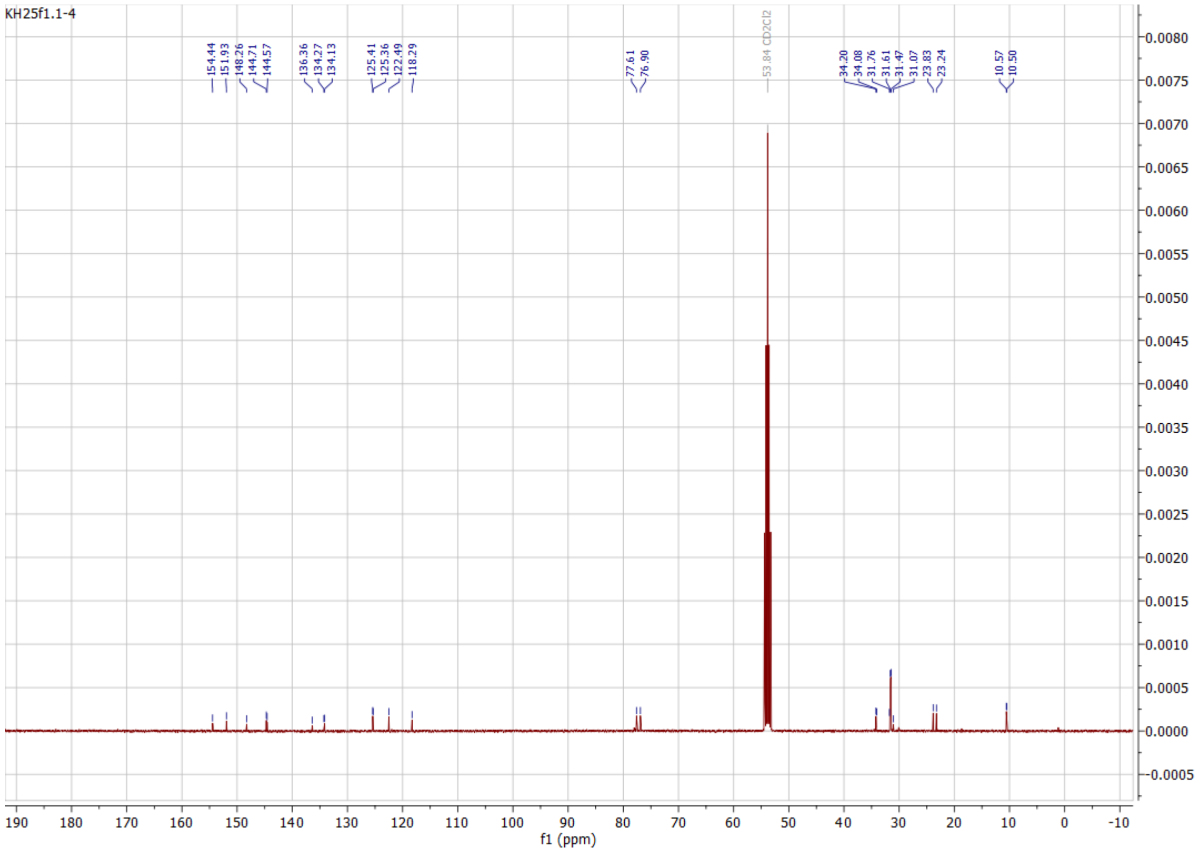

Figure S18: $^{13}C\{^{1}H\}$ NMR spectrum of compound **11** in CD_2Cl_2 (101 MHz)

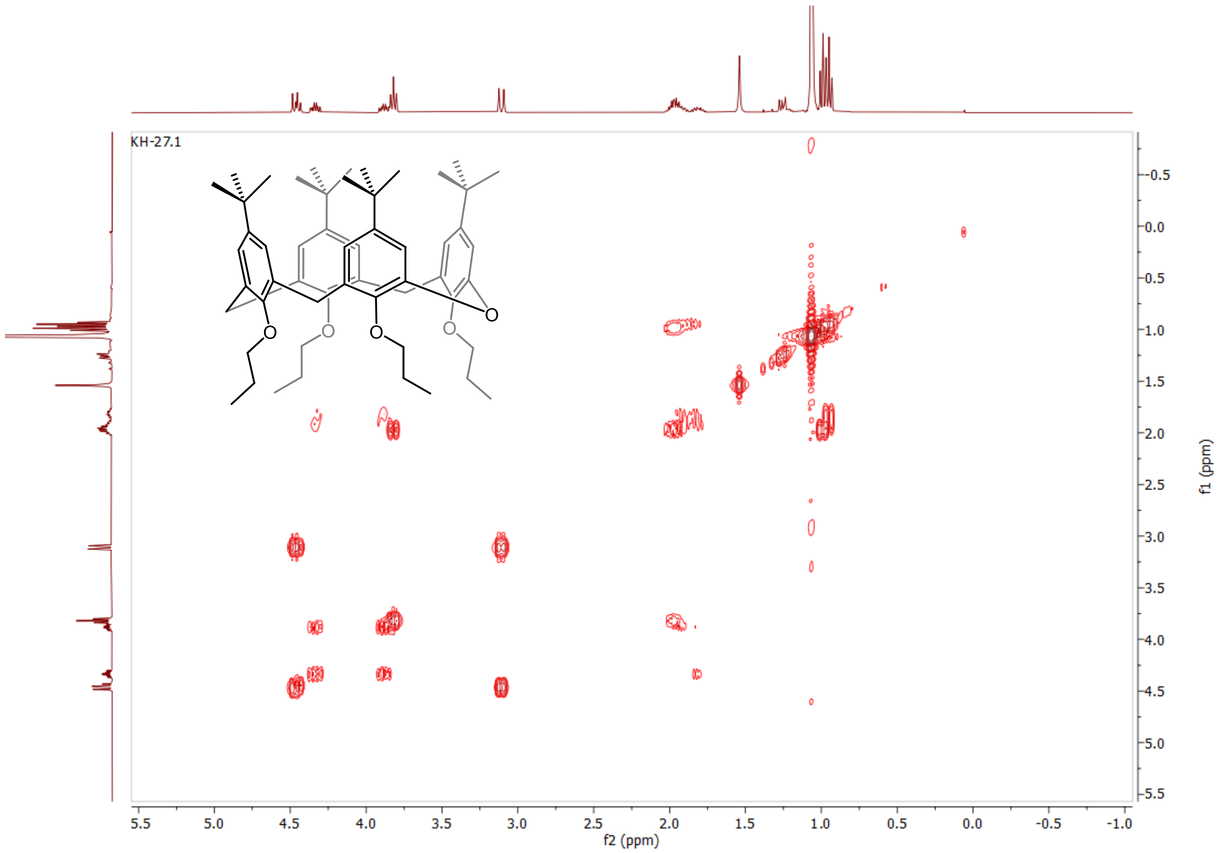

Figure S19: COSY spectrum of compound **11** in $CDCl_3$ (400 MHz)

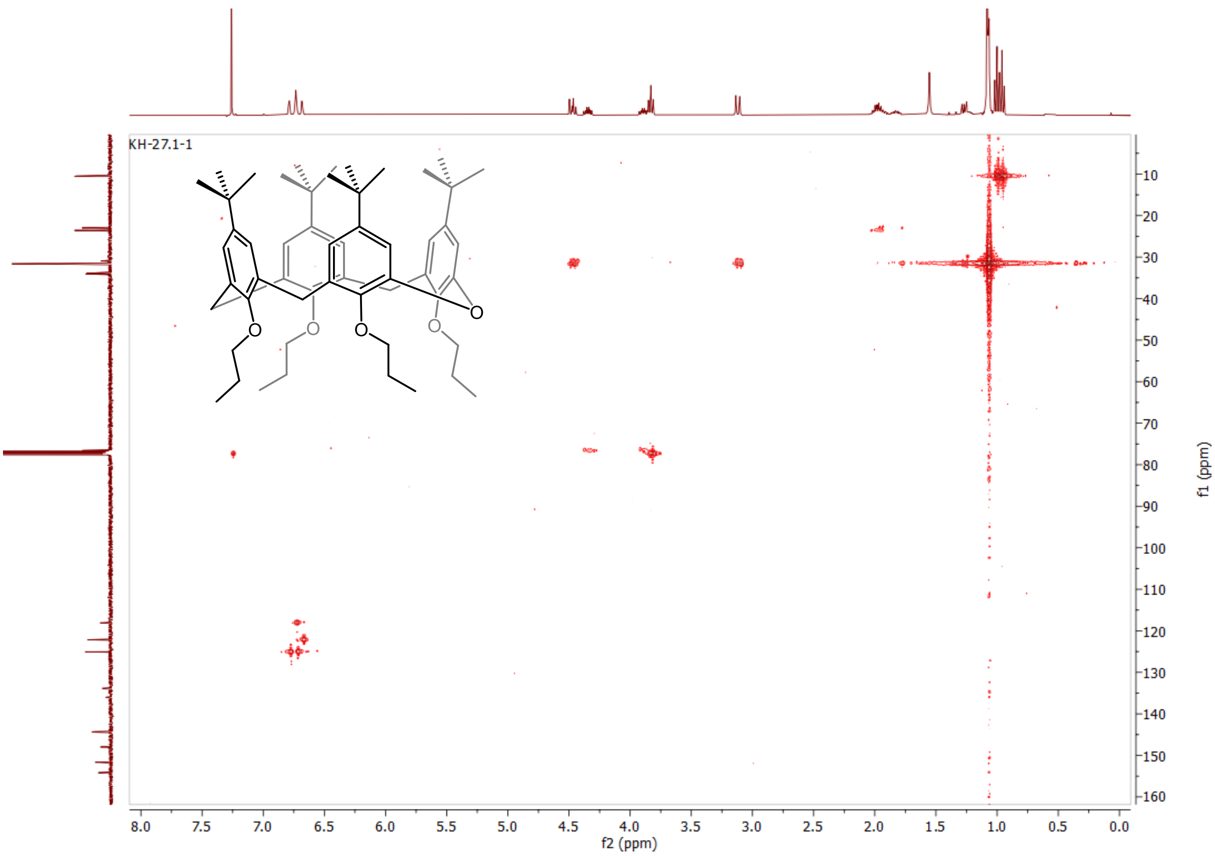

Figure S20: HMQC spectrum of compound **11** in $CDCl_3$ (400 MHz)

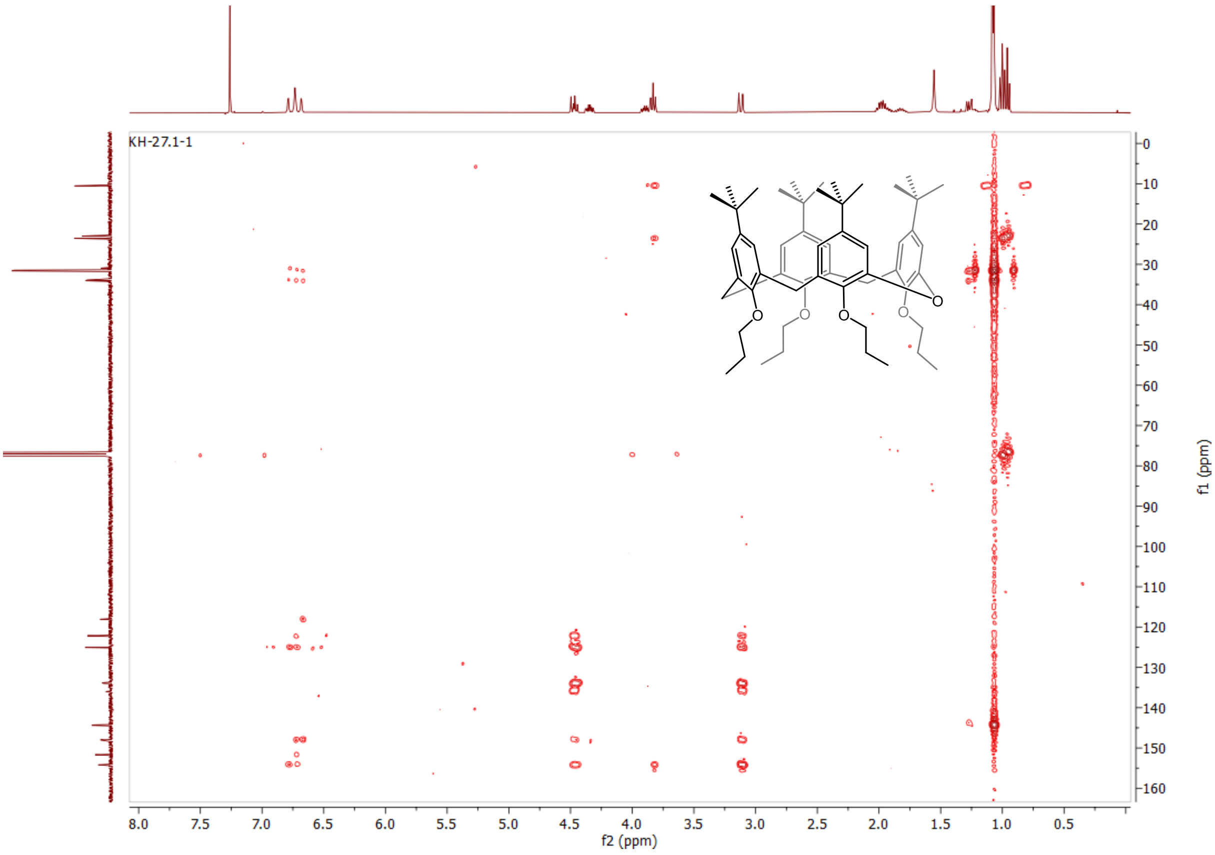

Figure S21: HMBC spectrum of compound **11** in $CDCl_3$ (400 MHz)

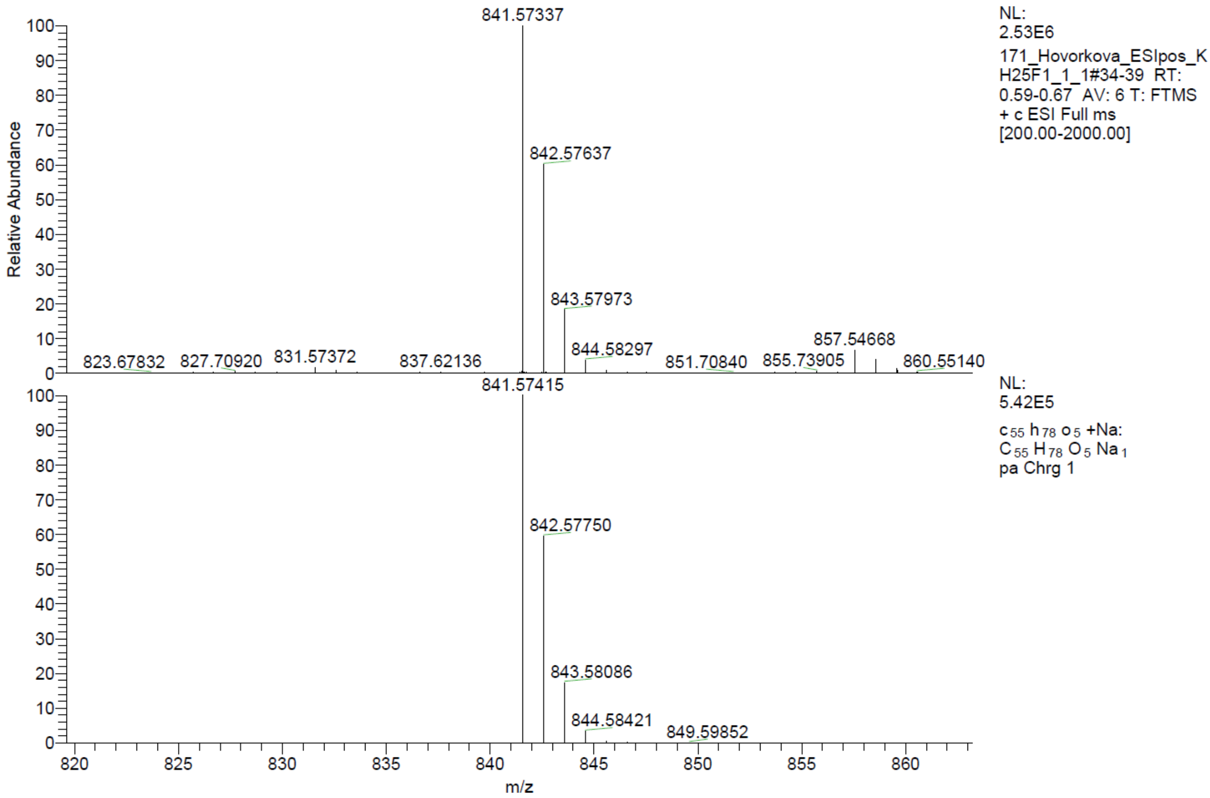

Figure S22: HRMS spectrum of compound **11**

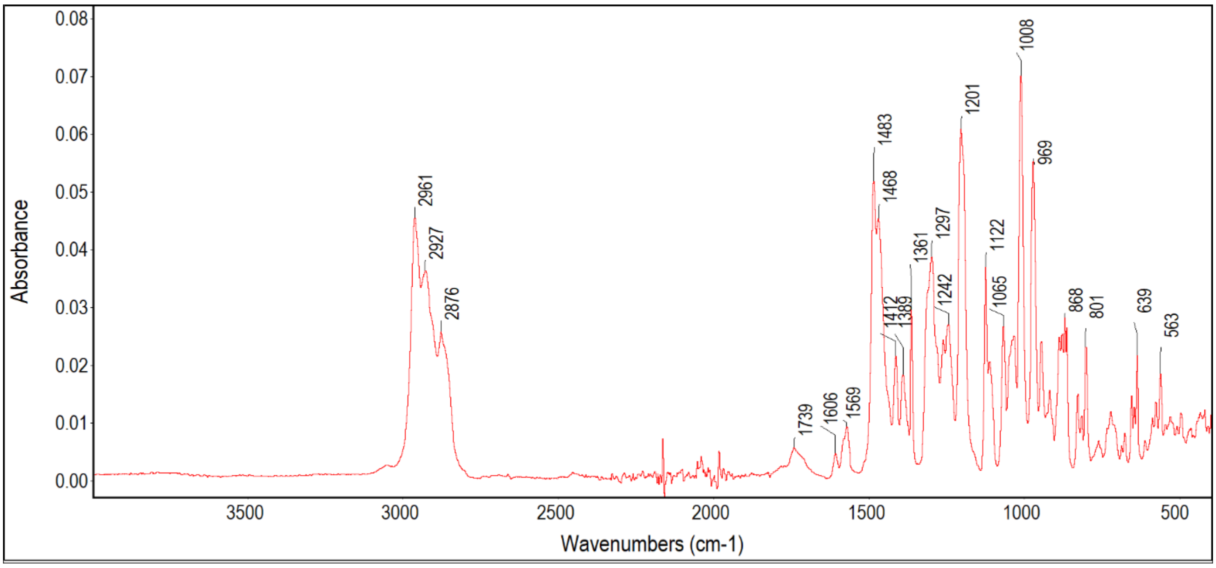

Figure S23: IR spectrum of compound **11**

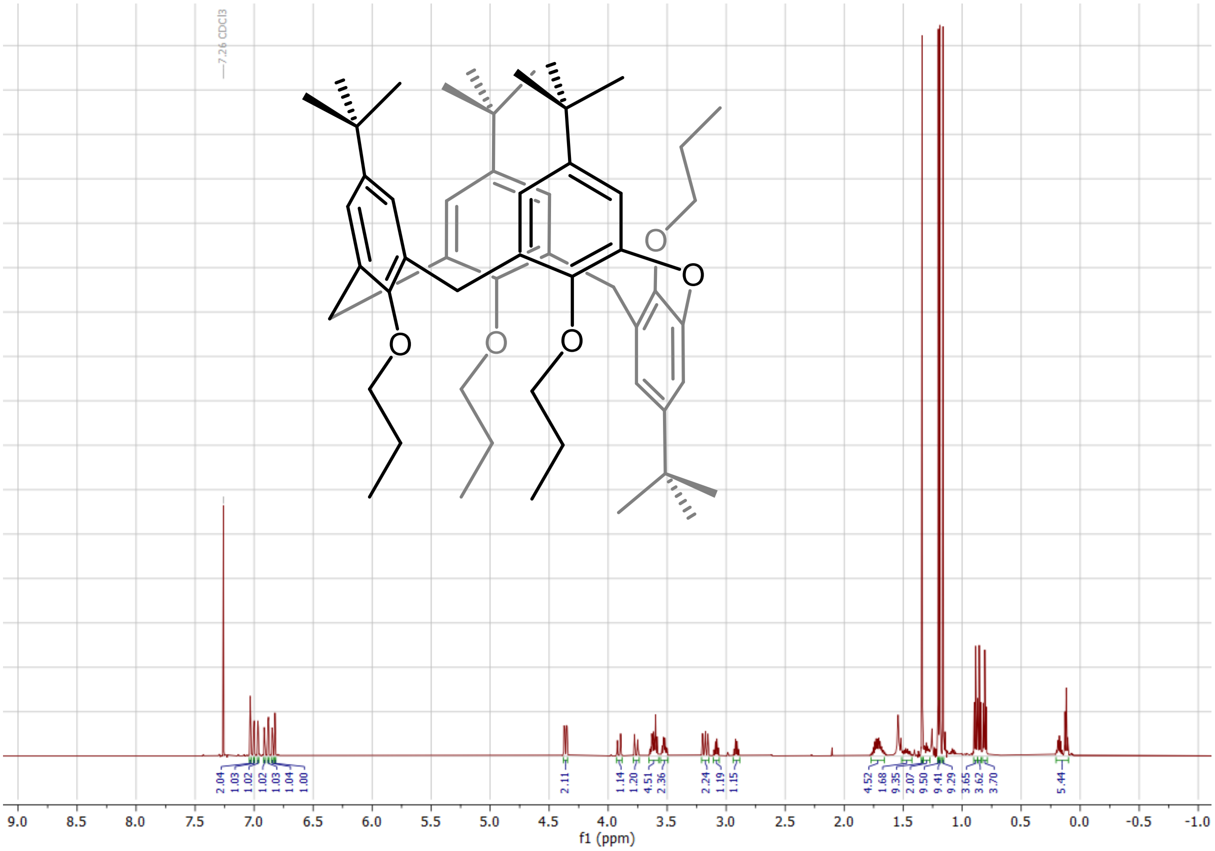

Figure S24: ^{1}H NMR spectrum of compound **12** in $CDCl_3$ (600 MHz)

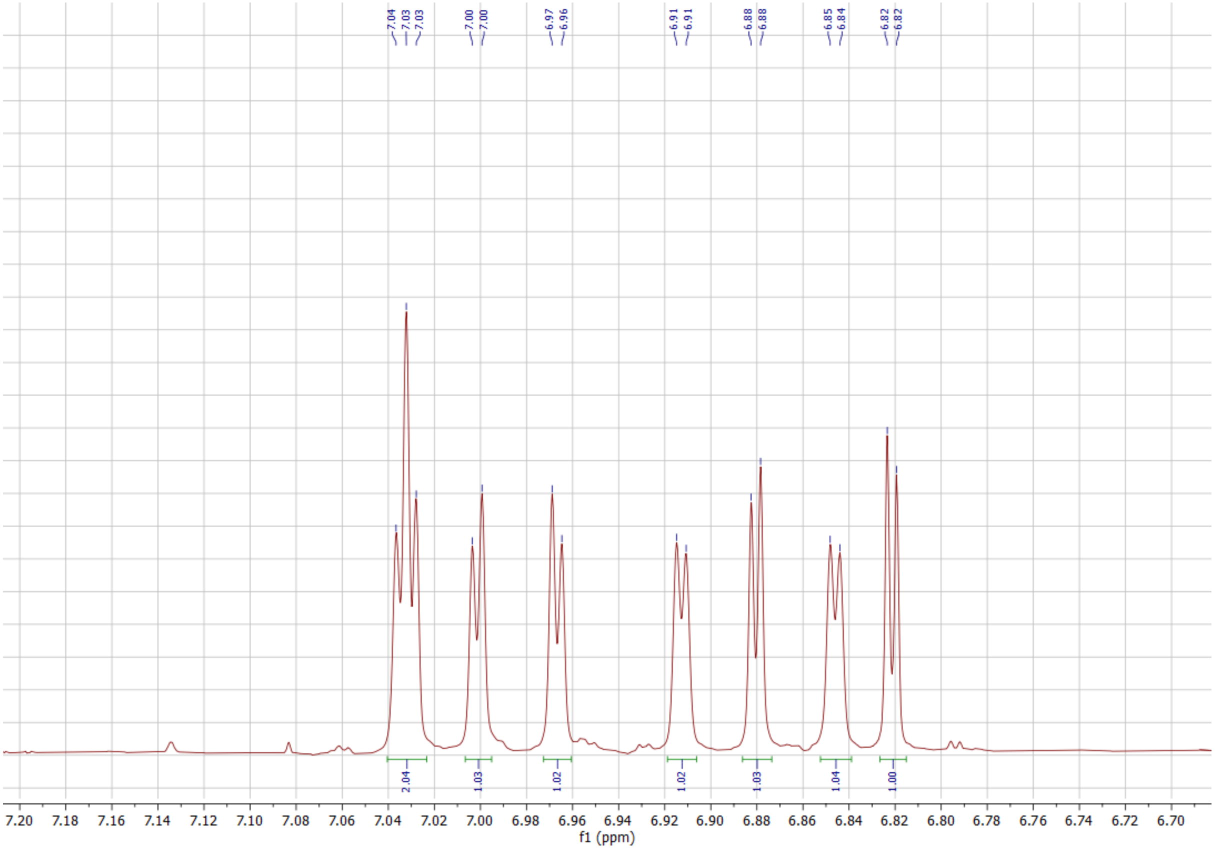

Figure S25: ^{1}H NMR spectrum of compound **12** in $CDCl_3$ – aromatic section (600 MHz)

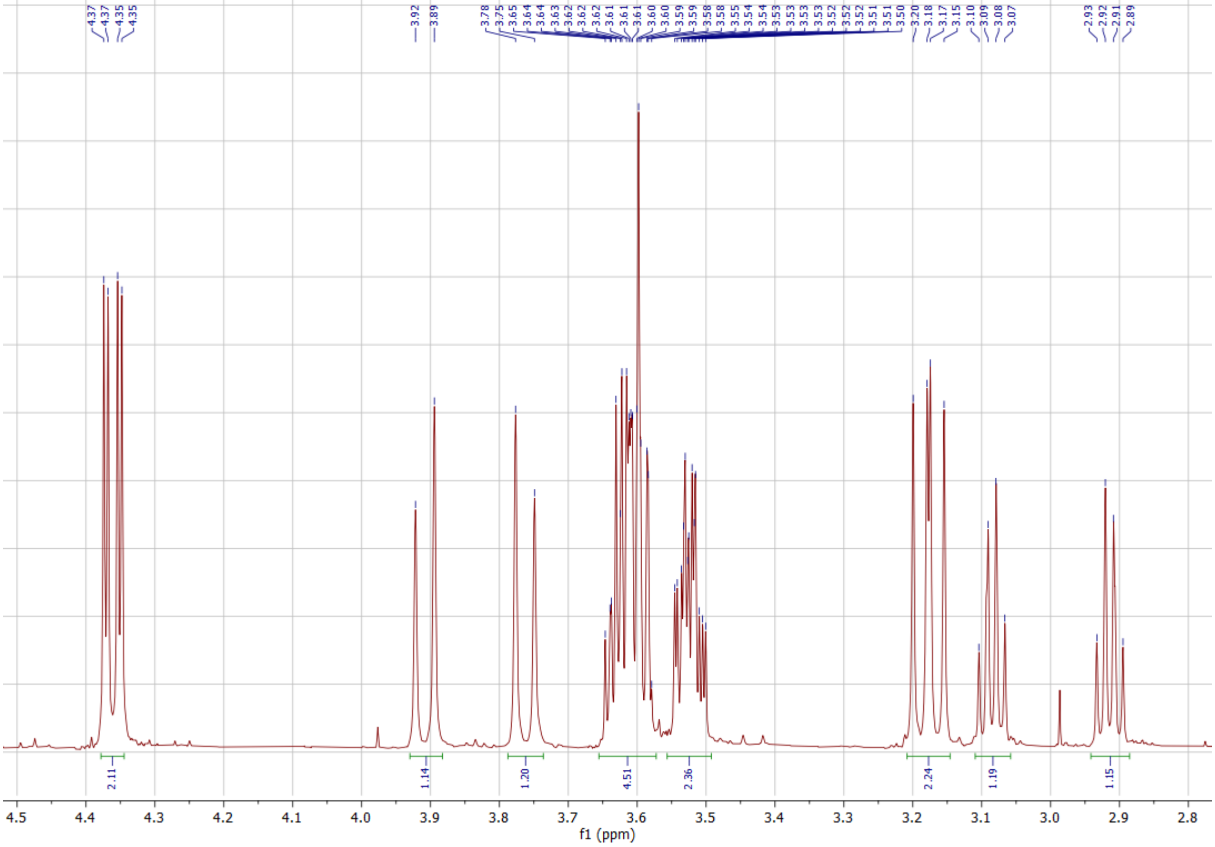

Figure S26: ^{1}H NMR spectrum of compound **12** in $CDCl_3$ – methylene bridges section (600 MHz)

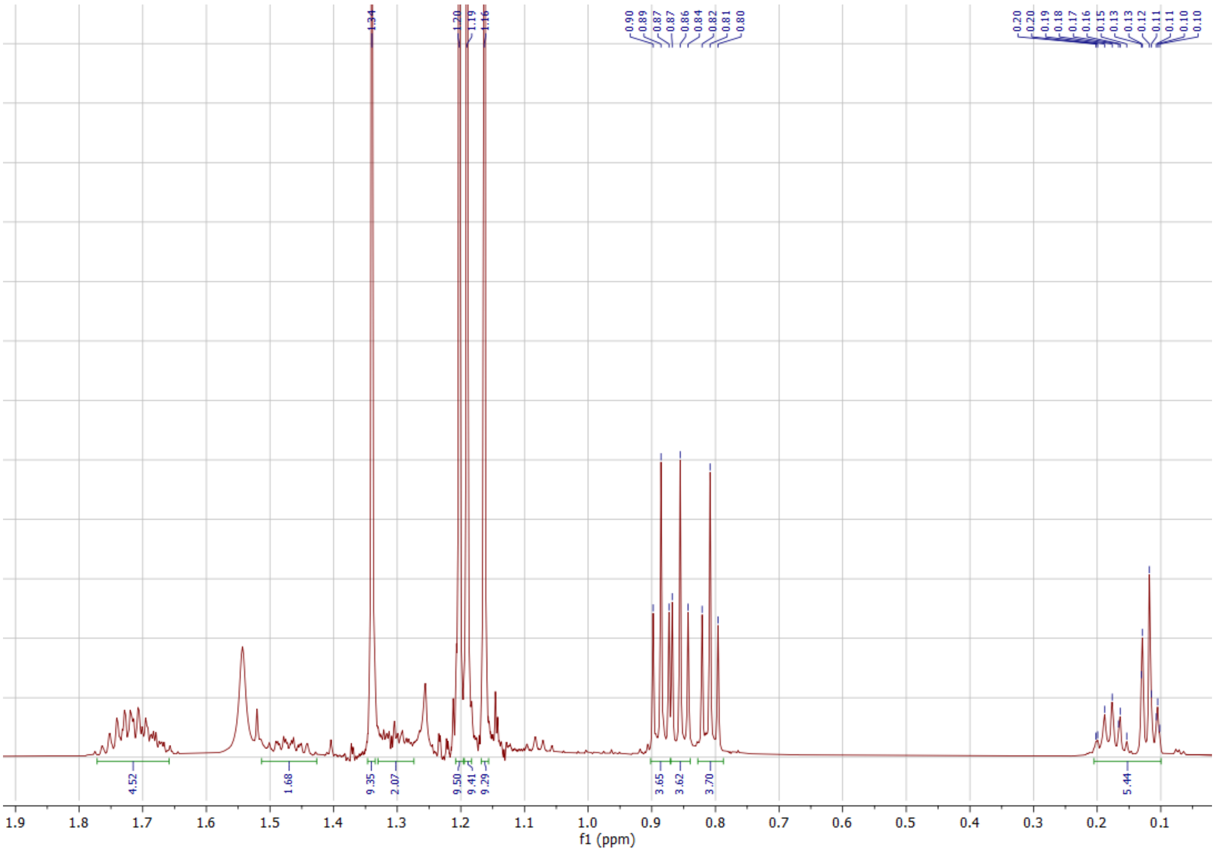

Figure S27: ^{1}H NMR spectrum of compound **12** in $CDCl_3$ – aliphatic section (600 MHz)

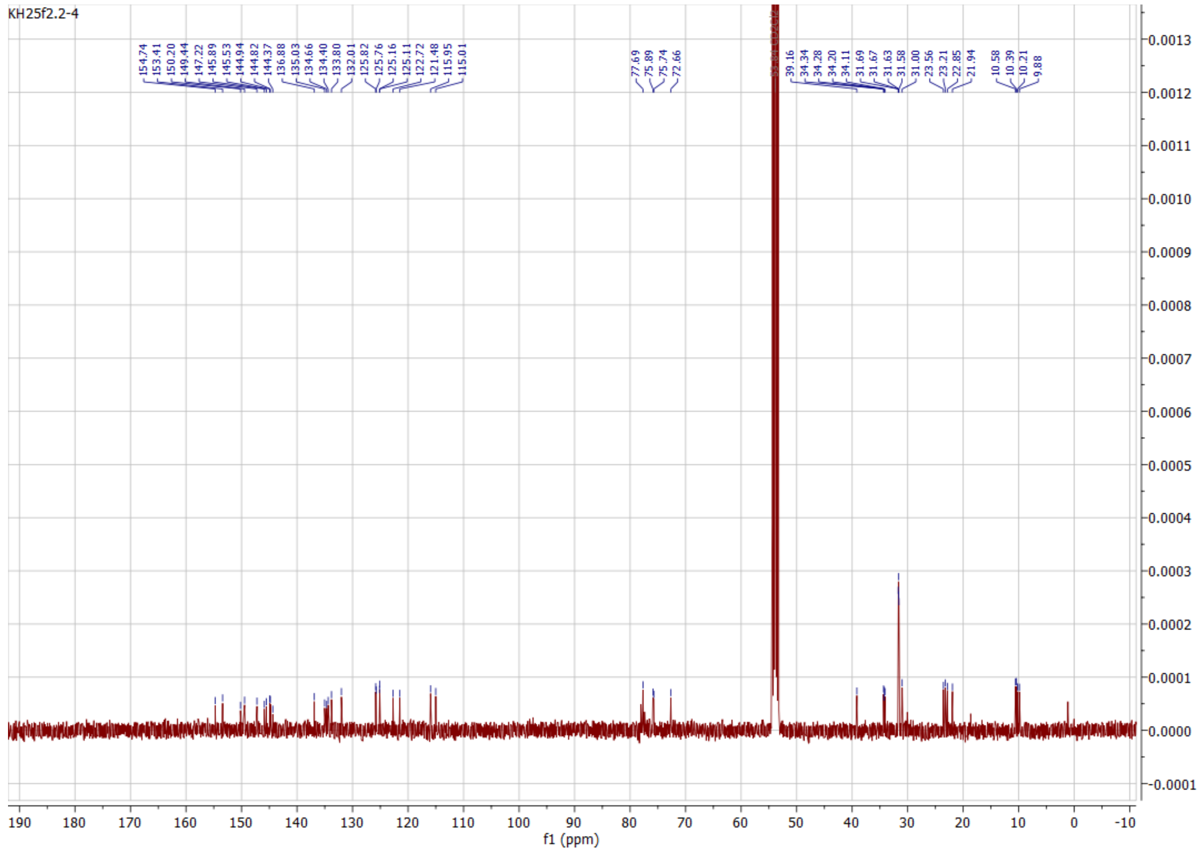

Figure S28: $^{13}C\{^1H\}$ NMR spectrum of compound **12** in CD_2Cl_2 (101 MHz)

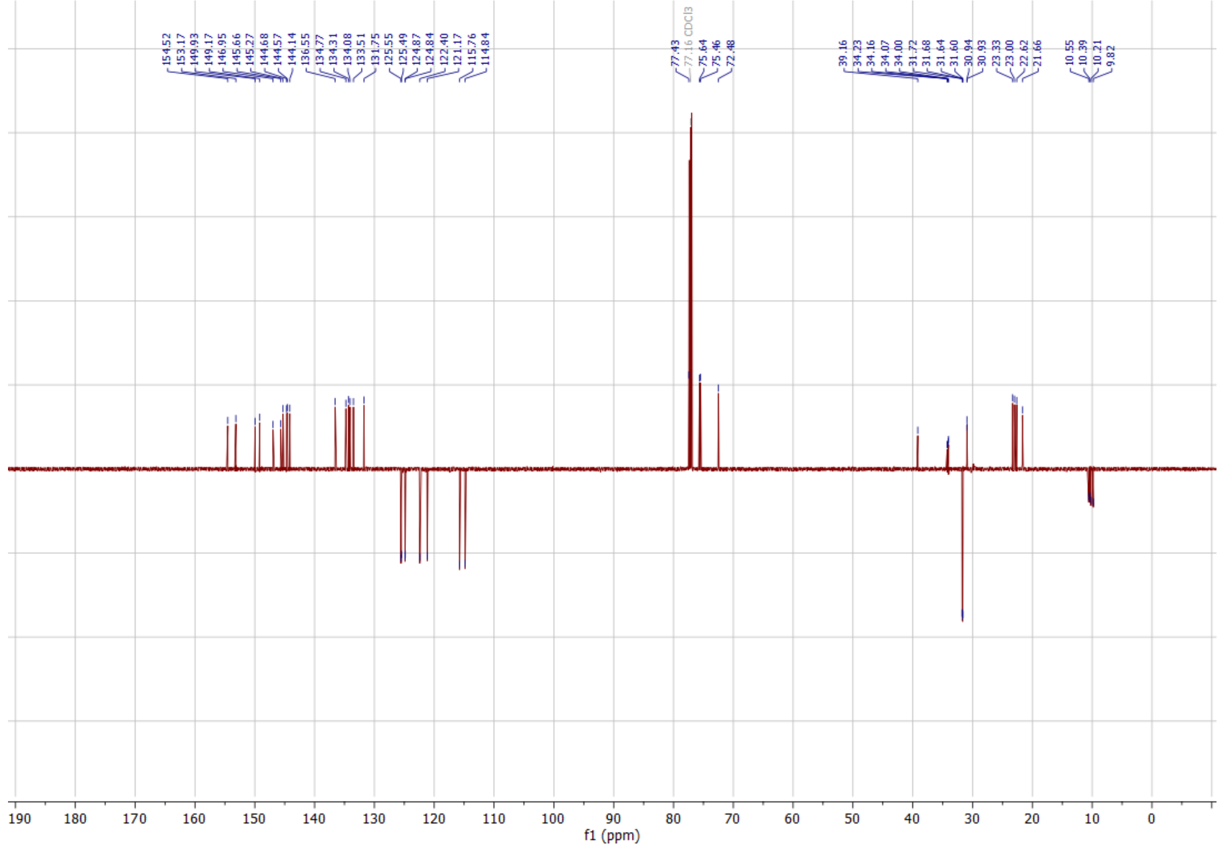

Figure S29: APT NMR spectrum of compound **12** in $CDCl_3$ (151 MHz)

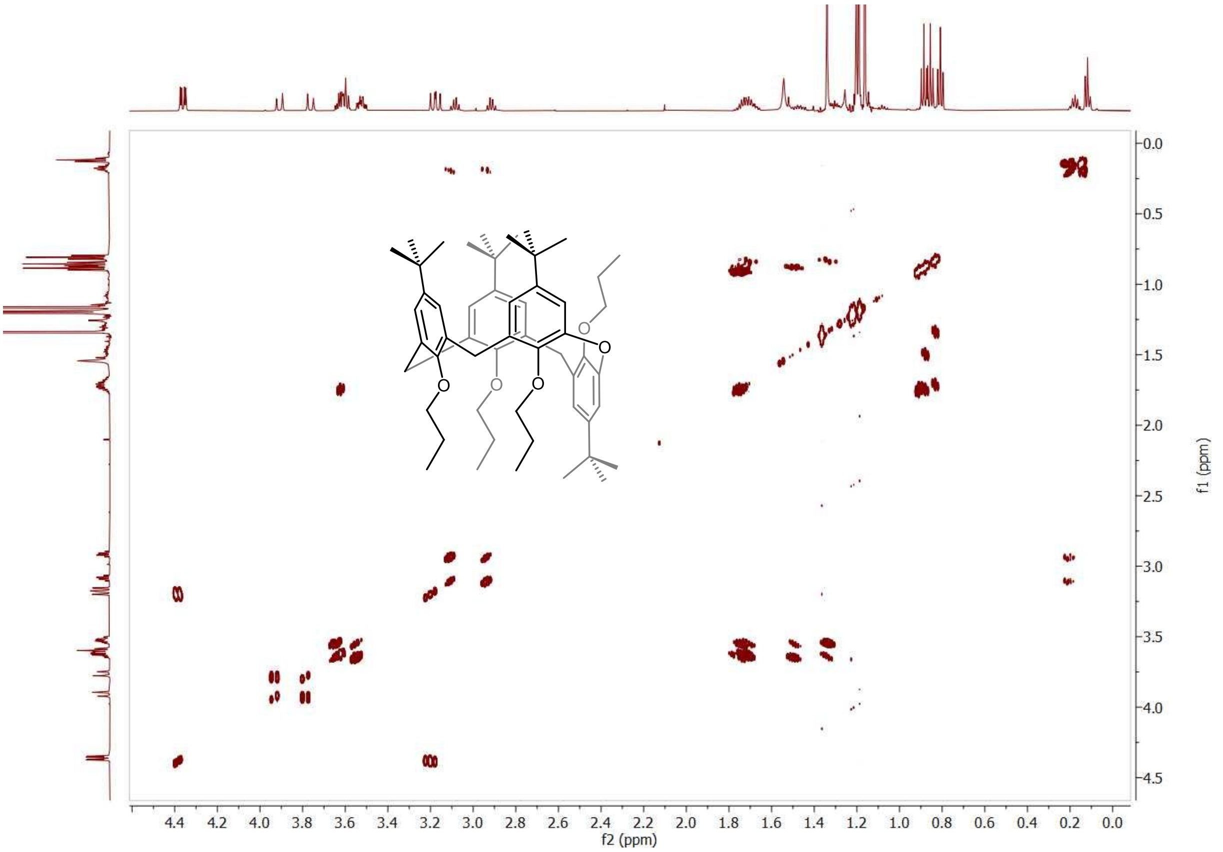

Figure S30: COSY spectrum of compound **12** in CD_2Cl_2 (600 MHz)

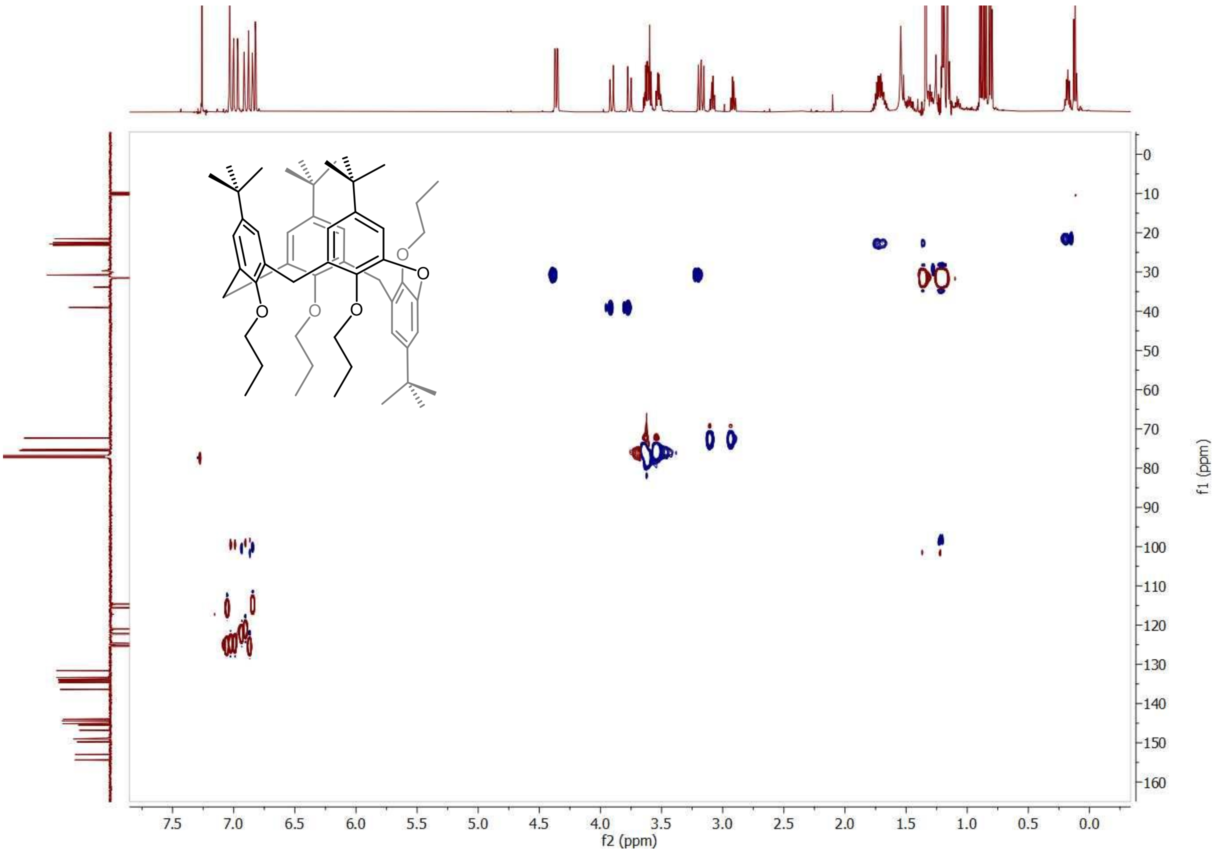

Figure S31 HSQC spectrum of compound **12** in CD_2Cl_2 (600 MHz)

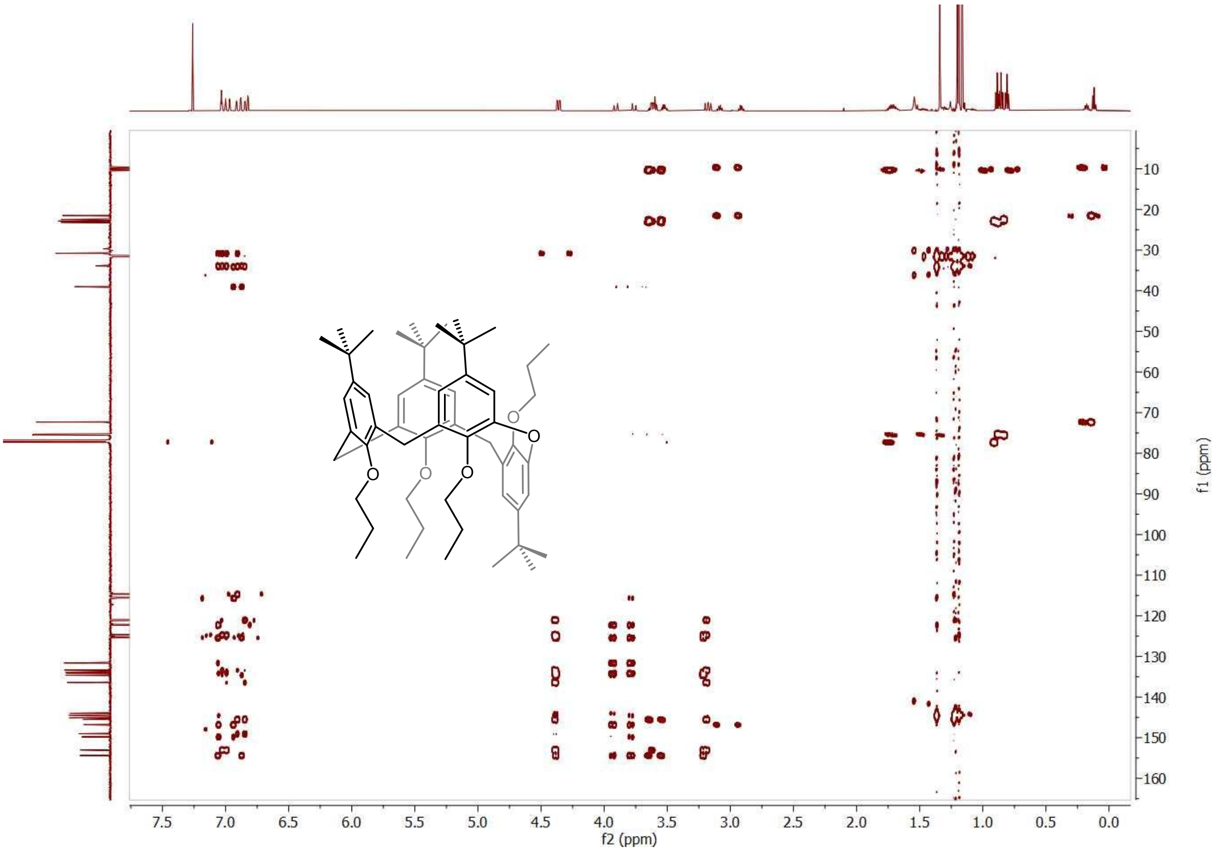

Figure S32: HMBC spectrum of compound **12** in CD_2Cl_2 (600 MHz)

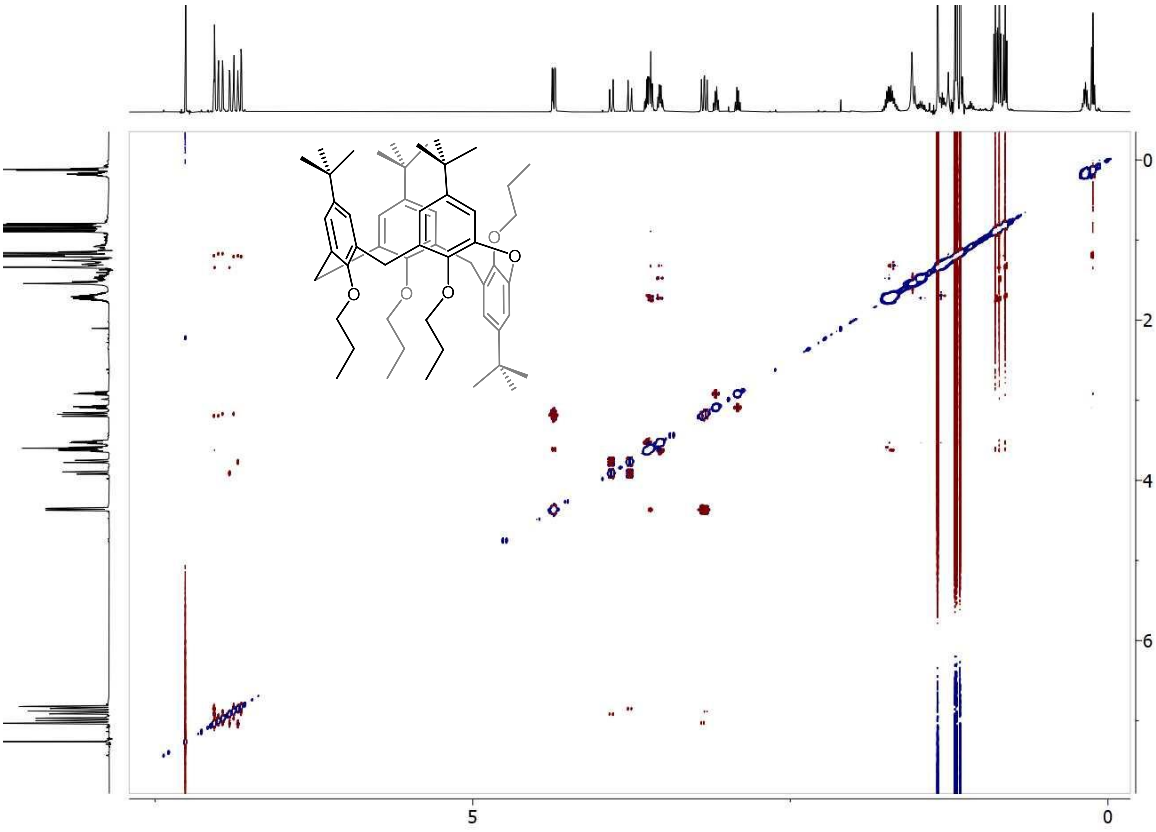

Figure S33: NOESY spectrum of compound **12** in CD_2Cl_2 (600 MHz)

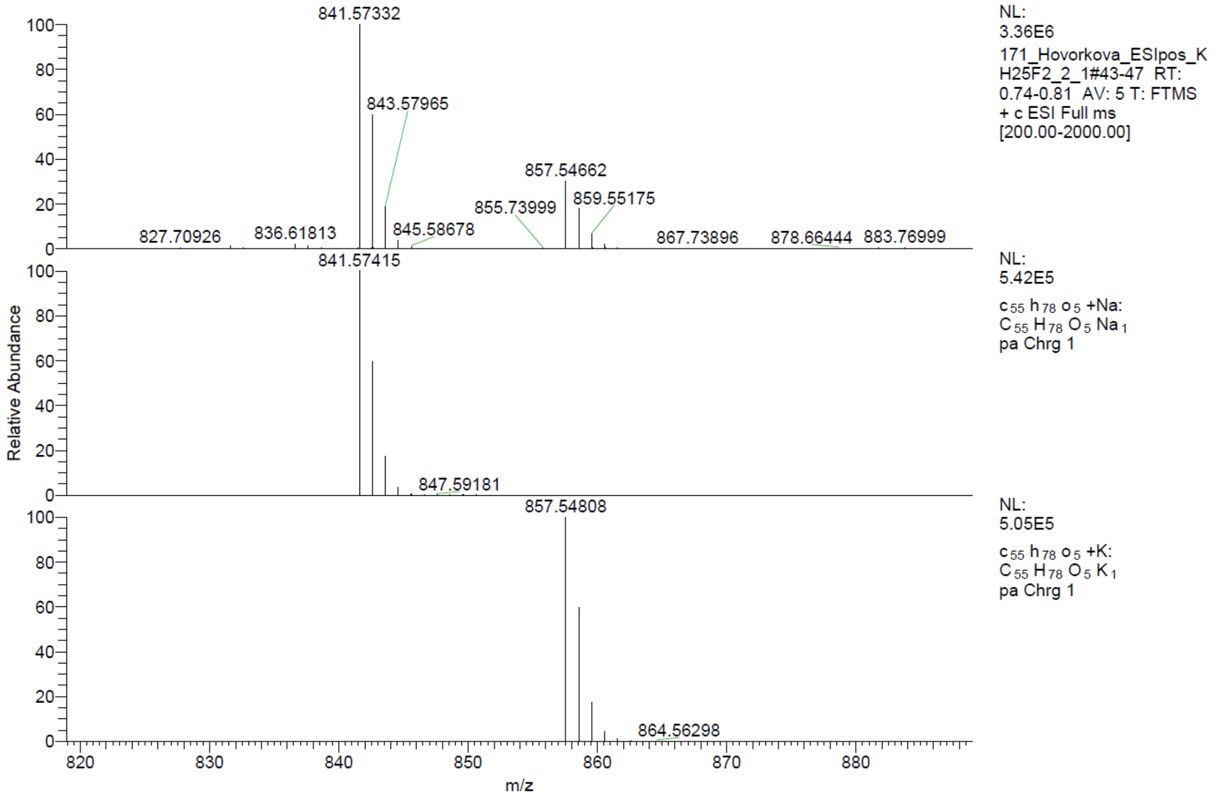

Figure S34: HRMS spectrum of compound **12**

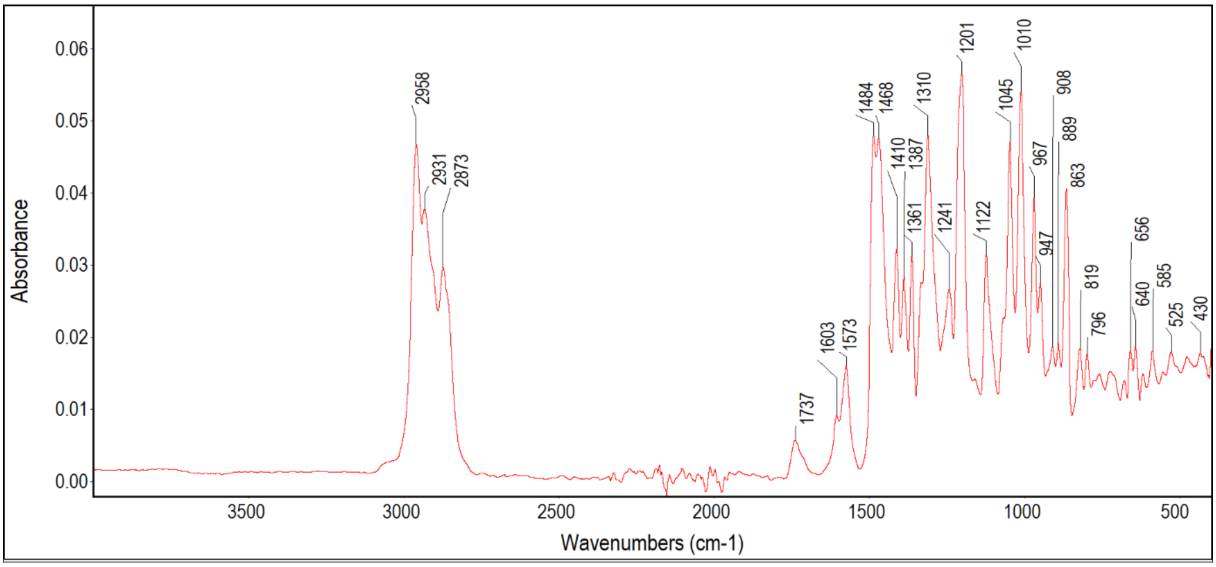

Figure S35: IR spectrum of compound **12**

4. Variable temperature NMR spectra

Variable temperature studies of **10** were performed on Bruker Avance III 500 (^{1}H: 500.13 MHz, Bremen, Germany). The dynamic behaviour of methylene bridges was followed in the temperature range 267 – 333 K in $C_2D_2Cl_4$ (δ = 6.00 ppm). At the lowest temperature, the system provided signals of three methylene bridges in two separate sets for axial and equatorial hydrogen atoms, each of them containing signals in the ratio 2:1 due to the symmetry of the molecule. At higher temperature, the signals of corresponding axial and equatorial protons collapsed in two lines preserving the original ratio 2:1. A similar behaviour was observed also in CD_2Cl_2 (δ = 5.30 ppm). The obtained spectra in the range 263 – 298 K were analysed in Dynamic NMR Models (dNMR) in the TopSpin (version 4.5.0) software in order to obtain the rate constant k for each temperature and calculate thermodynamic parameters. The chemical exchange of 2 symmetrical CH_2 groups and the residual one were fitted independently providing two rate constants k_1 and k_2. Two restrains were applied, the intensity of fitted systems were kept in the approximate ratio 2:1 due to the intensity ratio of both systems and $k_1 \cong k_2$ as both describe the same dynamic process and therefore should be equal. The dynamic behaviour was simulated in the chemical shift range 5.0 – 3.0 ppm. The agreement between the experimental and fitted spectra is depicted in the **Figure S36:**. The obtained k_1 and k_2 values enabled determination of thermodynamic parameters and the activation free energy of the *cone-cone* interconversion using Eyring equations:

$$k = \frac{k_B T}{h} e^{-\frac{\Delta G^\ddagger}{RT}}$$

where k_B is the Boltzmann constant, R the gas constant and h the Planck constant.

The obtained values of both rate constants k_1 and k_2 and of the final overlap of the model and the corresponding experimental spectrum for each temperature are listed in **Table S2**. The dependence of ln(k/T) on 1/T served for determination of enthalpic parameters, **Figure S37** shows this dependence calculated for k_1 representing the major system of the two equivalent CH_2 bridges. **Table S3** summarized the obtained thermodynamic parameters for dynamic exchange of the two *cone* conformations of **10** calculated both rate constants k_1 and k_2. The final values of activation free energy $\Delta G^\ddagger$ were calculated for 300 K and are in good agreement.

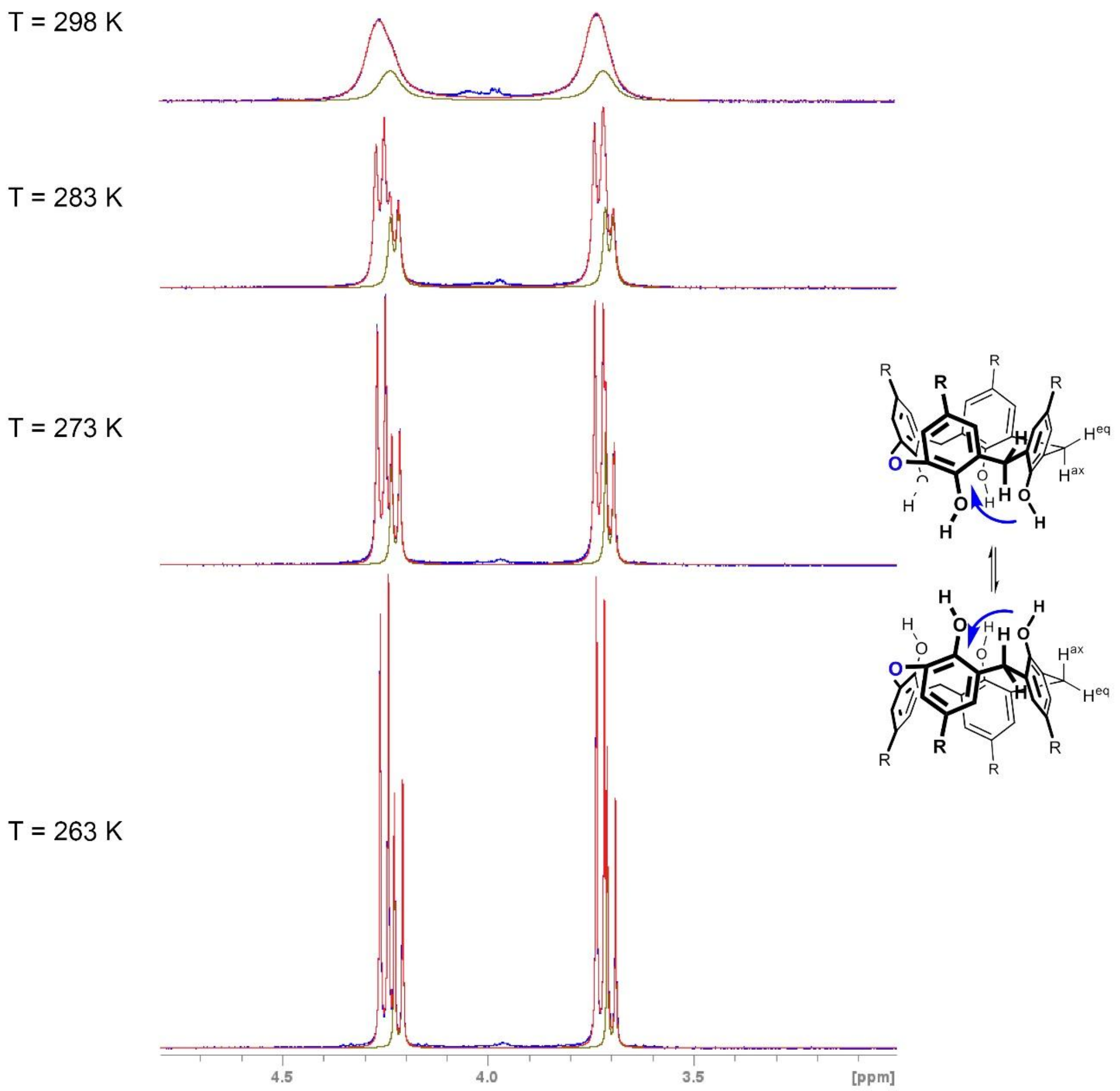

Figure S36: Spectra of the variable temperature study performed in CD_2Cl_2 (500 MHz); experimental spectrum (blue), a sum of both simulated systems (red), a contribution of the minor system 2 (green).

Table S2: The obtained values of rate constants k_1 and k_2 for each temperature and of the final overlap of the model and the corresponding experimental spectrum.

T (K)	k_1 (Hz)	k_2 (Hz)	Overlap (%)
263	0	0	94.3
273	6.4	6.5	95.1
283	18.9	18.9	95.8
298	102.4	103.6	96.2

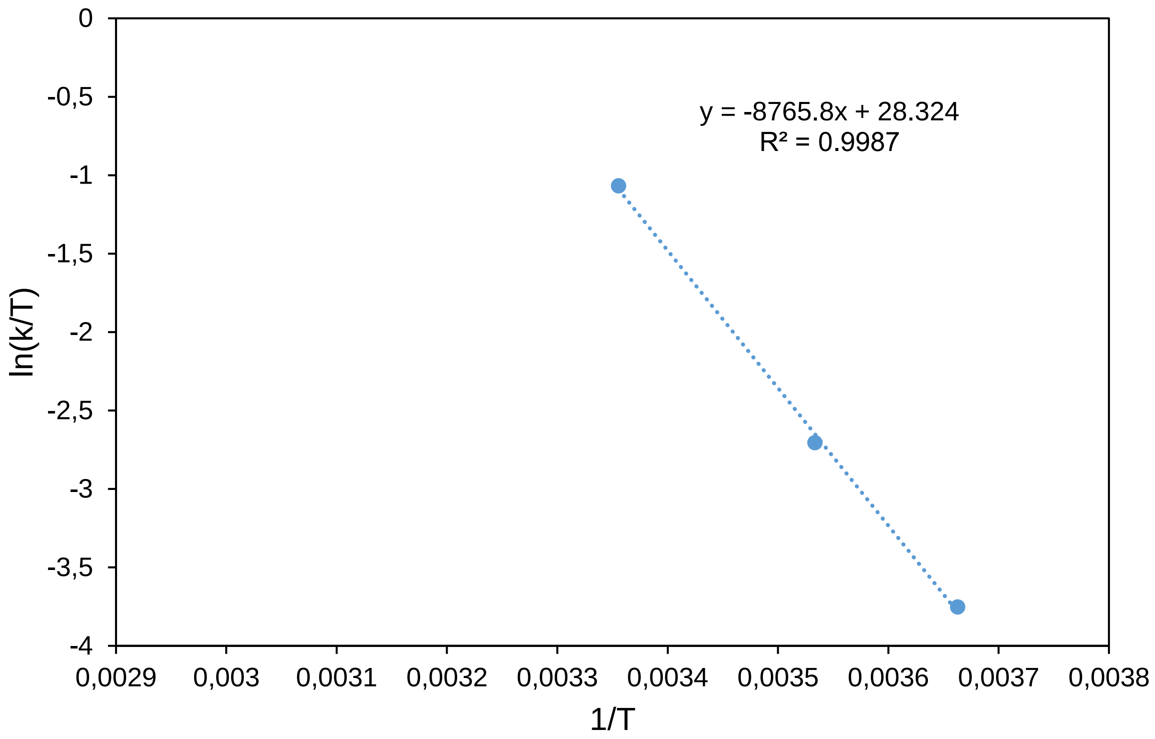

Figure S37: The dependence of ln(*k*/T) on 1/T calculated for k_1 (the major system, two equivalent CH_2 bridges).

Table S3: The obtained thermodynamic parameters for both exchanging systems

system	$\Delta H^{\ddagger}$ (kJ/mol)	$\Delta S^{\ddagger}$ (J/K·mol)	$\Delta G^{\ddagger}_{300K}$ (kJ/mol)	$\Delta G^{\ddagger}_{300K}$ (kcal/mol)
2 x CH_2 (k_1)	72.9	37.9	61.5	14.7
1 x CH_2 (k_2)	72.8	37.8	61.5	14.7

The second variable temperature study of **10** was focused on a dynamic behaviour of phenolic OH groups and was performed in CD_2Cl_2 (δ = 5.32 ppm) in the temperature range 183 – 298 K. The obtained spectra in the temperature range 203 – 243 K were then analysed in Dynamic NMR Models (dNMR) in the TopSpin (version 4.5.0) software which provided the rate constant *k* for each temperature. At the lowest temperature, the system provided four resolved OH signals at 10.35, 9.93, 9.85 and 9.68 ppm.

Increasing the temperature, a chemical exchange between two systems was observed. One system consists of two the most downfield signals (10.35 and 9.93 ppm) and the second one contains the other signals at 9.85 and 9.68 ppm. At 243 K, the pairs of signals collapse into two broad signals of the same intensity. Again, the chemical exchange of 2 two systems were fitted independently providing two rate constants k_1 and k_2, both describing the same dynamic process. The dynamic behaviour was simulated in the chemical shift range 11 – 9 ppm keeping $k_1 \cong k_2$. The agreement between the experimental and fitted spectra is depicted in the **Figure S38** together with a schematic depiction of the *flip-flow* dynamics of OH groups changing the orientation of circular hydrogen bond which is manifested in NMR spectra by a chemical exchange between two pairs of signals. Based on 2D NMR experiments performed at 183 K, the downfield system (system 1) was assigned to OH groups in the proximity of the oxa-bridge while the system 2 corresponds to OH groups opposite to the oxa-bridge. The activation free energy of the *flip-flop* motion of OH groups in molecule **10** was then calculated using Eyring equations. The obtained values of the rate constants k_1 and k_2 and of the final overlap of the model and the experiment for each temperature are listed in **Table S4**. **Figure S39** shows the dependence of ln(*k*/T) on 1/T calculated for k_1 and **Table S5** summarized the obtained thermodynamic parameters provided by both simulated systems. The final values of activation free energy $\Delta G^{\ddagger}$ were calculated for 300 K and are in good agreement for both systems.

Further increase of the temperature above 243 K led to a collapse of both systems into a single resonance at 10.02 ppm corresponding to an average signal of all four OH groups.

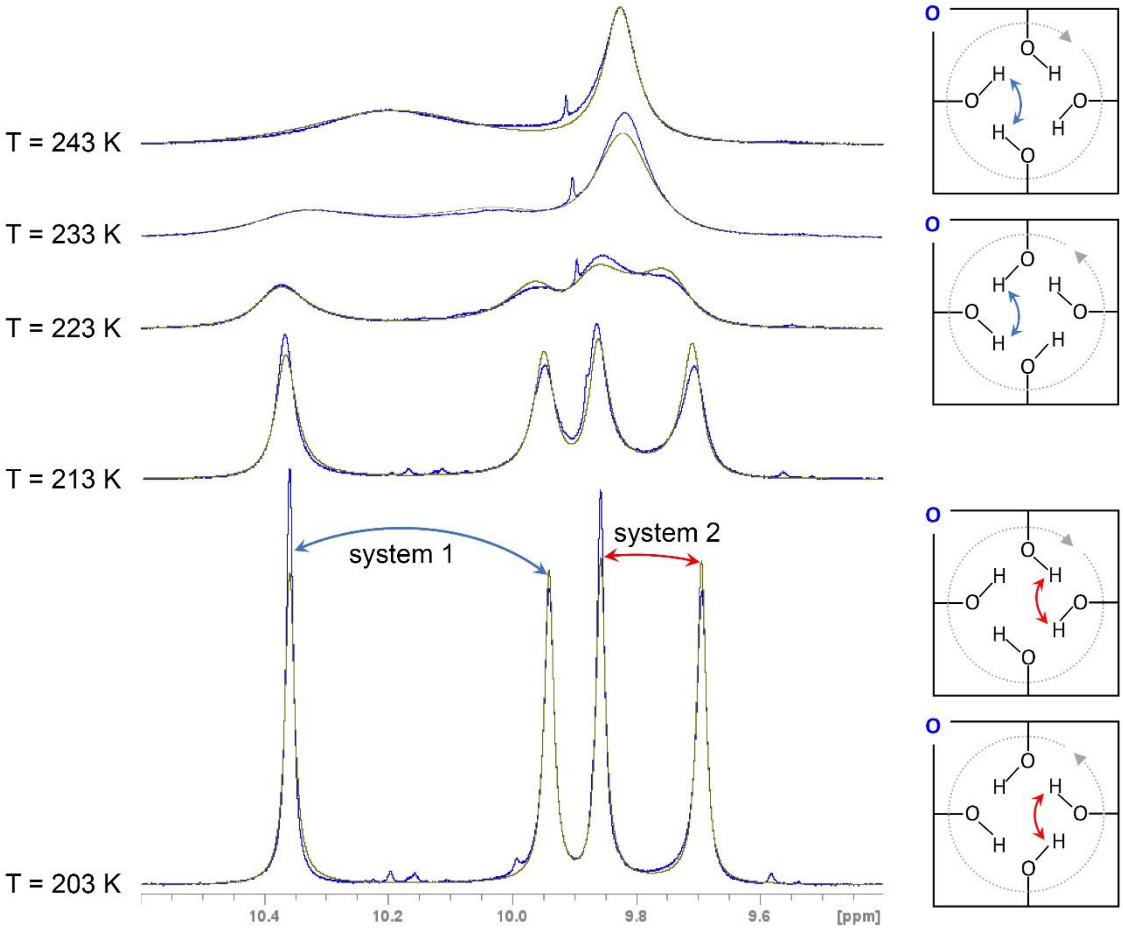

Figure S38: Spectra of the variable temperature study performed in CD_2Cl_2 (500 MHz) (left); experimental spectrum (blue), a sum of both simulated systems (grey). A schematic depiction of the *flip-flow* dynamics of OH groups when changing the orientation of circular hydrogen bond (right). The pairs of two exchanging protons are indicated by an arrow.

Table S4: The obtained values of rate constants k_1 and k_2 for each temperature and of the final overlap of the model and the corresponding experimental spectrum.

T (K)	k_1 (Hz)	k_2 (Hz)	Overlap (%)
203	13	12	93.4
213	46	43	93.8
223	140	140	95.5
233	340	360	95.1
243	649	662	94.9

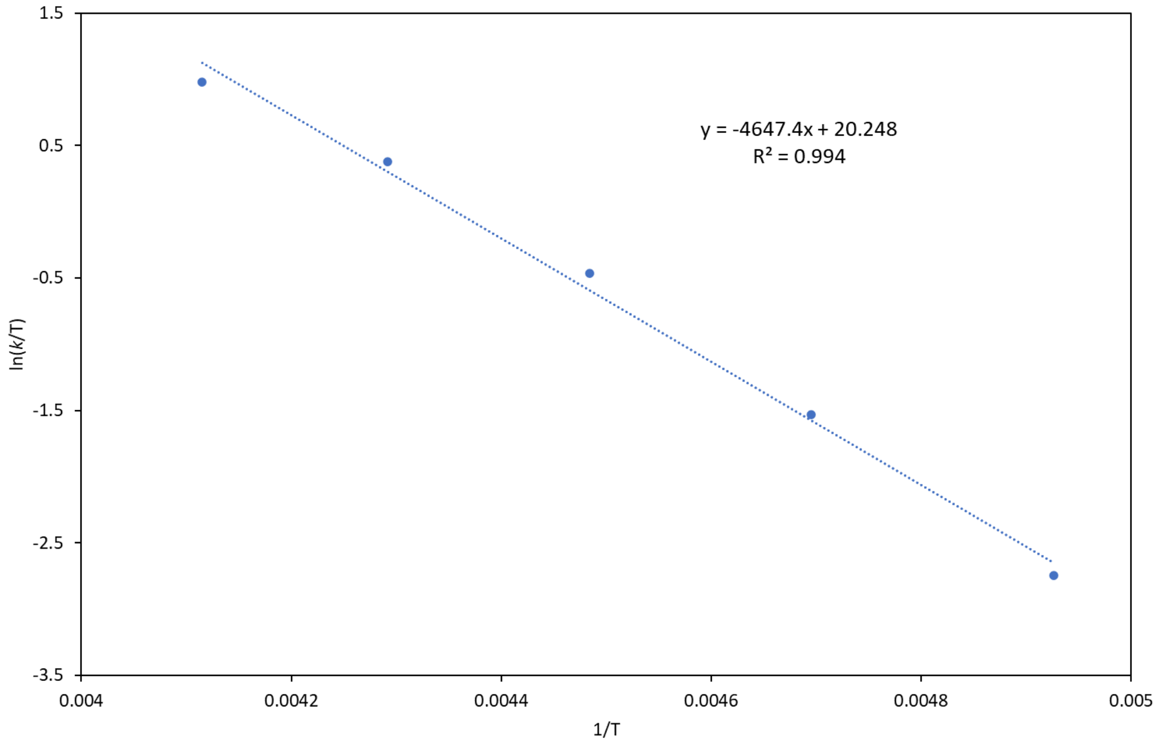

Figure S39: The dependence of ln(*k*/T) on 1/T calculated for k_1.

Table S5: The obtained thermodynamic parameters for both exchanging systems of phenolic OH groups.

system	$\Delta H^‡$ (kJ/mol)	$\Delta S^‡$ (J/K·mol)	$\Delta G^‡_{300K}$ (kJ/mol)	$\Delta G^‡_{300K}$ (kcal/mol)
10.35 → 9.93 (k_1)	38.6	-29.2	47.4	11.3
9.85 → 9.68 (k_2)	40.0	-23.3	47.0	11.2

5. Crystallographic data

The monocrystals were prepared by slow evaporation of solutions of **8** and **9** in chloroform.

Compound 10
M = 650.91 g.mol^{-1}, tetragonal system, space group $P4/n$, a = 12.7755 (4) Å, c = 12.6860 (6) Å, Z = 2, V = 2070.53 (13) Å^3, D_c = 1.044 g.cm^{-3}, μ(Cu-$K\alpha$) = 0.53 mm^{-1}, crystal dimensions of 0.14 × 0.08 × 0.04 mm. Data were collected at 180 (2) K on a Bruker D8 Venture Photon CMOS diffractometer with Incoatec microfocus sealed tube Cu-$K\alpha$ radiation. The structure was solved by dual space methods(S9) and anisotropically refined by full matrix least squares on F squared using the Olex2(S10) to final value R = 0.086 and wR = 0.203 using 1976 independent reflections (ϑ_{max} = 70.3°), 124 parameters and 13 restrains. The hydrogen atoms bonded to carbon atoms were placed in calculated positions refined with riding constraints. The hydrogen atom bonded to oxygen was refined with restrained geometry. The disordered oxygen and methylene bridge positions were found in difference electron density maps and refined with restrained APDs. To keep the molecular composition the occupancy ratio was fixed to 0.25:0.75 (O:CH_2). Highly disordered solvent was observed in the oxacalix[4]arene cavity. The solvent disorder was further complicated by the presence of fourfold axis; therefore, we have decided to remove the solvent from the structure model and calculate its contribution to structure factors using Olex2 Mask(S11). The molecular graphics were created using Diamond 3.0(S12). The structure was deposited into Cambridge Structural Database under number CCDC 2536607.

Compound 11
M = 819.22 g.mol^{-1}, triclinic system, space group $P\overline{1}$, a = 12.199 (2) Å, b = 13.780 (2) Å, c = 17.715 (3) Å, α = 78.527 (10)°, β = 71.23 (1)°, γ = 64.096 (9)°, Z = 2, V = 2530.3 (8) Å^3, D_c = 1.075 g.cm^{-3}, μ(Cu-$K\alpha$) = 0.52 mm^{-1}, crystal dimensions of 0.47 × 0.31 × 0.04 mm. The data of highly deficient weakly diffracting crystal were collected at 180 (2) K on a Bruker D8 Venture Photon CMOS diffractometer with Incoatec microfocus sealed tube Cu-$K\alpha$ radiation. The structure was solved by charge flipping methods(S13) and anisotropically refined by full matrix least squares on F squared using the CRYSTALS(S14) to final value R = 0.113 and wR = 0.321 using 5071 independent reflections (ϑ_{max} = 49.8°), 678 parameters and 149 restrains. The hydrogen atoms bonded to carbon atoms were placed in calculated positions refined with riding constraints. Two bridging positions were found to be disordered, each being occupied by a mixture of methylene and oxa bridge. The disordered bridges were refined with restrained geometry and ADPs, while their occupancies were refined with sums constrained to full in each position and to keep the correct molecular composition, resulting in the final ratio of 55(3):45(3) in position 2 and 45(3):55(3) in position 14 for C:O respectively. All four propoxy groups were found to be disordered over two positions. They were refined with restrained geometry and ADPs and sum of occupancies constrained to full in each of the groups, resulting in occupancy ratios of 751(15):149(15), 565(16):436(16), 638(14):362(14) and 744(14):256(14). MCE(S15) was used for visualization of electron density maps. The molecular graphics were created using Diamond 3.0(S12). The structure was deposited into Cambridge Structural Database under number CCDC 2536608.

Compound 12
M = 819.22 g.mol^{-1}, monoclinic system, space group $P2_1/c$, a = 12.7125 (4) Å, b = 32.7348 (12) Å, c = 13.3826 (5) Å, β = 114.705 (2)°, Z = 4, V = 5059.3 (3) Å^3, D_c = 1.075 g.cm^{-3}, μ(Cu-$K\alpha$) = 0.52 mm^{-1}, crystal dimensions of 0.36 × 0.13 × 0.08 mm. The data of highly deficient weakly diffracting crystal were collected at 180 (2) K on a Bruker D8 Venture Photon CMOS diffractometer with Incoatec microfocus sealed tube Cu-$K\alpha$ radiation. The structure was solved by charge flipping methods(S13) and anisotropically refined by full matrix least squares on F squared using the CRYSTALS(S14) to final value

R = 0.063 and wR = 0.185 using 9645 independent reflections (ϑ_{max} = 70.6°), 810 parameters and 399 restrains. The hydrogen atoms bonded to carbon atoms were placed in calculated positions refined with riding constraints. Two bridging positions were found to be disordered, each being occupied by a mixture of methylene and oxa bridge. The disordered bridges were refined with restrained geometry and ADPs, while their occupancies were refined with sums constrained to full in each position and to keep the correct molecular composition, resulting in the final ratio of 288(11):712(11) in position 2 and 712(11):288(11) in position 8 for C:O respectively. All four tert-butyl groups were found to be disordered over two positions, two propoxy groups were found to be disordered over two positions, and one propoxy group was found to be disordered over three positions. They were refined with restrained geometry and ADPs and sum of occupancies constrained to full in each of the groups, resulting in occupancy ratios of 709(15):291(15), 767(5):233(5), 842(5):158(5) and 681(6):319(6) for tert-butyl groups and 760(6):240(6), 812(6):188(6) and 505(7):313(7):183(6) for propoxy groups. MCE[(S15)] was used for visualization of electron density maps. The molecular graphics were created using Diamond 3.0[(S12)]. The structure was deposited into Cambridge Structural Database under number CCDC 2536609.

(S9) Sheldrick, G.M., *Acta Cryst.* **2015**, *A71*, 3-8.
(S10) Bourhis, L.J.; Dolomanov, O.V.; Gildea, R.J.; Howard, J.A.K.; Puschmann, H., *Acta Cryst.* **2015,** *A71*, 59-75.
(S11) Van der Sluis, P.; Spek, A. L., *Acta Cryst*. **1990**, *A46*, 194.
(S12) Brandenburg, K. (1999). DIAMOND. Crystal Impact GbR, Bonn, Germany.
(S13) Palatinus, L.; Chapuis, G., *J. Appl. Cryst.* **2007,** *40*, 786-790.
(S14) Betteridge, P.W.; Carruthers, J.R.; Cooper, R.I.; Prout, K.; Watkin, D.J., *J. Appl. Cryst.* **2003,** *36*, 1487.
(S15) Rohlicek J.; Husak M., *J. Appl. Cryst.* **2007,** *40*, 600

6. Single crystal X-ray structures

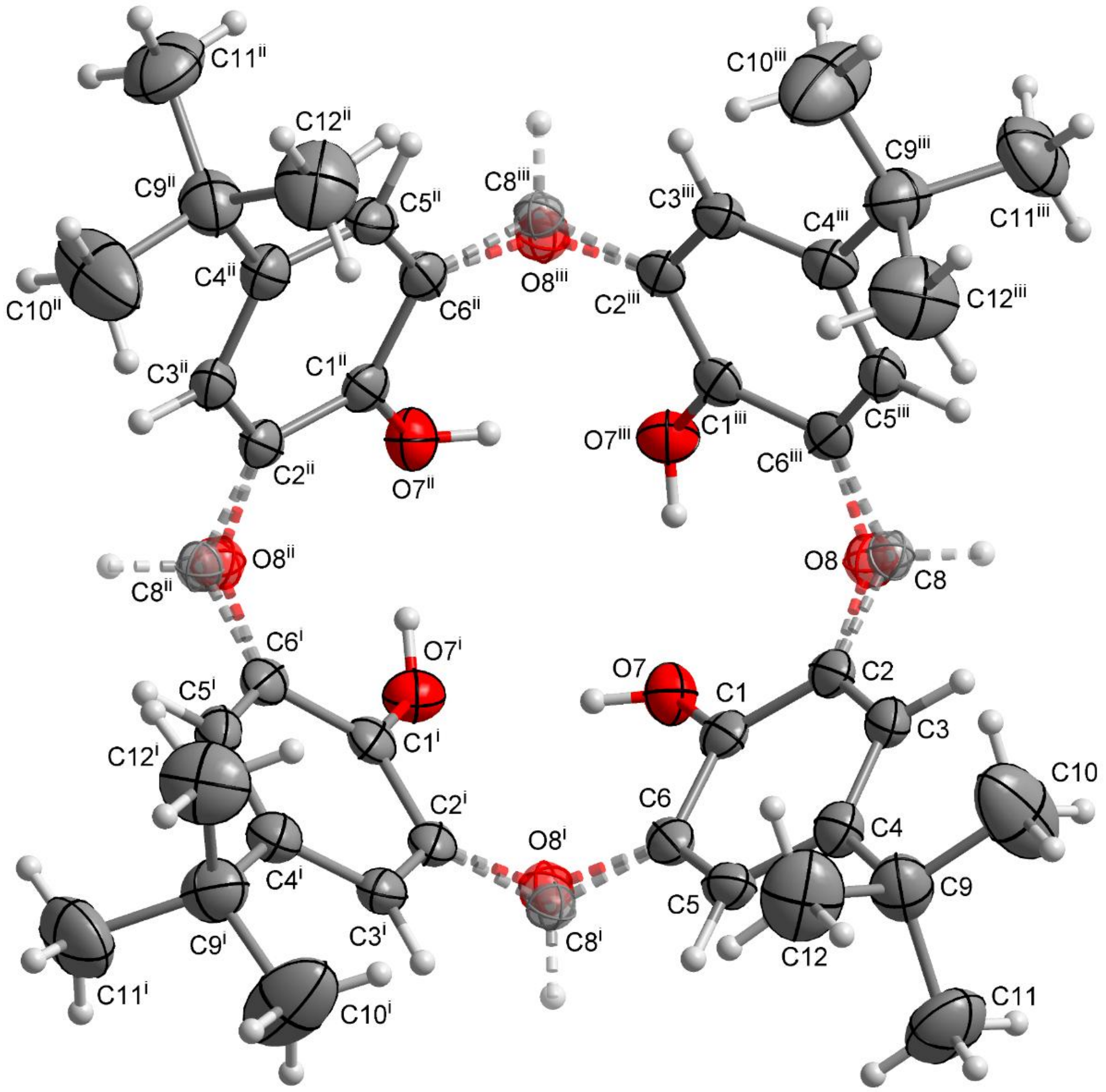

Figure S40: The numbering scheme of crystal structure **10**, with ADPs drawn at 50% probability level. The weakly occupied atoms are depicted as transparent, with dashed bonds. Symmetry codes: (*i*) 1.5-*y*, *x*, *z*; (*ii*) 1.5-*x*, 1.5-*y*, *z*; (*iii*) *y*, 1.5-*x*, *z*.

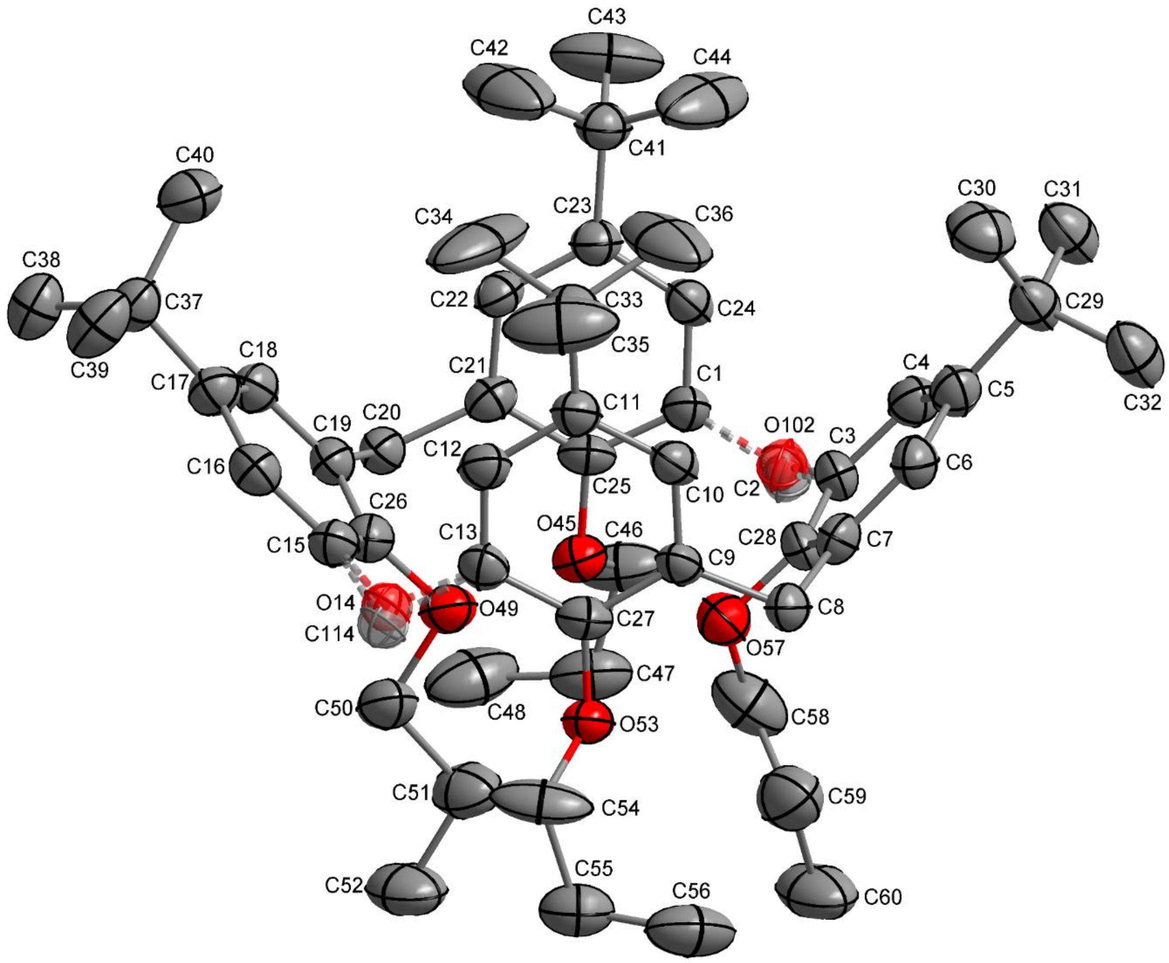

Figure S41: The numbering scheme of crystal structure **11**, with ADPs drawn at 30% probability level. The weakly occupied bridging atoms are depicted as transparent, with dashed bonds. Hydrogen atoms and weakly occupied propoxy group positions were omitted for clarity.

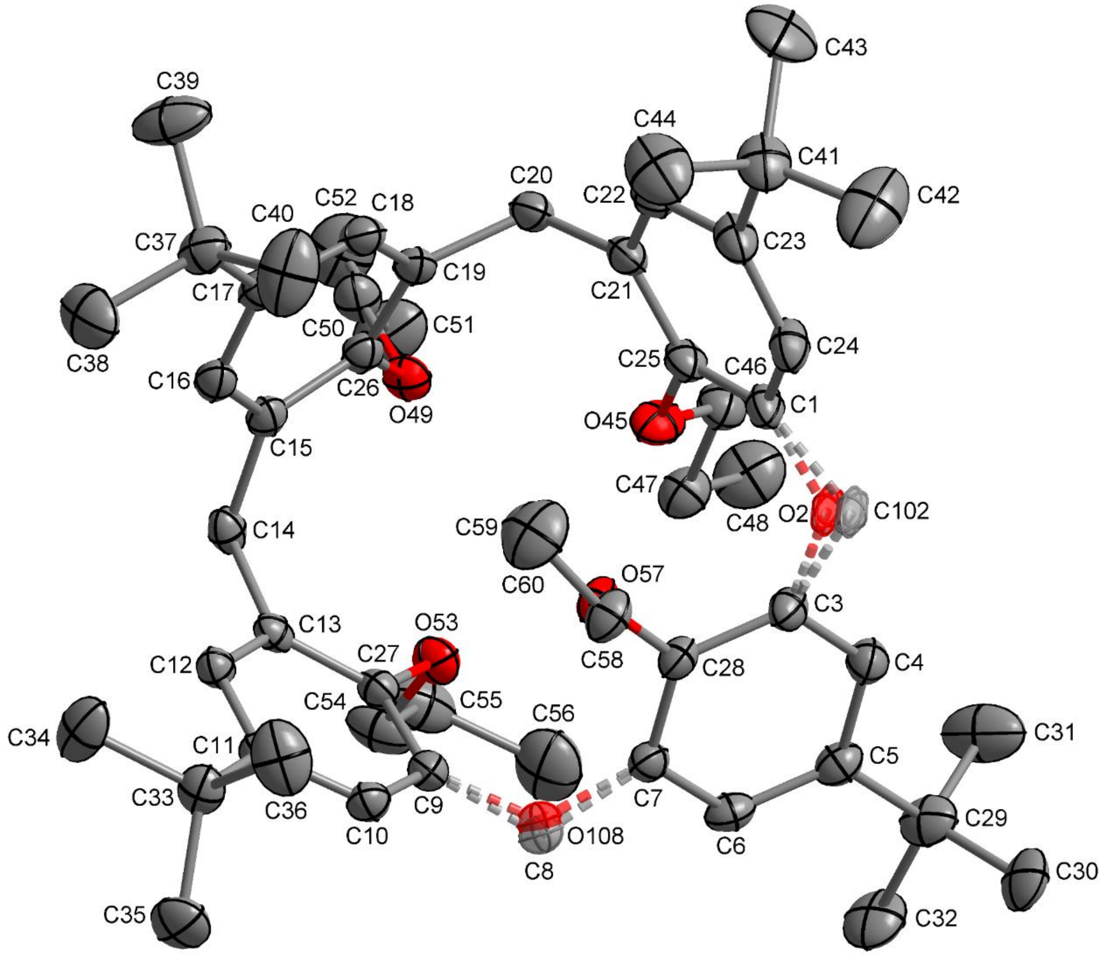

Figure S42: The numbering scheme of crystal structure **12**, with ADPs drawn at 30% probability level. The weakly occupied bridging atoms are depicted as transparent, with dashed bonds. Hydrogen atoms and weakly occupied tert-butyl and propoxy group positions were omitted for clarity.

7. Computational results – energy minimized alkylation products

DFT calculations (Gaussian 16, revision B.01) (S16) using the B3LYP functional and def2-TZVP basis set.

Compound **11** (*cone*)

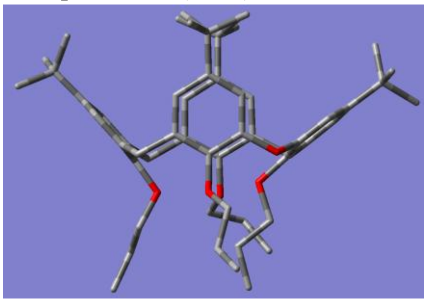 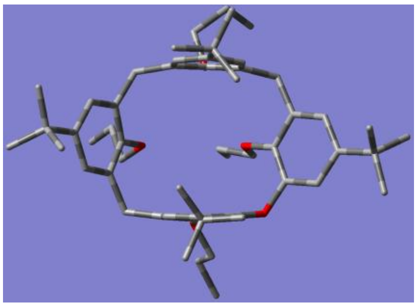

Center Number	Atom	Coordinates (Angstroms) X	Y	Z
1	C	0.081217	0.695418	2.779370
2	C	-1.153648	0.036061	2.708542
3	C	-1.168958	-1.358087	2.773141
4	C	-0.006755	-2.119168	2.859358
5	C	1.202299	-1.427702	2.784649
6	C	1.274919	-0.040218	2.722335
7	H	2.133950	-1.979794	2.764627
8	H	-2.130652	-1.849323	2.736790
9	C	-0.014481	-3.646603	3.025495
10	C	0.820894	-4.309832	1.912327
11	H	0.419681	-4.064945	0.927793
12	H	0.807481	-5.396777	2.025245
13	H	1.862193	-3.987854	1.935534
14	C	0.598273	-4.004637	4.396217
15	H	0.020059	-3.560605	5.209171
16	H	1.624345	-3.643855	4.481584
17	H	0.609553	-5.088387	4.539242
18	C	-1.432869	-4.232968	2.967400
19	H	-1.918790	-4.022549	2.012801
20	H	-2.066758	-3.843769	3.766405
21	H	-1.386437	-5.317642	3.084107
22	O	0.133633	2.073047	2.864487
23	C	2.631744	0.620485	2.510539
24	C	3.211247	0.248105	1.156585
25	H	3.327423	0.301503	3.290252
26	H	2.518014	1.698425	2.589085
27	C	2.644958	0.783456	-0.007071
28	C	3.030259	0.282768	-1.249976

29	C	4.247112	-0.672064	1.027952
30	C	4.731551	-1.097181	-0.215030
31	C	4.082963	-0.619746	-1.353052
32	H	4.371677	-0.936798	-2.344362
33	H	4.693378	-1.060475	1.935548
34	C	5.920908	-2.068084	-0.286171
35	C	5.578933	-3.372285	0.463353
36	H	5.357450	-3.189444	1.515415
37	H	6.420496	-4.067903	0.417576
38	H	4.710073	-3.861476	0.018681
39	C	7.153538	-1.412956	0.371819
40	H	6.969131	-1.166453	1.418130
41	H	7.426344	-0.490681	-0.144932
42	H	8.010378	-2.090308	0.333490
43	C	6.290459	-2.432891	-1.731524
44	H	7.143825	-3.113763	-1.730570
45	H	6.571536	-1.553079	-2.313406
46	H	5.469760	-2.935720	-2.246792
47	O	1.706186	1.772132	0.087839
48	O	2.355808	0.699142	-2.395827
49	C	1.132296	0.054330	-2.597563
50	C	-0.265025	2.629314	4.127919
51	H	-0.742603	3.585430	3.895606
52	C	-0.043006	0.810037	-2.665935
53	C	-1.270237	0.133914	-2.722344
54	C	1.099736	-1.326213	-2.751148
55	C	-0.101941	-2.007814	-2.924178
56	C	-1.271037	-1.250818	-2.865920
57	O	-0.009495	2.182894	-2.655072
58	H	-2.230903	-1.747315	-2.926377
59	H	2.039678	-1.854906	-2.722810
60	C	-0.168342	-3.520881	-3.183091
61	C	-1.090137	-4.199145	-2.150434
62	H	-0.721659	-4.039649	-1.136069
63	H	-1.132932	-5.275967	-2.331802
64	H	-2.109053	-3.814851	-2.197404
65	C	1.212670	-4.187682	-3.098213
66	H	1.664913	-4.057797	-2.113141
67	H	1.902197	-3.791877	-3.846339
68	H	1.113681	-5.259908	-3.278631
69	C	-0.733433	-3.761716	-4.599098
70	H	-1.734098	-3.340859	-4.706741
71	H	-0.794249	-4.832772	-4.808957
72	H	-0.095155	-3.302491	-5.356704
73	C	-2.597444	0.862859	-2.544716
74	C	-3.245954	0.471214	-1.223982
75	H	-3.273308	0.590816	-3.358653
76	H	-2.425099	1.933397	-2.608447

77	C	-2.778791	0.988162	-0.004552
78	C	-3.154523	0.405161	1.208372
79	C	-4.141368	-0.584719	1.189454
80	C	-4.729694	-1.034395	0.009941
81	C	-4.232598	-0.508593	-1.186961
82	H	-4.614458	-0.876706	-2.132040
83	H	-4.456466	-0.993968	2.139513
84	C	-5.864621	-2.070513	-0.017037
85	C	-5.431084	-3.306009	-0.832647
86	H	-5.190760	-3.047012	-1.864465
87	H	-6.234045	-4.047036	-0.856041
88	H	-4.549622	-3.774770	-0.391002
89	C	-7.111856	-1.442727	-0.674183
90	H	-7.930645	-2.166032	-0.707525
91	H	-7.451263	-0.571227	-0.110703
92	H	-6.909267	-1.119858	-1.696164
93	C	-6.257114	-2.545864	1.389778
94	H	-5.425474	-3.034666	1.900941
95	H	-7.070249	-3.270905	1.317619
96	H	-6.605462	-1.721376	2.014748
97	O	-1.893185	2.037070	0.000468
98	C	-2.466402	0.790773	2.506741
99	H	-3.143644	0.578897	3.338676
100	H	-2.265019	1.857180	2.507696
101	H	-1.013656	1.983464	4.593337
102	C	-2.438808	3.312728	-0.366207
103	C	-2.782741	4.167222	0.846231
104	H	-3.330372	3.172750	-0.984959
105	C	2.268238	3.080069	-0.112439
106	C	1.221543	4.132685	0.181766
107	H	2.629685	3.162767	-1.143297
108	C	0.512574	2.760669	-3.862612
109	C	0.345909	4.266975	-3.803761
110	H	-0.032872	2.349967	-4.721294
111	C	0.902321	2.845852	5.083966
112	H	-1.681131	3.804610	-0.977411
113	H	3.131479	3.201678	0.554852
114	C	1.794328	5.545692	0.081678
115	H	0.817725	3.949041	1.178464
116	H	0.399934	4.002079	-0.521766
117	H	1.567525	2.491518	-3.971813
118	H	1.024768	6.294701	0.276831
119	H	2.203292	5.746711	-0.912143
120	H	2.598322	5.704506	0.804894
121	C	0.870736	4.949647	-5.066101
122	H	0.873167	4.648667	-2.926444
123	H	-0.712634	4.502334	-3.663084
124	H	0.744507	6.031708	-5.006122

125	H	0.340366	4.603788	-5.956624
126	H	1.934192	4.748200	-5.214785
127	C	-3.286319	5.551826	0.439849
128	H	-1.892042	4.257361	1.472214
129	H	-3.544100	3.656961	1.442675
130	H	-3.524995	6.156638	1.316212
131	H	-4.190676	5.484272	-0.170101
132	H	-2.535477	6.093430	-0.140887
133	H	0.480270	3.108418	6.060181
134	C	1.875531	3.935254	4.639449
135	H	1.427003	1.896405	5.223360
136	H	2.701305	4.038927	5.345784
137	H	2.300444	3.713971	3.659814
138	H	1.373430	4.903788	4.571050

HF = -2519.8658327

Compound **12** (*partial cone A*)

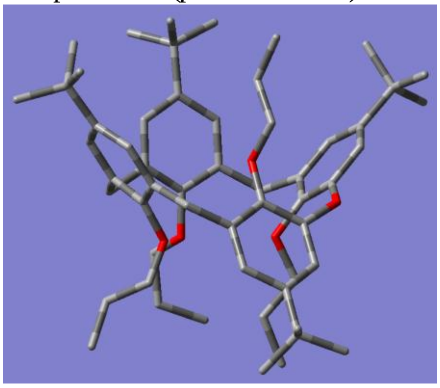
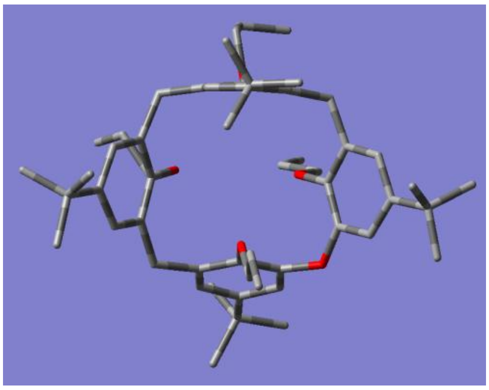

Center Number	Atom	Coordinates (Angstroms) X	Y	Z
1	C	-2.356745	5.212255	-2.009584
2	C	-5.221325	0.166969	0.824873
3	C	5.969629	-0.452969	-1.689525
4	C	-2.418520	-5.468033	-1.348915
5	O	-0.080237	0.169429	3.330932
6	O	1.383834	1.923818	0.814813
7	O	0.245750	-0.144722	-1.916321
8	O	1.232318	-1.711217	0.973911
9	C	-0.375777	-3.918249	-1.483199
10	C	0.506012	-2.999971	-0.917205
11	C	0.329597	-2.568238	0.398017
12	C	-0.801402	-2.994103	1.109724
13	C	-1.657046	-3.921841	0.524022
14	C	-1.459024	-4.417106	-0.768897
15	O	1.582057	-2.661005	-1.709697
16	C	2.251874	-1.451502	-1.676560
17	C	3.639444	-1.536206	-1.634590
18	C	4.433712	-0.396915	-1.696084
19	C	3.774563	0.831369	-1.785741
20	C	1.612187	-0.218538	-1.829018
21	C	-1.135785	-2.387095	2.463120
22	C	-1.857184	-1.060013	2.279704
23	C	-1.245904	0.158556	2.585029
24	C	-3.080625	1.322598	1.569347
25	C	-3.791662	0.135108	1.386771
26	C	-3.128855	-1.045099	1.704583
27	C	-1.810494	1.361047	2.134500
28	C	2.387712	0.947995	-1.855972
29	C	1.793331	2.325170	-2.101847
30	C	-1.383322	4.257306	-1.300685
31	C	-1.567242	3.935225	0.045560

32	C	-0.689480	3.116162	0.747842
33	C	0.464387	2.650563	0.094890
34	C	0.634513	2.865657	-1.275862
35	C	-0.295335	3.672240	-1.939542
36	C	-2.041396	-5.876787	-2.780507
37	C	-2.384276	-6.732833	-0.465635
38	C	-3.855015	-4.906798	-1.375306
39	C	-6.112883	1.029582	1.742320
40	C	-5.213104	0.775446	-0.592080
41	C	-5.846598	-1.233353	0.738641
42	C	-2.008421	5.409334	-3.492561
43	C	-3.794571	4.660736	-1.928956
44	C	-2.305326	6.591419	-1.318896
45	C	6.499898	-1.888523	-1.560092
46	C	6.506082	0.137504	-3.010722
47	C	6.522109	0.369292	-0.507444
48	C	-0.309507	0.499720	4.708644
49	C	2.620849	2.581156	1.108280
50	C	-0.306254	-0.454717	-3.204142
51	C	2.331711	-2.359860	1.625898
52	C	-1.815519	-0.345386	-3.116050
53	C	-2.491845	-0.633191	-4.455173
54	C	2.551905	3.495095	2.325847
55	C	3.899476	4.157028	2.612006
56	C	3.278825	-1.300844	2.152906
57	C	4.459343	-1.910753	2.906484
58	C	0.979789	0.351362	5.496475
59	C	1.431198	-1.094088	5.692768
60	H	-0.164350	-4.237451	-2.493145
61	H	-2.505733	-4.263338	1.103396
62	H	4.065796	-2.525602	-1.558216
63	H	4.356350	1.743837	-1.831066
64	H	-1.770848	-3.085673	3.012559
65	H	-0.234622	-2.225144	3.047696
66	H	-3.519069	2.258269	1.246925
67	H	-3.596150	-1.997394	1.498867
68	H	1.463350	2.360622	-3.144527
69	H	2.622304	3.037826	-2.049374
70	H	-2.423825	4.330949	0.577770
71	H	-0.138061	3.842347	-2.996165
72	H	-2.747149	-6.625562	-3.145508
73	H	-2.076086	-5.029185	-3.467685
74	H	-1.042449	-6.314197	-2.828224
75	H	-3.064093	-7.491578	-0.861455
76	H	-2.685159	-6.517080	0.560194
77	H	-1.379603	-7.159181	-0.435744
78	H	-3.911802	-4.011222	-1.997185
79	H	-4.545171	-5.648565	-1.784970

80	H	-4.205703	-4.643408	-0.376938
81	H	-7.134829	1.065012	1.356267
82	H	-6.145730	0.617112	2.752865
83	H	-5.747717	2.054804	1.813106
84	H	-6.227633	0.809360	-0.997781
85	H	-4.595955	0.178844	-1.266318
86	H	-4.818710	1.791677	-0.593480
87	H	-5.894401	-1.716545	1.716615
88	H	-6.866996	-1.155833	0.357677
89	H	-5.292394	-1.885941	0.061756
90	H	-2.723183	6.097547	-3.948154
91	H	-1.011930	5.835437	-3.623240
92	H	-2.054467	4.471133	-4.049057
93	H	-4.490287	5.338250	-2.430336
94	H	-4.128886	4.549360	-0.896896
95	H	-3.865526	3.684021	-2.411198
96	H	-1.300543	7.015499	-1.371325
97	H	-2.994115	7.287485	-1.804462
98	H	-2.583999	6.524797	-0.266421
99	H	7.591605	-1.877288	-1.559187
100	H	6.174341	-2.359687	-0.630672
101	H	6.177905	-2.517276	-2.392197
102	H	7.598413	0.104133	-3.026680
103	H	6.201534	1.176632	-3.141134
104	H	6.136350	-0.428615	-3.867906
105	H	6.174746	-0.034811	0.445394
106	H	7.614664	0.344795	-0.506502
107	H	6.213736	1.413955	-0.560474
108	H	-1.086491	-0.159903	5.115549
109	H	-0.681859	1.527871	4.780668
110	H	3.339351	1.778357	1.277841
111	H	2.960900	3.152534	0.238626
112	H	-0.005651	-1.464308	-3.503641
113	H	0.091180	0.247044	-3.948158
114	H	1.953267	-2.987342	2.443022
115	H	2.844237	-3.020035	0.916268
116	H	-2.072662	0.657548	-2.767302
117	H	-2.168737	-1.043595	-2.353775
118	H	-3.576617	-0.552156	-4.369861
119	H	-2.263361	-1.641050	-4.810584
120	H	-2.168899	0.070399	-5.226733
121	H	2.232967	2.907446	3.190182
122	H	1.790527	4.262284	2.161611
123	H	3.843254	4.799080	3.492501
124	H	4.224956	4.776625	1.772637
125	H	4.678493	3.412599	2.795051
126	H	2.715292	-0.624637	2.798138
127	H	3.633799	-0.707190	1.308456

128	H	5.132924	-1.134245	3.273136
129	H	5.043066	-2.576812	2.265941
130	H	4.127684	-2.492137	3.770302
131	H	0.821950	0.821565	6.472220
132	H	1.762685	0.929147	4.997116
133	H	2.361005	-1.141373	6.262757
134	H	1.599959	-1.588324	4.736448
135	H	0.677725	-1.668129	6.237898
136	C	-1.030428	2.670670	2.160329
137	H	-0.121551	2.535062	2.737170
138	H	-1.626742	3.448790	2.643205

HF = -2519.868249

Compound **13** (*partial cone B*)

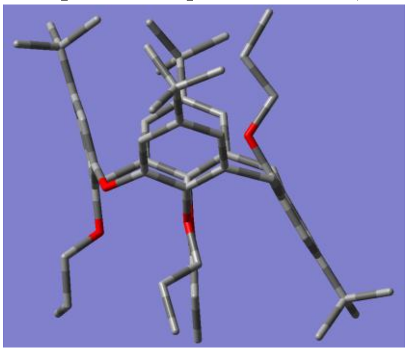
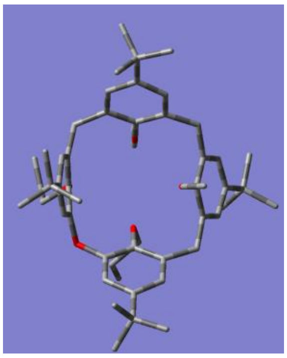

Center Number	Atom	Coordinates (Angstroms) X	Y	Z
1	C	-2.356745	5.212255	-2.009584
2	C	-5.221325	0.166969	0.824873
1	C	-1.965153	5.556764	-1.652811
2	C	-5.170839	0.421054	0.908830
3	C	5.969710	-0.571251	-1.608404
4	C	-2.995545	-5.200529	-1.380241
5	O	0.066085	0.078636	3.198606
6	O	1.378542	1.571157	0.736923
7	O	0.272507	-0.175672	-2.112734
8	O	1.188992	-2.035144	0.908094
9	C	-0.783101	-3.896938	-1.553724
10	C	0.255133	-3.130732	-1.014017
11	C	0.162980	-2.742045	0.322777
12	C	-0.987941	-3.028802	1.074241
13	C	-1.972715	-3.826211	0.500870
14	C	-1.891492	-4.293985	-0.813006
15	C	1.456841	-2.852243	-1.904232
16	C	2.228726	-1.545603	-1.829795
17	C	3.618902	-1.618975	-1.701328
18	C	4.437483	-0.495101	-1.704574
19	C	3.802474	0.742262	-1.816687
20	C	1.640572	-0.286737	-1.979769
21	C	-1.217126	-2.398540	2.439819
22	C	-1.848422	-1.023176	2.269538
23	C	-1.123264	0.145245	2.522398
24	C	-2.928159	1.429429	1.542564
25	C	-3.728097	0.291956	1.419566
26	C	-3.140318	-0.923644	1.747252
27	C	-1.639729	1.359916	2.044292
28	O	-0.842254	2.508953	2.067680
29	C	2.422681	0.876870	-1.942139
30	C	1.852886	2.271889	-2.134011

31	C	-1.095608	4.433023	-1.067964
32	C	-1.320691	3.984206	0.237006
33	C	-0.538143	2.989948	0.795561
34	C	0.564787	2.464670	0.101550
35	C	0.756635	2.828376	-1.232845
36	C	-0.080433	3.806971	-1.783458
37	C	-2.739500	-5.590790	-2.843615
38	C	-3.067848	-6.496620	-0.545807
39	C	-4.356898	-4.478926	-1.312205
40	C	-5.959452	1.372421	1.832649
41	C	-5.166594	0.992575	-0.523895
42	C	-5.902567	-0.928762	0.882869
43	C	-1.590926	5.891352	-3.104265
44	C	-3.450069	5.140145	-1.627439
45	C	-1.783024	6.832834	-0.804464
46	C	6.477083	-2.014648	-1.472898
47	C	6.592166	0.035084	-2.883848
48	C	6.459930	0.223433	-0.380668
49	C	0.079983	0.800728	4.442050
50	C	2.640003	2.082065	1.185739
51	C	-0.231026	-0.220532	-3.449767
52	C	2.170747	-2.848254	1.554248
53	H	-0.690295	-4.200793	-2.587741
54	H	-2.835869	-4.077880	1.105198
55	H	2.185316	-3.653325	-1.743427
56	H	1.116260	-2.991451	-2.934534
57	H	4.057134	-2.602997	-1.605792
58	H	4.397490	1.647870	-1.815356
59	H	-1.880927	-3.042937	3.020760
60	H	-0.284534	-2.298049	2.986595
61	H	-3.295330	2.395361	1.228222
62	H	-3.686147	-1.842253	1.590759
63	H	1.469999	2.348285	-3.155877
64	H	2.699600	2.964920	-2.102794
65	H	-2.116243	4.396851	0.842309
66	H	0.094955	4.082720	-2.814558
67	H	-3.546119	-6.236366	-3.196733
68	H	-2.707134	-4.717169	-3.497631
69	H	-1.803212	-6.139754	-2.960359
70	H	-3.851850	-7.154274	-0.929869
71	H	-3.289078	-6.289534	0.501938
72	H	-2.120521	-7.038043	-0.585379
73	H	-4.337810	-3.555746	-1.894794
74	H	-5.146937	-5.117947	-1.714627
75	H	-4.628888	-4.222504	-0.287864
76	H	-6.988963	1.477383	1.481009
77	H	-5.986991	0.988599	2.854506
78	H	-5.515038	2.367972	1.863319

79	H	-6.189259	1.093265	-0.896592
80	H	-4.619472	0.334873	-1.201877
81	H	-4.697990	1.976380	-0.565400
82	H	-5.948767	-1.383073	1.874628
83	H	-6.928183	-0.782816	0.537745
84	H	-5.425313	-1.637064	0.203770
85	H	-2.230089	6.697313	-3.470062
86	H	-0.555789	6.227076	-3.190188
87	H	-1.729067	5.035353	-3.767681
88	H	-4.074715	5.934599	-2.043171
89	H	-3.798535	4.943479	-0.612854
90	H	-3.612088	4.236781	-2.218681
91	H	-0.742060	7.162033	-0.815174
92	H	-2.400145	7.643802	-1.199310
93	H	-2.070261	6.670575	0.235195
94	H	7.566990	-2.015056	-1.408224
95	H	6.093763	-2.497646	-0.572013
96	H	6.198336	-2.626133	-2.333123
97	H	7.683112	-0.009558	-2.833946
98	H	6.306606	1.079522	-3.014932
99	H	6.270017	-0.512549	-3.771785
100	H	6.048109	-0.190404	0.541874
101	H	7.549939	0.183149	-0.312290
102	H	6.169438	1.273161	-0.434346
103	C	-1.737960	-0.054977	-3.401118
104	H	0.036525	-1.174515	-3.920636
105	H	0.225557	0.577092	-4.049308
106	C	-2.368629	-0.085806	-4.791956
107	H	-1.966968	0.888545	-2.900044
108	H	-2.152756	-0.850145	-2.777217
109	H	-3.451406	0.034642	-4.732153
110	H	-2.170246	-1.032316	-5.301226
111	H	-1.982665	0.717209	-5.425095
112	H	3.199023	1.196831	1.488588
113	C	2.544364	3.075341	2.336054
114	H	3.175185	2.540283	0.346875
115	C	3.928128	3.547982	2.781500
116	H	2.018519	2.607055	3.168477
117	H	1.943452	3.934824	2.028501
118	H	3.853098	4.255893	3.608540
119	H	4.461237	4.047172	1.968288
120	H	4.547303	2.712176	3.117371
121	H	1.692511	-3.461187	2.329527
122	C	3.230480	-1.949886	2.160940
123	H	2.620245	-3.538082	0.828180
124	C	4.305139	-2.744753	2.900214
125	H	2.739216	-1.247508	2.836740
126	H	3.678603	-1.357124	1.360671

127	H	5.060132	-2.081280	3.325336
128	H	4.818506	-3.442251	2.233290
129	H	3.879221	-3.326780	3.721262
130	C	1.305729	0.399401	5.242421
131	H	-0.833403	0.564709	5.001827
132	H	0.084496	1.873985	4.238659
133	H	1.376247	1.085673	6.092389
134	H	2.196457	0.572034	4.632054
135	C	1.283743	-1.042201	5.745895
136	H	2.181321	-1.269968	6.324080
137	H	1.230883	-1.749033	4.917699
138	H	0.419057	-1.219017	6.390568

HF = -2519.858713

(S16) Frisch, M. J.; Trucks, G. W.; Schlegel, H. B.; Scuseria, G. E.; Robb, M. A.; Cheeseman, J. R.; Scalmani, G.; Barone, V.; Petersson, G. A.; Nakatsuji, H.; Li, X.; Caricato, M.; Marenich, A. V.; Bloino, J.; Janesko, B. G.; Gomperts, R.; Mennucci, B.; Hratchian, H. P.; Ortiz, J. V.; Izmaylov, A. F.; Sonnenberg, J. L.; Williams; Ding, F.; Lipparini, F.; Egidi, F.; Goings, J.; Peng, B.; Petrone, A.;

Henderson, T.; Ranasinghe, D.; Zakrzewski, V. G.; Gao, J.; Rega, N.; Zheng, G.; Liang, W.; Hada, M.; Ehara, M.; Toyota, K.; Fukuda, R.; Hasegawa, J.; Ishida, M.; Nakajima, T.; Honda, Y.; Kitao, O.; Nakai, H.; Vreven, T.; Throssell, K.; Montgomery Jr., J. A.; Peralta, J. E.; Ogliaro, F.; Bearpark, M. J.; Heyd, J. J.; Brothers, E. N.; Kudin, K. N.; Staroverov, V. N.; Keith, T. A.; Kobayashi, R.; Normand, J.; Raghavachari, K.; Rendell, A. P.; Burant, J. C.; Iyengar, S. S.; Tomasi, J.; Cossi, M.; Millam, J. M.; Klene, M.; Adamo, C.; Cammi, R.; Ochterski, J. W.; Martin, R. L.; Morokuma, K.; Farkas, O.; Foresman, J. B.; Fox, D. J. *Gaussian 16 Rev. C.01*, Wallingford, CT, 2016.
